# Comparative safety of interleukin inhibitors for rheumatoid arthritis: a systematic review and network meta-analysis of randomized controlled trials

**DOI:** 10.3389/fimmu.2026.1866093

**Published:** 2026-09-23

**Authors:** Changyi Liu, Jichong Li, Can Huang, Bing Wang

**Affiliations:** 1 The First Affiliated Hospital of Dalian Medical University, Dalian, Liaoning, China; 2 The Second Affiliated Hospital of Dalian Medical University, Dalian, Liaoning, China; 3 Dalian Medical University College of Basic Medical Sciences, Dalian, China

**Keywords:** adverse event, interleukin inhibitor, network meta-analysis, randomized controlled trial, rheumatoid arthritis

## Abstract

**Background:**

The safety of biological agents in the treatment of rheumatoid arthritis (RA) is an important factor affecting patient prognosis and treatment adherence. Among them, interleukin inhibitors are an important category. However, the safety of different types of interleukin inhibitors used to treat RA has not been adequately evaluated. The purpose of this study is to evaluate the safety of interleukin inhibitors for the treatment of RA.

**Methods:**

Computer-based searches were conducted in PubMed, the Cochrane Library, Embase, and CNKI to retrieve randomized controlled trials of interleukin inhibitors for the treatment of RA up to March 2026. Literature screening, data extraction, and risk of bias assessment were independently performed by two researchers, and the data were analyzed using Stata 13.1 and the R 4.5.3’s gemtc package. The network meta-analysis was performed using a Markov Chain Monte Carlo (MCMC) consistent model. Odds ratios (OR) were used as the measure of effect size.

**Results:**

A total of 24 RCTs involving 12,915 patients with RA were included. The use of interleukin inhibitors was associated with the occurrence of adverse events (AE) (OR = 1.15, 95% CI [1.09, 1.21], p < 0.0001), serious adverse events (SAEs) (OR = 1.22, 95% CI [1.08, 1.38], p = 0.001), infections (OR = 1.10, 95% CI [1.02, 1.18], p = 0.007), serious infections (OR = 1.35, 95% CI [1.00, 1.83], p = 0.046), AEs leading to discontinuation (OR = 1.65, 95% CI [1.32, 2.06], p < 0.0001), general disorders and administration site conditions (OR = 1.85, 95% CI [1.49, 2.29], p < 0.0001), gastrointestinal AEs (OR = 1.38, 95% CI [1.04, 1.83], p = 0.002), neurological AEs (OR = 1.32, 95% CI [1.03, 1.68], p = 0.024), skin and subcutaneous tissues AEs (OR = 1.86, 95% CI [1.52, 2.28], p < 0.0001), abnormal laboratory investigations (OR = 1.99, 95% CI [1.59, 2.48], p < 0.0001), and increased ALT in laboratory investigations (OR = 1.88, 95% CI [1.36, 2.60], p < 0.0001). However, interleukin inhibitor use was associated with a decrease in musculoskeletal and connective tissue AEs (OR = 0.77, 95% CI [0.62, 0.95], p = 0.014). It is worth noting that characteristics of the included studies may have affected the results, such as sparse networks, short trial durations, rare-event uncertainty, heterogeneity in background MTX/DMARD use, and lack of long-term safety data.

**Conclusions:**

Overall, the AEs caused by different interleukin inhibitors vary. However, in clinical practice, our conclusion should be interpreted cautiously according to individual patient circumstances.

**Systematic review registration:**

https://www.crd.york.ac.uk/PROSPERO/ **, identifier 420261338669.**

## 1 Introduction

Rheumatoid arthritis (RA) is a chronic and progressive autoimmune disease mainly characterized by joint lesions (1). The core pathological changes are chronic synovitis driven by synovial fibroblasts, including synovial hyperplasia, pannus formation, and joint destruction (2). The pathogenic factors underlying RA are complex and unknown. Currently, RA is believed to be related to factors such as immunity, genetics, environment, and infection (3). Its clinical manifestations are diverse, and the disease predominantly affects women. The main symptoms include symmetrical joint deformities and pain, morning stiffness lasting more than 60 minutes, and associated extra-articular organ lesions (4, 5). Rheumatoid factor (RF) and anti-cyclic citrullinated peptide antibody (ACCP) are characteristic antibodies of RA (1, 3). According to the research on Global Disease Burden, in 2020, 176 million people had RA, and the number is on the rise, representing a significant public health burden (6).

The biological agents currently used in clinical practice alleviate RA by inhibiting interleukins, tumor necrosis factors, and Janus kinases (7). Among them, the cytokines secreted by inflammatory cells, such as IL-6 and IL-1, play a significant role in the pathogenesis of RA. This has led to the development of a variety of monoclonal antibodies that treat RA by inhibiting the secretion of interleukins, and they have also passed clinical trials (8). Tocilizumab (TCZ) is a monoclonal antibody targeting the IL-6 receptor. It has been shown to be effective in treating RA, especially in patients who do not respond adequately to MTX or TNF-α inhibitors (9). Anakinra (ANA) is a monoclonal antibody targeting the IL-1 receptor, and it has moderate anti-inflammatory effects and long-term joint-protective effects (10). Olokizumab (OKZ) is a humanized monoclonal antibody targeting IL-6 that directly neutralizes IL-6. It has been approved in Russia for the treatment of RA (11). Sarilumab (SAR) is a fully human monoclonal antibody targeting the IL-6 receptor that can simultaneously control the complications of RA and extrajoint manifestations (12). Although biological agents have good efficacy, their safety profiles cannot be ignored (4). The use of JAK inhibitors is often associated with the occurrence of malignant tumors, and the use of TNF-α inhibitors increases the risk of tuberculosis (13, 14). At present, the majority of studies directly compare interleukin inhibitors with methotrexate (MTX), and comparisons among different interleukin inhibitors remain limited, highlighting an advantage of network meta-analysis. Therefore, this systematic review and network meta-analysis aimed to analyze the safety of four interleukin inhibitors, used alone or in combination with MTX, at different doses for the treatment of adult rheumatoid arthritis and to provide suggestions for improving the prognosis of patients with different systemic complications.

## 2 Materials and methods

### 2.1 Database retrieval strategy and registration

Randomized controlled trials (RCTs) of interleukin inhibitors for the treatment of RA were searched in Pubmed, Embase, the Cochrane library, and the CNKI database. The MeSH terms retrieved included RA, interleukin inhibitor, and randomized controlled trial, with searches conducted through June 2026 (**Supplementary Table S1**). This meta-analysis was performed strictly according to the Preferred Reporting Items for Systematic Reviews and Meta-Analyses for NMA guidelines (15) (**Supplementary Table S2**). This network meta-analysis was registered with PROSPERO (CRD: 420261338669).

### 2.2 Inclusion and exclusion criteria

Inclusion criteria were as follows: a. the study design was an RCT; b. participants were adults with RA diagnosed according to ACR or EULAR criteria; c. the interventions were interleukin inhibitors approved for the treatment of RA, administered alone or in combination with MTX; d. the control measures were placebo or MTX; e. the outcome indicators were various adverse events (AEs); and f. the study was published in English. Exclusion criteria were as follows: a. systematic reviews, cohort studies, animal experiments, long-term follow-up results, and studies for which the full text was not available; b. studies involving patients with juvenile RA; c. studies of unlisted interleukin inhibitors or biologically derived preparations that have not been clinically approved for RA treatment; and d. studies in which the outcome indicators were radiological reports, biomarkers, blood biochemical indicators, or pharmacokinetics, or in which no AEs were reported.

### 2.3 Study selection and data extraction

EndNote X9 was used to remove duplicates, after which records were screened based on their titles and abstracts. Further screening was performed according to inclusion and exclusion criteria. Two authors extracted basic study information liter(first author, year, diagnostic method, sex, age, course of disease, Disease Activity Score in 28 Joints [DAS28], and reported outcomes), intervention methods (drugs, doses, and course of treatment), and outcome indicators (AEs, serious adverse events [SAEs], infections, serious infections, etc.). A third author decided when there was inconsistency.

### 2.4 Risk of bias and quality assessment

Grading of Recommendations Assessment, Development and Evaluation (GRADE) was used to classify the level of evidence as high, moderate, low, or very low (16). The risk of bias was evaluated according to the ‘overall’ in Cochrane RoB 2.0. Inconsistency was assessed based on unexplained heterogeneity in the direction or size of the results across different studies. Indirectness was evaluated based on whether there were differences in the population, control, intervention, and outcomes between the research evidence and the target clinical problem. Imprecision was assessed based on excessively wide confidence intervals for the effect estimates. Funnel plots were used to assess publication bias.

The risk of bias was assessed by two independent researchers (Liu and Li) using the Cochrane risk of bias assessment tool Risk of Bias 2.0 (RoB 2.0) (17). Each project was rated as low risk, with some concerns, or high risk. When there was disagreement, the third author decided. The evaluation contents included the randomization process, deviations from intended interventions, missing outcome data, measurement of the outcome, selection of the reported result, and overall.

### 2.5 Data analysis

Stata 13.1 was used for meta-analysis of direct comparison and comparison-correction funnel plot. Odds ratio (OR) was used for the combined effect size for dichotomous variables. The chi-square test was used to assess heterogeneity among the included studies. When I^2^ > 50% and p < 0.10, heterogeneity was considered significant, and the random-effect model was used to combine the effect estimates. When I^2^ < 50% and p > 0.10, heterogeneity was considered non-significant, and the fixed-effect model was used to combine the effect estimates. Sensitivity analysis was performed using the Leave-One-Out Influence Analysis method. After each study was removed step by step, network meta-analysis was performed again to assess the combined effect size and the changes in I ^2^ and τ ^2^.

The gemtc package of R 4.5.3 was used to draw a network diagram, perform comparisons among interventions, and calculate the probability ranking. A network meta-analysis was performed using a Markov Chain Monte Carlo (MCMC) consistent model. Four chains were used for the simulations. The number of iterations for the analysis of severe infections and deaths was set to 80,000 (the first 40,000 times for burn-in and the last 40,000 times for sampling). The number of iterations for the analysis of other outcome was set to 150,000 (the first 90,000 times for burn-in and the last 60,000 times for sampling). All data were analyzed using a random-effects model with the OR as the effect size. They were estimated and inferred under the assumption that MCMC reached a stable state of convergence, as evaluated by the potential scale reduction factor (PSRF). The convergence diagnosis diagram, density diagram, and trajectory diagram were used to assess the convergence of the model. The node-split method was used to evaluate the consistency of direct evidence and indirect evidence. When p > 0.05, local consistency was considered good; when p < 0.05, the inconsistency model analysis was used.

It is worth noting that all data were retrieved from the intention-to-treat population (ITT) and investigator-reported adverse events. Outcome data were extracted at the end of the randomized controlled phase. When the number of events was 0, 1 was added to the number of events and the total number of participants in the corresponding group. The MCMC consistent model automatically processes multi-arm studies to prevent repeated calculation of the control group.

## 3 Results

### 3.1 Literature screening

A total of 1,142 articles were retrieved, and 275 duplicate articles were deleted. Screening of titles and abstracts resulted in the exclusion of 678 reviews, cohort studies, and animal studies. Of the remaining 157 articles, 63 articles for which the full text could not be obtained were deleted. The 94 articles initially included were read and screened again according to the inclusion and exclusion criteria. Finally, 32 articles were included, involving 15,205 patients. The screening process is shown in **Figure 1**.

**Figure 1:**
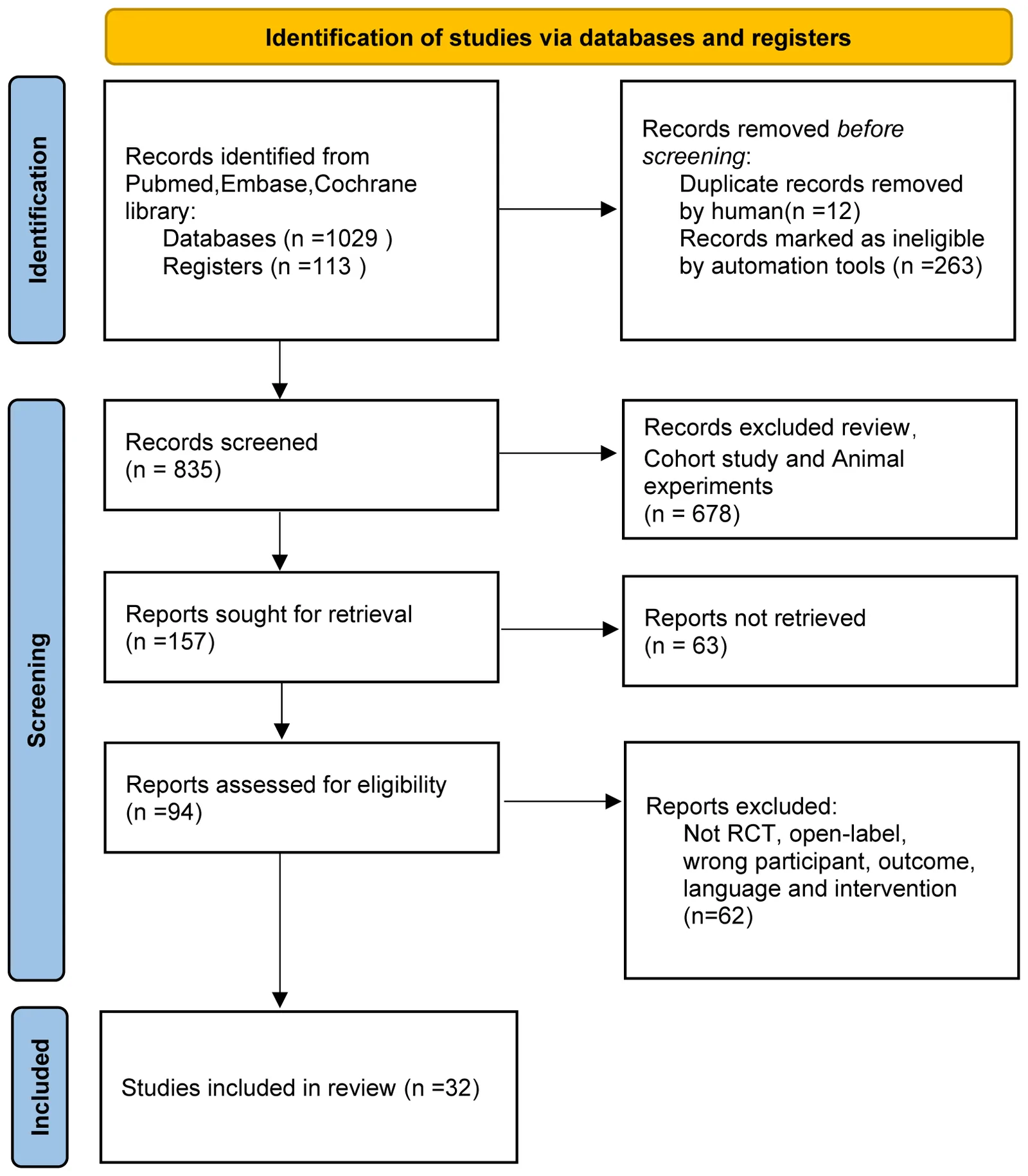
Literature Screening Flowchart.

### 3.2 Characteristics of the included literature

Of the 32 articles included, 16 were TCZ interventions (18–33), 5 articles were ANA (34–38), 5 articles were OKZ (39–43), and 6 articles were SAR (44–49). There were 31 articles reporting AEs, 28 reporting SAEs, 23 reporting infection, 20 reporting serious infection, 22 reporting AEs leading to discontinuation, 21 reporting death, 15 reporting general disorders and administration site conditions, 10 reporting abnormal laboratory investigations, 12 reporting gastrointestinal disorders, 7 reporting neurological disorders, 11 reporting skin and subcutaneous tissues disorders, 9 reporting musculoskeletal and connective tissue disorders, 7 reporting vascular disorders, 6 reporting injury, poisoning, and procedural complications, 9 reporting ALT, 6 reporting AST, 8 reporting nasopharyngitis, 12 reporting upper respiratory tract infection, 3 reporting benign, malignant, and unspecified neoplasms, 5 reporting herpes zoster, and 3 reporting sepsis. The characteristics of the included studies are shown in **Table 1**. The characteristics of intervention measures are shown in **Table 2**. Information on the other included articles is provided in the **Supplementary Material** (**Supplementary Table S3**).

**Table 1:** Characteristics of the included studies.

Study	Diagnostic criteria	Sex (male/female)	Age (years)	Course (years)	DAS28 (mean)	Outcomes
G Jones (2010) (18)	ACR	T: 50/236 C: 60/224	T: 50.7 C: 50.0	T: 6.4 C: 6.2	T: 6.8 C: 6.8	ⓐⓑⓒⓓⓔⓕⓖⓗⓘⓙⓚⓛⓜⓝⓟⓠⓢ
R.N.Maini (2006) (19)	ACR	T_1_: 14/38 T_2_: 11/39 C: 11/38	T_1_: 50.1 T_2_: 50.1 C: 50.9	T_1_: 9.21 T_2_: 10.62 C: 11.24	T_1_: 6.43 T_2_: 6.47 C: 6.75	ⓐⓑⓓⓠ
P Emery (2008) (20)	ACR	T: 28/142 C: 33/125	T: 53.9 C: 53.4	T: 12.6 C: 11.4	T: 6.79 C: 6.80	ⓐⓑⓒⓓⓖⓗⓘⓙⓚⓛⓜⓝⓟⓠ
Mark C. Genovese (2008) (21)	ACR	T: 153/650 C: 66/347	T: 53.0 C: 54.0	T: 9.8 C: 9.8	T: 6.7 C: 6.6	ⓐⓑⓒⓓⓖⓗⓘⓙⓚⓛⓜⓝⓞⓟⓠⓡ
Norihiro Nishimoto (2009) (22)	ACR	T: 6/55 C: 16/48	T: 50.8 C: 52.6	T: 8.7 C: 8.5	T: 6.2 C: 6.1	ⓐⓑⓔⓕⓖ
ALAN KIVITZ (2014) (23)	ACR	T: 62/375 C: 38/181	T: 50.1 C: 52.2	T: 11.1 C: 11.1	T: 6.7 C: 6.6	ⓐⓑⓒⓓⓟⓠ
Tian Liu (2025) (24)	ACR	T_1_: 18/118 T_2_: 13/123 C: 15/53	T_1_: 47.0 T_2_: 47.8 C: 47.8	T_1_: 8.9 T_2_: 8.8 C: 8.0	T_1_: 6.3 T_2_: 6.3 C: 6.0	ⓐⓑⓒⓕⓠ
Johannes W J Bijlsma (2016) (25)	ACR or EULAR	T_1_: 41/65 T_2_: 25/78 C: 39/69	T_1_: 53.0 T_2_: 55.0 C: 53.5	T_1_: 24.5(days) T_2_: 25.5(days) C: 27.0(days)	T_1_: 5.2 T_2_: 5.3 C: 5.1	ⓐⓑⓓⓠⓣ
Yusuf Yazici (2012) (26)	ACR	T: 84/325 C: 33/172	T: 55.2 C: 55.8	T: 8.62 C: 8.52	T: 6.53 C: 6.55	ⓐⓑⓟⓠ
Joel M. Kremer (2018) (27)	ACR	T: 37/110 C: 37/110	T: 56.4 C: 54.6	T: 6.8 C: 6.8	T: 6.3 C: 6.2	ⓐⓑⓓ
Maxime Dougados (2013) (28)	ACR	T: 50/227 C: 59/217	T: 53.0 C: 53.6	T: 8.2 C: 8.3	T: 6.3 C: 6.3	ⓐⓑⓓⓟⓠⓣ
Josef S Smolen (2008) (29)	ACR	T: 30/175 C: 45/159	T: 50.8 C: 50.6	T: 7.5 C: 7.8	T: 6.8 C: 6.8	ⓐⓑⓔⓕⓖⓗⓘⓙⓚⓝⓜ
Han Joo Baek (2019) (30)	ACR	T: 5/42 C: 6/42	T: 52.6 C: 52.0	T: 10.8 C: 8.9	T: 6.1 C: 6.1	ⓐⓑⓒⓓⓖⓗⓝⓟⓠ
J.L. Pablos (2019) (31)	–	T: 20/62 C: 17/65	T: 50.2 C: 51.0	T: 5.8 C: 6.4	T: 5.4 C: 5.3	ⓐⓑⓒⓖⓗⓘⓚⓛⓜⓝ
Kristina Lend (2024) (33)	ACR or EULAR	T: 59/129 C: 64/153	T: 52.4 C: 54.3	T: 8 (days) C: 6 (days)	T: 4.9 C: 5.0	ⓐⓑⓕⓖⓚⓝ
Gerd R Burmester (2016) (32)	ACR	T_1_: 62/228 T_2_: 73/219 C: 58/229	T_1_: 49.5 T_2_: 49.9 C: 49.6	T_1_: 0.5 T_2_: 0.5 C: 0.4	T_1_: 6.7 T_2_: 6.7 C: 6.6	ⓐⓑⓒⓓⓟⓠⓣ
JOHN TESSER (2004) (34)	ACR	–	–	–	–	ⓑⓒⓓⓕⓚ
Roy M. Fleischmann (2003) (35)	ACR	T: 282/834 C: 72/211	T: 54.6 C: 55.7	–	–	ⓐⓑⓒⓓⓚⓟⓠ
S B Cohen (2004) (36)	ACR	T: 52/198 C: 63/188	T: 56.0 C: 57.0	T:11.0 C:10.0	–	ⓐⓑⓚⓠ
Michael H. Schiff (2004) (37)	ACR	T: 93/268 C: 26/61	T: 49.1 C: 51.7	T: 9.3 C: 9.4	–	ⓐⓑⓒⓓⓕⓚⓞⓡ
Stanley Cohen (2002) (38)	ACR	T: 27/45 C: 11/63	T: 54.1 C: 53.0	T: 8.0 C: 7.8	–	ⓐⓚⓠ
Mark C Genovese (2013) (39)	ACR	T_1_: 2/20 T_2_: 5/17 T_3_: 6/37 C: 5/17	T_1_: 52.6 T_2_: 54.5 T_3_: 56.5 C: 58.1	T_1_: 10.8 T_2_: 7.8 T_3_: 10.5 C: 7.5	T_1_: 6.14 T_2_: 5.83 T_3_: 5.73 C: 5.69	ⓐⓑⓒⓓⓖⓚⓟⓢ
Eugen Feist (2022) (40)	ACR or EULAR	T: 31/130 C: 14/55	T: 53.9 C: 53.0	T: 12.7 C: 9.8	T: 6.0 C: 6.2	ⓐⓒⓖⓗⓘⓙⓚⓛⓜⓝⓟ
Tsutomu Takeuchi (2016) (41)	ACR	T_1_: 1/31 T_2_: 11/21 C: 4/25	T_1_: 53.9 T_2_: 56.7 C: 52.6	T_1_: 7.6 T_2_: 6.9 C: 6.5	T_1_: 5.5 T_2_: 5.3 C: 5.3	ⓐⓑⓒⓓⓔⓖⓘⓚⓝⓞⓟⓠⓡ
Josef S. Smolen (2022) (42)	ACR	T: 101/378 C: 53/190	T: 53.7 C: 54.3	T: 7.4 C: 6.9	T: 5.8 C: 5.8	ⓐⓑⓒⓓⓠⓟⓢ
Evgeniy Nasonov (2021) (43)	ACR or EULAR	T: 24/118 C: 23/120	T: 49.1 C: 52.7	T: 7.3 C: 8.4	T: 5.9 C: 6.0	ⓐⓑⓒⓓⓔⓕⓗⓙⓣⓝⓞ
Mark C. Genovese (2020) (44)	ACR	–	–	–	–	ⓐⓑⓒⓓⓟⓠⓣ
Tom W J Huizinga (2013) (45)	ACR	T_1_: 9/42 T_2_: 10/42 C: 14/38	T_1_: 51.2 T_2_: 48.7 C: 55.2	T_1_: 7.74 T_2_: 5.95 C: 8.07	T_1_: 6.11 T_2_: 6.06 C: 6.08	ⓐⓒⓔⓕⓗⓙⓛⓟⓠⓣ
Roy Fleischmann (2017) (46)	ACR	T_1_: 39/142 T_2_: 33/151 C: 27/154	T_1_: 54.0 T_2_: 52.9 C: 51.9	T_1_: 11.6 T_2_: 12.7 C: 12.0	T_1_: 6.1 T_2_: 6.3 C: 6.2	ⓐⓑⓒⓔⓕⓙⓟⓣⓠ
Mark C. Genovese (2015) (47)	ACR	T_1_: 20/80 T_2_: 15/85 C: 19/81	T_1_: 50.1 T_2_: 50.8 C: 50.9	T_1_: 9.5 T_2_: 8.6 C: 9.1	T_1_: 6.0 T_2_: 6.0 C: 5.9	ⓐⓑⓒⓕⓙⓟⓠⓣ
Paul Emery (2019) (48)	ACR	T_1_: 8/41 T_2_: 12/39 C: 20/82	T_1_: 54.8 T_2_: 51.7 C: 50.4	T_1_: 13.6 T_2_: 10.5 C: 10.8	T_1_: 5.8 T_2_: 5.8 C: 5.9	ⓐⓑⓒⓓⓟⓠ
Yoshiya Tanaka (2019) (49)	ACR or EULAR	T_1_: 18/63 T_2_: 19/61 C: 16/65	T_1_: 56.1 T_2_: 55.3 C: 53.5	T_1_: 7.0 T_2_: 8.3 C: 8.2	T_1_: 5.7 T_2_: 5.4 C: 5.4	ⓐⓑⓒⓔⓕⓖⓜⓝⓟⓠ

ⓐAE; ⓑSAE; ⓒinfection; ⓓSerious infection; ⓔNasopharyngitis; ⓕUpper respiratory tract infection; ⓖGastrointestinal disorders; ⓗMusculoskeletal and connective tissue disorders; ⓘNervous system disorders; ⓙAbnormal laboratory investigations; ⓚGeneral disorders and administration site conditions; ⓛInjury, poisoning and procedural complications; ⓜVascular disorders; ⓝSkin and subcutaneous disorders; ⓞPneumonia; ⓟdeath; ⓠAEs leading to discontinuation; ⓡCellulitis; ⓢTumor; ⓣALT. T, Treatment Group; C, Control Group;.

**Table 2:** Characteristics of interventions in the included studies.

Study	Design	Interventions		Treatment course (week)
		Treatment (sample)	Control (sample)	
G Jones (2010) (18)	Two-arm	Tocilizumab 8mg/kg (n=288)	MTX(n=284)	24
R. N. Maini (2006) (19)	Three-arm	T_1_: Tocilizumab 8mg/kg (n=52) T_2_: Tocilizumab 8mg/kg + MTX (n=50)	MTX(n=49)	20
P Emery (2008) (20)	Two-arm	Tocilizumab 8mg/kg + MTX (n=170)	Placebo+ MTX (n=158)	24
Mark C. Genovese (2008) (21)	Two-arm	Tocilizumab 8mg/kg + DMARDs (n=803	Placebo + DMARDs (n=413)	24
Norihiro Nishimoto (2009) (22)	Two-arm	Tocilizumab 8mg/kg (n=61)	MTX(n=64)	24
ALAN KIVITZ (2014) (23)	Two-arm	Tocilizumab-SC 162mg every other week + MTX (n=437)	Placebo + MTX (n=218)	24
Tian Liu (2025) (24)	Three-arm	T_1_: Tocilizumab-SC 162mg every other week + MTX (n=136) T_2_: Tocilizumab-SC 162mg every other week + Placebo (n=136)	Placebo + MTX (n=68)	24
Johannes W J Bijlsma (2016) (25)	Three-arm	T_1_: Tocilizumab 8mg/kg + MTX (n=106) T_2_: Tocilizumab 8mg/kg (n=103)	MTX (n=108)	24
Yusuf Yazici (2012) (26)	Two-arm	Tocilizumab 8mg/kg + DMARDs (n=409)	Placebo + MTX (n=205)	24
Joel M. Kremer (2018) (27)	Two-arm	Tocilizumab-SC 162mg every other week + MTX(n=147)	Tocilizumab-SC 162mg every other week (n=147)	24
Maxime Dougados (2013) (28)	Two-arm	Tocilizumab 8mg/kg + MTX (n=277)	Tocilizumab 8mg/kg (n=276)	24
Josef S Smolen (2008) (29)	Two-arm	Tocilizumab 8mg/kg + MTX (n=206)	Placebo + MTX (n=204)	24
Han Joo Baek (2019) (30)	Two-arm	Tocilizumab 8mg/kg (n=48)	Placebo (n=51)	24
J.L. Pablos (2019) (31)	Two-arm	Tocilizumab 8mg/kg + MTX (n=83)	Placebo + Tocilizumab 8mg/kg (n=82)	28
Gerd R Burmester (2016) (32)	Three-arm	T_1_: Tocilizumab 8mg/kg + MTX (n=290) T_2_: Tocilizumab 8mg/kg (n=292)	Placebo + MTX (n=282)	24
Kristina Lend (2024) (33)	Two-arm	Tocilizumab 8mg/kg + MTX (n=188)	MTX (n=217)	24
JOHN TESSER (2004) (34)	Two-arm	Anakinra-SC 100mg/day (n=346)	Placebo (n=100)	24
Roy M. Fleischmann (2003) (35)	Two-arm	Anakinra-SC 100mg/day (n=1116)	Placebo (n=283)	24
S B Cohen (2004) (36)	Two-arm	Anakinra-SC 100mg/day + MTX (n=250)	Placebo + MTX (n=251)	24
Michael H. Schiff (2004) (37)	Two-arm	Anakinra-SC 100mg/day (n=361)	Placebo (n=87)	24
Stanley Cohen (2002) (38)	Two-arm	Anakinra-SC 2mg/kg/day + MTX(n=72)	Placebo (n=74)	24
Mark C Genovese (2013) (39)	Four-arm	T_1_: olokizumab(60mg) every four week (n=22) T_2_: olokizumab(240mg) every four week (n=22) T_3_: Tocilizumab 8mg/kg (n=43)	Placebo (n=22)	12
Eugen Feist (2022) (40)	Two-arm	olokizumab(64mg) every four week (n=161)	Placebo (n=69)	24
Tsutomu Takeuchi (2016) (41)	Three-arm	T_1_: olokizumab(60mg) every four week (n=32) T_2_: olokizumab(240mg) every four week (n=26)	Placebo (n=29)	12
Josef S. Smolen (2022) (42)	Two-arm	olokizumab(64mg) every four week (n=479)	Placebo (n=243)	24
Evgeniy Nasonov (2021) (43)	Two-arm	olokizumab(64mg) every four week (n=142)	Placebo (n=142)	24
Mark C. Genovese (2020) (44)	Four-arm	T_1_: Sarilumab(150mg) every two week + MTX (n=401) T_2_: Sarilumab(200mg) every two week + MTX (n=396) T_3_: Sarilumab(200mg) every two week (n=184)	Placebo (n=397)	52(T_1_ and T_2_) 24(T_3_)
Tom W J Huizinga (2013) (45)	Three-arm	T_1_: Sarilumab(150mg) every two week (n=51) T_2_: Sarilumab(200mg) every two week (n=52)	Placebo (n=52)	22
Roy Fleischmann (2017) (46)	Three-arm	T_1_: Sarilumab(150mg) every two week + DMARDs (n=181) T_2_: Sarilumab(200mg) every two week + DMRADs (n=184)	Placebo +DMARDs (n=52)	24
Mark C. Genovese (2015) (47)	Three-arm	T_1_: Sarilumab(150mg) every two week + MTX(n=400) T_2_: Sarilumab(200mg) every two week + MTX(n=399)	Placebo +MTX (n=398)	52
Paul Emery (2019) (48)	Three-arm	T_1_: Sarilumab(150mg) every two week + DMARDs (n=49) T_2_: Sarilumab(200mg) every two week + DMARDs (n=51)	Tocilizumab 8mg/kg + DMARDs (n=102)	24
Yoshiya Tanaka (2019) (49)	Three-arm	T_1_: Sarilumab(150mg) every two week + MTX (n=81) T_2_: Sarilumab(200mg) every two week + MTX(n=80)	Placebo +MTX (n=81)	24

T, Treatment Group; C, Control Group; MTX, Methotrexate; DMARDs, disease-modifying antirheumatic drugs.

### 3.3 Risk of bias in outcomes

For the randomization process, the majority of articles were rated as low risk because of the use of interactive voice response systems (IVRS) and random stratification. The articles rated as having some concerns only mentioned random grouping and did not specify the method. For deviations from established interventions, one study was rated as high risk because medications could have been changed during treatment (25). Five study were rated as having some concerns because the obvious injection-site reactions associated with ANA may have compromised blinding (34–38). The remaining studies were rated as low risk because they used double-blinding and intention-to-treat (ITT) analysis. For missing outcome data, three articles were rated as high risk because of a significant difference in completion rates between the experimental group and the control group or a dropout rate greater than 20% (22, 38, 47). The remaining studies were rated as low risk because loss to follow-up was limited and balanced between groups. For measurement of the outcome, the majority of studies were rated as low risk because all trials were double-blind and generally used two independent blinded readers. For Selection of the reported result, six articles were rated as having some concerns because they did not report the pre-registration number (19, 34–38). For the overall risk-of-bias assessment, all evaluations were rated as low risk when they were rated as low risk. Evaluations were rated as having some concerns when at least one domain raised some concerns but there was no high risk, and as high risk when at least one domain was rated as high risk. In addition, funding sponsors of several studies participated in the experimental design, data collection, and article writing process (24, 42, 45, 47). These studies were rated as having some concerns overall. The risk-of-bias results are shown in **Figure 2**. The comparison-correction funnel plot was used to assist in assessing small-study effects and publication bias. The results showed that the funnel plot was not completely symmetrical, suggesting the presence of small-study effects or mild publication bias. The comparison-correction funnel plot is shown in **Figure 3**.

**Figure 2:**
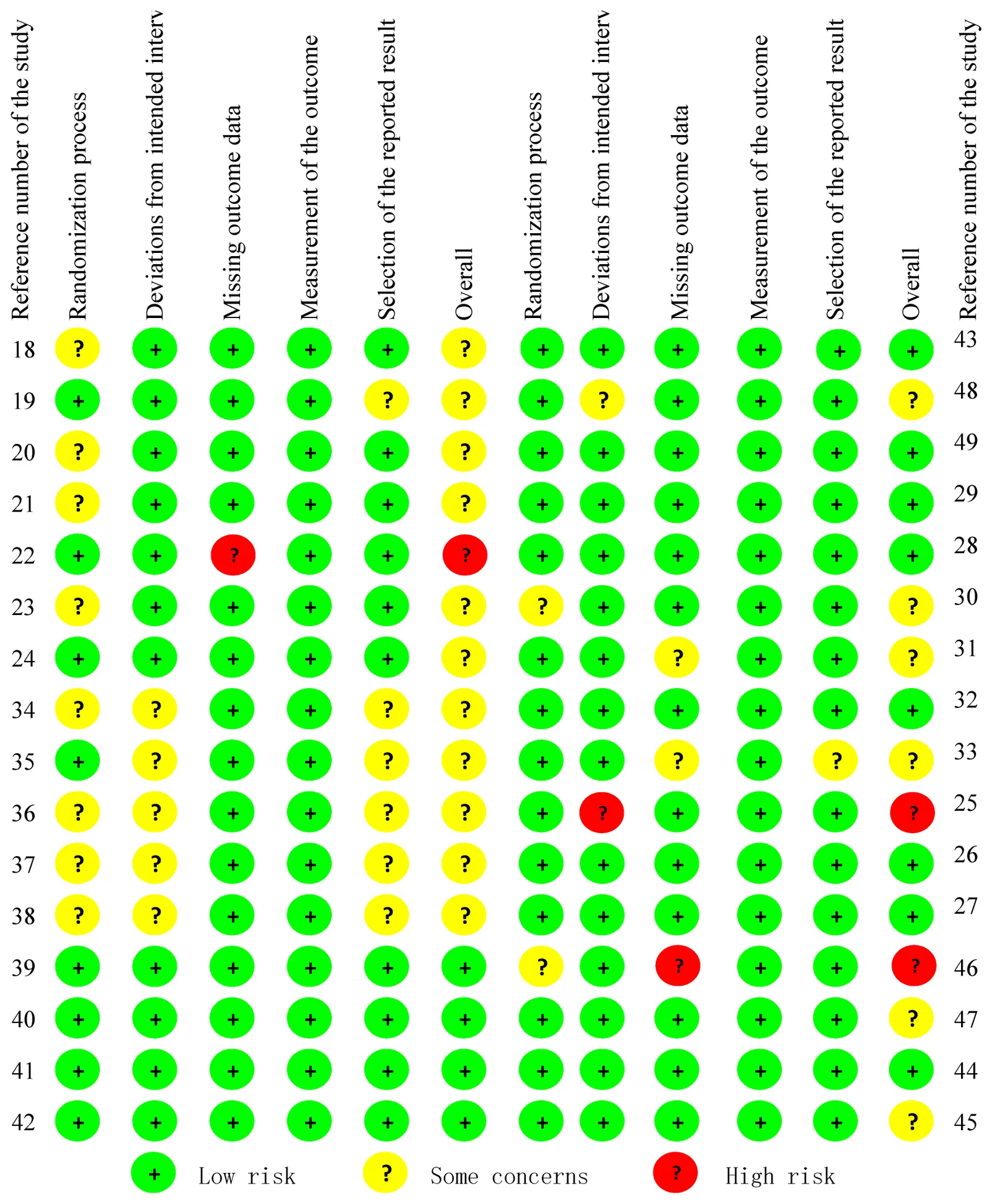
Results of the risk of bias evaluation.

**Figure 3:**
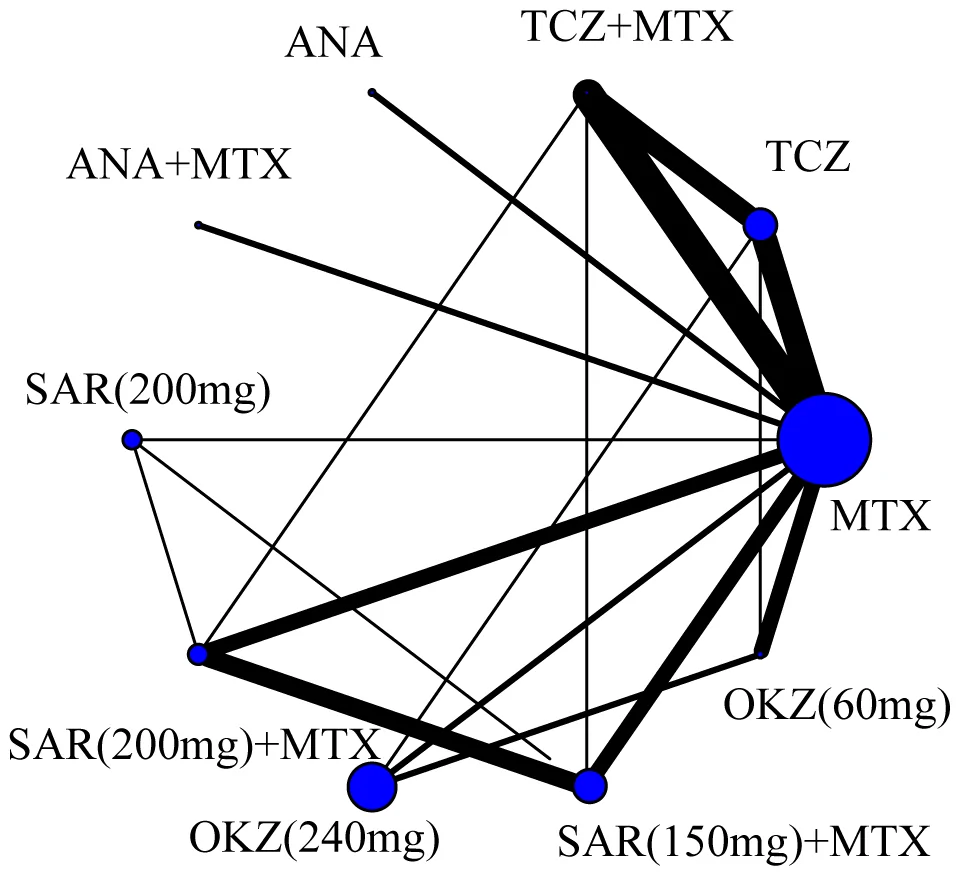
The network structure diagram of AE. MTX, Methotrexate; TCZ, Tocilizumab; ANA, Anakinra; SAR, Sarilumab; OKZ, Olokizumab.

### 3.4 Directly compared meta-analysis results

#### 3.4.1 Overall safety outcomes

There was a statistical difference between the use of interleukin inhibitors and the overall occurrence of AEs (OR = 1.15, 95% CI [1.09, 1.21], p < 0.0001). The incidence of AEs was higher with TCZ + MTX (OR = 1.16, 95% CI [1.05, 1.29], p = 0.004), SAR (150 mg) + MTX (OR = 1.23, 95% CI [1.07, 1.40], p = 0.002), and SAR (200 mg) + MTX (OR = 1.26, 95% CI [1.10, 1.43], p < 0.0001) than with MTX. (**Supplementary Table S4**)

There was a statistically significant difference in the overall incidence of SAEs (OR = 1.22, 95% CI [1.08, 1.38], p = 0.001). The incidence of SAEs with SAR (150 mg) + MTX (OR = 1.50, 95% CI [1.05, 2.13], p = 0.023) and SAR (200 mg) + MTX (OR = 1.91, 95% CI [1.36, 2.68], p < 0.0001) was significantly higher than with MTX. (**Supplementary Table S4**)

There was a statistically significant difference in the overall incidence of AEs leading to a discontinuation with interleukin inhibitor use (OR = 1.65, 95% CI [1,32, 2.06], p < 0.0001). The incidence in the TCZ (OR = 1.52, 95% CI [1.03, 2.23], p = 0.034), TCZ + MTX (OR = 2.02, 95% CI [1.45, 2.81], p < 0.0001), SAR (150 mg) + MTX (OR = 2.29, 95% CI [1.65, 3.18], p < 0.0001), and SAR (200 mg) + MTX (OR = 2.55, 95% CI [1.85, 3.53], p < 0.0001) treatment groups was significantly higher than with MTX. However, the incidence of MTX AEs leading to discontinuation was significantly higher when ANA was used (OR = 0.37, 95% CI [0.22, 0.62], p < 0.0001). (**Supplementary Table S4**)

There were statistical differences in the use of interleukin inhibitors and general disorders and administration site conditions (OR = 1.85, 95% CI [1.49, 2.29], p < 0.0001). The incidence in the OKZ (60 mg) (OR = 2.27, 95% CI [1.14, 4.51], p = 0.019), ANA (OR = 2.19, 95% CI [1.80, 2.66], p < 0.0001), ANA + MTX (OR = 2.76, 95% CI [1.97, 3.86], p < 0.0001), and SAR (200 mg) + MTX (OR = 4.05, 95% CI [1.10, 14.89], p = 0.035) treatment groups was significantly higher than with MTX. (**Supplementary Table S4**)

#### 3.4.2 Infection-related outcomes

There was a statistically significant difference in the overall incidence of infection (OR = 1.10, 95% CI [1.02, 1.18], p = 0.007). The incidence in the SAR (150 mg) + MTX (OR = 1.25, 95% CI [1.06, 1.48], p = 0.007), TCZ + MTX (OR = 1.20, 95% CI [1.00, 1.45], p = 0.049), and SAR (200 mg) + MTX (OR = 1.24, 95% CI [1.05, 1.46], p = 0.014) treatment groups was significantly higher than with MTX. There was no statistical difference in the incidence of nasopharyngitis (OR = 1.24, 95% CI [0.96, 1.58], p = 0.089) and upper respiratory tract infections (OR = 1.23, 95% CI [0.92, 1.63], p = 0.151) associated with interleukin inhibitor use. (**Supplementary Table S4**)

There was a statistical difference in the overall occurrence of serious infections associated with interleukin inhibitor use (OR = 1.35, 95% CI [1.00, 1.83], p = 0.046). There was also a statistical difference in the incidence of cellulitis (OR = 2.33, 95% CI [1.01, 5.39], p = 0.047). There was no statistical difference in the incidence of pneumonia (OR = 0.84, 95% CI [0.56, 1.26], p = 0.409), herpes zoster (OR = 1.53, 95% CI [0.52, 4.47], p = 0.318), and sepsis (OR = 1.70, 95% CI [0.42, 6.93], p = 0.454) in serious infections when using interleukin inhibitors. (**Supplementary Table S4**)

#### 3.4.3 Laboratory outcomes

There was a statistical difference between the use of interleukin inhibitors and abnormal laboratory investigations (OR = 1.99, 95% CI [1.59, 2.48], p < 0.0001). The incidence in the SAR (150 mg) + MTX (OR = 1.85, 95% CI [1.30, 2.68], p = 0.001), SAR (200 mg) + MTX (OR = 2.10, 95% CI [1.48, 2.97], p < 0.0001), TCZ + MTX (OR = 2.71, 95% CI [1.45, 5.09], p = 0.002), and OKZ (60 mg) (OR = 2.01, 95% CI [1.25, 3.24], p = 0.004) treatment groups was significantly higher than with MTX. There was a statistical difference between the use of interleukin inhibitors and increased ALT in laboratory investigations (OR = 1.88, 95% CI [1.36, 2.60], p < 0.0001). The incidence in the SAR (150 mg) + MTX (OR = 2.76, 95% CI [1.80, 4.24], p < 0.0001), SAR (200 mg) + MTX (OR = 2.64, 95% CI [1.68, 4.15], p < 0.0001), SAR (200 mg) (OR = 2.87, 95% CI [1.19, 6.94], p = 0.019), TCZ + MTX (OR = 1.36, 95% CI [1.05, 1.76], p = 0.017), and OKZ (60 mg) (OR = 3.00, 95% CI [1.45, 6.16], p = 0.003) treatment groups was higher than with MTX. There was no statistical difference in the overall incidence of increased AST in laboratory investigations associated with interleukin inhibitor use (OR = 1.24, 95% CI [0.91, 1.68], p = 0.158). (**Supplementary Table S4**)

#### 3.4.4 Organ-system adverse events

The use of interleukin inhibitors resulted in statistical differences in gastrointestinal AEs (OR = 1.38, 95% CI [1.04, 1.83], p = 0.002), and the incidence in the TCZ + MTX (OR = 1.42, 95%CI [1.17, 1.72], p<0.0001) treatment group was higher than that in the MTX group. The use of interleukin inhibitors resulted in statistical differences in neurological AEs (OR = 1.32, 95% CI [1.03, 1.68], p = 0.024), and the incidence in the TCZ treatment group was higher than that in the MTX group (OR = 2.02, 95% CI [1.12, 3.64], p = 0.018). There was a statistically significant difference in skin and subcutaneous tissue AEs (OR = 1.86, 95% CI [1.52, 2.28], p < 0.0001), and the occurrence in the TCZ + MTX treatment group was higher than that in the MTX group (OR = 2.16, 95% CI [1.68, 2.78], p < 0.0001). There was a statistically significant difference between the use of interleukin inhibitors and vascular AEs (OR = 1.45, 95% CI [1.04, 2.02], p = 0.026). It is worth noting that interleukin inhibitor use was associated with a statistically significant reduction in musculoskeletal and connective tissue AEs (OR = 0.77, 95% CI [0.62, 0.95], p = 0.014), and the incidence in the TCZ + MTX treatment group was lower than that in the MTX group (OR = 0.73, 95% CI [0.57, 0.93], p = 0.014). (**Supplementary Table S4**)

#### 3.4.5 Rare serious outcomes

There was no statistical difference in the incidence of death (OR = 0.82, 95% CI [0.52, 1.30], p = 0.414), injury, poisoning, and procedural complications (OR = 1.07, 95%,CI [0.79, 1.47], p = 0.632), or benign, malignant, and unspecified neoplasms (OR = 0.53, 95% CI [0.23, 1.22], p = 0.137) associated with interleukin inhibitor use. (**Supplementary Table S4**)

### 3.5 Results of network meta-analysis

#### 3.5.1 AEs

The AE network included 10 therapeutic measures, forming 12 closed loops (**Figure 3**). A total of 14,759 patients were included, with 10,870 cases. The convergence of the model showed that PSRF was equal to 1, and the density map shows that the multi-chain posterior density curves of each parameter are highly coincident, with smooth curves and no multi-peak or irregular shapes, indicating that the MCMC has good convergence (**Supplementary Figure S1–S2**). Therefore, MCMC was used to fit the consistency model analysis. Pairwise comparison analysis showed that the incidence of AEs after MTX treatment was significantly lower than with TCZ, TCZ + MTX, ANA + MTX, SAR (200 mg) + MTX, SAR (150 mg) + MTX, and OKZ (60 mg). The incidence of AEs with SAR (200 mg) + MTX was significantly higher than with TCZ, TCZ + MTX, and ANA. The incidence of AEs after ANA treatment was significantly lower than that with ANA + MTX, TCZ + MTX, and SAR (150 mg) + MTX. (**Table 3**). The probability ranking showed ANA > MTX > OKZ (60 mg) > TCZ > TCZ + MTX > SAR (150 mg) + MTX > SAR (200 mg) + MTX > ANA + MTX. SUCRA comprehensive ranking showed that ANA (0.89) was superior to other interleukin inhibitors (**Table 4**). The node-splitting method showed that no significant inconsistency was found in each comparison group (**Supplementary Figure S3**).

**Table 3:** NMA results for AEs and SAEs.

MTX	1.28 (0.94, 1.8)	**1.37 (1.06, 1.8)**	0.96 (0.66, 1.4)	1.28(0.46,3.63)	0.85(0.37,1.78)	**1.91(1.28,2.74)**	1.11(0.21,4.28)	1.43(0.94,2.09)	1.01(0.54,1.89)
**0.7 (0.58, 0.85)**	TCZ	1.08(0.78,1.46)	0.75(0.45,1.22)	1.01(0.34,3)	0.67(0.27,1.49)	1.49(0.88,2.37)	0.87(0.16,3.37)	1.12(0.64,1.8)	0.79(0.39,1.56)
**0.64(0.56,0.74)**	0.92(0.76,1.1)	TCZ + MTX	0.7 (0.44, 1.1)	0.94(0.32,2.75)	0.62(0.26,1.35)	1.39(0.86,2.13)	0.81(0.15,3.16)	1.04(0.63,1.61)	0.73(0.37,1.45)
1.05(0.69,1.65)	1.5(0.94,2.45)	**1.63(1.04,2.62)**	ANA	1.34(0.45,4.05)	0.89(0.35,2.02)	**1.99(1.13,3.33)**	1.16(0.21,4.71)	1.49(0.83,2.53)	1.06(0.51,2.19)
**0.42(0.25,0.68)**	0.6(0.34,1)	0.65(0.38,1.08)	**0.4(0.2,0.75)**	ANA + MTX	0.66(0.17,2.4)	1.48(0.49,4.38)	0.86(0.12,4.81)	1.11(0.36,3.28)	0.79(0.24,2.62)
0.72(0.49,1.05)	1.02(0.67,1.57)	1.12(0.74,1.67)	0.68(0.38,1.2)	1.72(0.92,3.24)	SAR (200mg)	**2.23(1.08,5.02)**	1.3(0.21,6.53)	1.67(0.8,3.76)	1.18(0.45,3.41)
**0.49(0.4,0.6)**	**0.7(0.53,0.92)**	**0.76(0.61,0.96)**	**0.47(0.29,0.74)**	1.18(0.69,2.04)	0.68(0.47,1.01)	SAR (200mg) + MTX	0.58(0.11,2.36)	0.75(0.52,1.06)	0.53(0.26,1.1)
0.87(0.34,2.01)	1.24(0.48,2.89)	1.35(0.52,3.11)	0.82(0.3,2.1)	2.08(0.71,5.6)	1.2(0.44,3)	1.77(0.68,4.15)	OKZ (240mg)	1.28(0.31,6.89)	0.92(0.23,4.86)
**0.5(0.45,0.65)**	0.77(0.59,1.01)	0.84(0.67,1.06)	**0.51(0.32,0.82)**	1.3(0.76,2.25)	0.75(0.52,1.11)	1.1(0.9,1.35)	0.62(0.26,1.62)	SAR (150mg) + MTX	0.71(0.34,1.51)
**0.7(0.55,0.91)**	1(0.73,1.38)	1.09(0.82,1.47)	0.67(0.4,1.1)	1.69(0.97,2.98)	0.98(0.62,1.56)	1.43(1.04,1.98)	0.81(0.35,2.1)	1.3(0.95,1.8)	OKZ (60mg)

The left half represents AEs, and the right half represents SAEs. Statistically significant differences are shown in bold. MTX, Methotrexate; TCZ, Tocilizumab; ANA, Anakinra; SAR, Sarilumab; OKZ, Olokizumab.

**Table 4:** Probability ranking results for AEs.

Drugs (SUCRA)	RANK1	RANK2	RANK3	RANK4	RANK5	RANK6	RANK7	RANK8	RANK9	RANK10
MTX (0.88)	0.24	**0.51**	**0.23**	0.01	0.00	0.00	0.00	0.00	0.00	0.00
TCZ (0.56)	0.00	0.01	0.11	0.26	**0.31**	0.21	0.08	0.02	0.00	0.00
TCZ+MTX (0.43)	0.00	0.00	0.01	0.06	0.19	**0.35**	**0.33**	0.05	0.01	0.00
ANA (0.89)	**0.45**	0.29	0.17	0.05	0.02	0.01	0.01	0.00	0.00	0.00
ANA+MTX (0.07)	0.00	0.00	0.00	0.01	0.01	0.02	0.05	0.09	0.14	**0.67**
SAR(200mg) (0.57)	0.02	0.05	0.18	0.24	0.16	0.13	0.14	0.05	0.02	0.01
SAR(200mg)+MTX (0.11)	0.00	0.00	0.00	0.00	0.00	0.01	0.03	0.18	**0.56**	0.21
OKZ(240mg) (0.68)	0.29	0.13	0.17	0.09	0.06	0.05	0.06	0.04	0.05	0.07
SAR(150mg)+MTX (0.22)	0.00	0.00	0.00	0.00	0.01	0.04	0.18	**0.52**	0.20	0.04
OKZ(60mg) (0.55)	0.00	0.01	0.12	**0.27**	0.24	0.18	0.13	0.04	0.01	0.00

The bold font represents the probability ranking of the therapies. MTX, Methotrexate; TCZ, Tocilizumab; ANA, Anakinra; SAR, Sarilumab; OKZ, Olokizumab.

#### 3.5.2 SAEs

The SAE network included 10 therapeutic measures, forming 12 closed loops (**Figure 4**). A total of 14,342 patients were included, with 1,927 cases. The convergence of the model showed that PSRF was equal to 1, and the density map shows that the multi-chain posterior density curves of each parameter are highly coincident, with smooth curves and no multi-peak or irregular shapes, indicating that the MCMC has good convergence (**Supplementary Figure S4–S5**). Pairwise comparison analysis showed that the incidence of SAEs after TCZ + MTX treatment was significantly higher than with MTX. The incidence of SAEs after SAR (200 mg) + MTX treatment was significantly higher than with SAR (200 mg), ANA, and MTX (**Table 3**). The probability ranking showed that SAR (200 mg) >ANA > MTX > TCZ > TCZ + MTX > SAR (150mg) + MTX > SAR (200 mg) + MTX. SUCRA comprehensive ranking showed that SAR (200 mg) (0.76) was superior to other interleukin inhibitors (**Table 5**). The node-splitting method showed that no significant inconsistency was found in each comparison group (**Supplementary Figure S6**).

**Figure 4:**
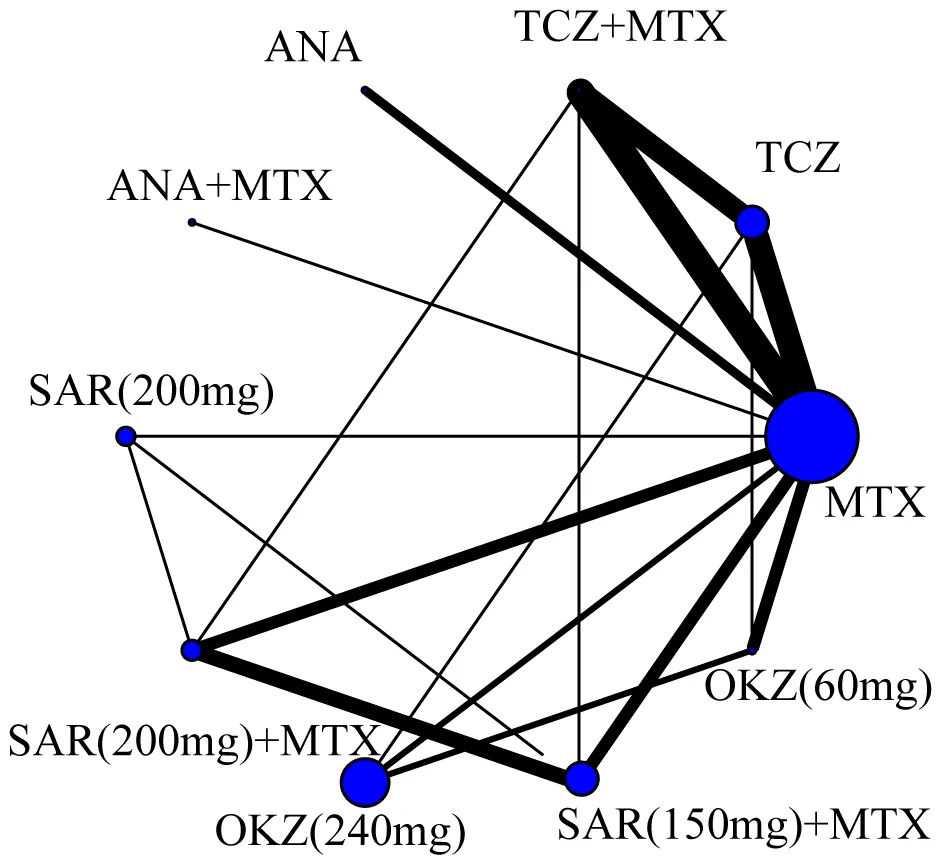
The network structure diagram of SAEs. MTX, Methotrexate; TCZ, Tocilizumab; ANA, Anakinra; SAR, Sarilumab; OKZ, Olokizumab.

**Table 5:** Probability ranking results for SAEs.

Drugs (SUCRA)	RANK1	RANK2	RANK3	RANK4	RANK5	RANK6	RANK7	RANK8	RANK9	RANK10
MTX (0.70)	0.03	0.14	**0.30**	**0.30**	**0.17**	0.05	0.01	0.00	0.00	0.00
TCZ (0.42)	0.00	0.02	0.04	0.09	0.16	**0.24**	0.22	0.14	0.07	0.02
TCZ+MTX (0.33)	0.00	0.00	0.01	0.03	0.10	0.20	**0.27**	0.24	0.12	0.02
ANA (0.72)	0.12	**0.23**	0.22	0.18	0.12	0.07	0.03	0.02	0.01	0.00
ANA+MTX (0.44)	0.12	0.11	0.07	0.07	0.08	0.08	0.07	0.09	0.12	0.20
SAR(200mg) (0.76)	**0.34**	0.20	0.12	0.09	0.08	0.06	0.05	0.04	0.02	0.01
SAR(200mg)+MTX (0.08)	0.00	0.00	0.00	0.00	0.01	0.02	0.04	0.10	**0.32**	**0.52**
OKZ(240mg) (0.54)	0.27	0.11	0.06	0.05	0.06	0.05	0.05	0.06	0.09	0.20
SAR(150mg)+MTX (0.32)	0.00	0.01	0.02	0.05	0.10	0.15	0.19	**0.26**	0.21	0.02
OKZ(60mg) (0.64)	0.13	0.19	0.15	0.13	0.12	0.09	0.07	0.06	0.04	0.02

The bold font represents the probability ranking of the therapies. MTX, Methotrexate; TCZ, Tocilizumab; ANA, Anakinra; SAR, Sarilumab; OKZ, Olokizumab.

#### 3.5.3 Infection

Infection included 9 treatments that formed 12 closed loops (**Figure 5**). A total of 13,468 patients were included, with 4,905 cases. The convergence of the model showed that PSRF was equal to 1, and the density map shows that the multi-chain posterior density curves of each parameter are highly coincident, with smooth curves and no multi-peak or irregular shapes, indicating that the MCMC has good convergence (**Supplementary Figure S7-S8**). Pairwise comparison analysis showed that the incidence of infection after MTX treatment was significantly lower than with TCZ + MTX and SAR (150 mg) + MTX (**Table 6**). The probability ranking showed SAR (200 mg) > OKZ (60 mg) > MTX > TCZ + MTX > TCZ > OKZ (60 mg) > ANA. SUCRA comprehensive ranking showed that SAR (200 mg) (0.93) was superior to other interleukin inhibitors (**Table 7**). The node-splitting method showed that no significant inconsistency was found in each comparison group (**Supplementary Figure S9**).

**Figure 5:**
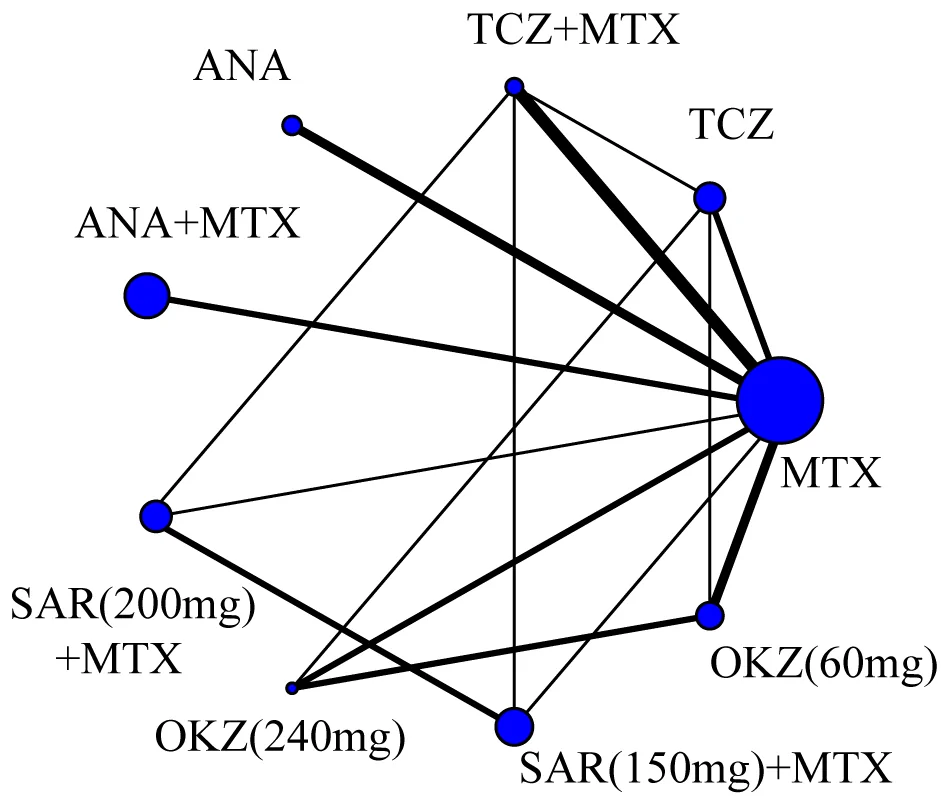
The network structure diagram of infection. MTX, Methotrexate; TCZ, Tocilizumab; ANA, Anakinra; SAR, Sarilumab; OKZ, Olokizumab.

**Table 6:** NMA results for infections and serious infections.

MTX	1.56(0.79,3.24)	1.53(0.9,2.82)	**3.46(1.1,15.95)**	0.37(0.05,1.73)	1.6(0.65,4.13)	2.3(0.56,8.88)	1.09(0.41,2.92)	0.88(0.32,2.42)
0.85(0.61,1.17)	TCZ	0.98(0.5,2)	2.23(0.56,11.69)	0.23(0.03,1.25)	1.03(0.34,3.08)	1.46(0.35,5.86)	0.7(0.22,2.21)	0.56(0.17,1.78)
**0.73(0.57,0.94)**	0.86(0.61,1.23)	TCZ + MTX	2.25(0.61,11.47)	0.24(0.03,1.19)	1.04(0.38,2.82)	1.49(0.35,6.18)	0.71(0.24,2.01)	0.57(0.18,1.79)
1.13(0.75,1.67)	1.32(0.8,2.23)	1.54(0.96,2.47)	ANA	**0.1(0.01,0.74)**	0.46(0.08,2.04)	0.64(0.08,4.11)	0.31(0.05,1.45)	0.25(0.04,1.22)
1.07(0.58,2)	1.26(0.63,2.56)	1.47(0.76,2.86)	0.95(0.46,1.99)	SAR (200mg)	4.34(0.99,35.74)	6.45(0.75,73.75)	2.93(0.62,24.66)	2.41(0.38,25)
0.76(0.55,1.07)	0.9(0.58,1.44)	1.05(0.71,1.56)	0.68(0.41,1.15)	0.71(0.38,1.33)	SAR (200mg) + MTX	1.42(0.27,7.17)	0.68(0.26,1.68)	0.55(0.14,2.11)
0.79(0.36,1.77)	0.93(0.41,2.15)	1.08(0.47,2.5)	0.7(0.29,1.72)	0.74(0.27,2.03)	1.03(0.44,2.46)	OKZ (240mg)	0.47(0.09,2.64)	0.38(0.09,1.7)
**0.69(0.49,0.94)**	0.8(0.51,1.27)	0.94(0.63,1.38)	0.61(0.36,1.01)	0.64(0.34,1.18)	0.9(0.64,1.23)	0.87(0.36,2.04)	SAR (150mg) + MTX	0.81(0.2,3.27)
1.11(0.75,1.67)	1.31(0.8,2.19)	1.52(0.96,2.46)	0.99(0.56,1.75)	1.04(0.5,2.18)	1.46(0.87,2.44)	1.41(0.62,3.2)	1.62(0.97,2.75)	OKZ (60mg)

The left half represents infection and the right half represents serious infection. Statistically significant differences are shown in bold. MTX, Methotrexate; TCZ, Tocilizumab; ANA, Anakinra; SAR, Sarilumab; OKZ, Olokizumab.

**Table 7:** Probability ranking results for infections.

Drugs (SUCRA)	RANK1	RANK2	RANK3	RANK4	RANK5	RANK6	RANK7	RANK8	RANK9
MTX (0.70)	0.03	0.2	0.36	**0.27**	0.11	0.03	0.01	0	0
TCZ (0.39)	0	0.03	0.06	0.11	0.18	0.22	**0.24**	0.13	0.03
TCZ+MTX (0.39)	0	0.02	0.04	0.12	**0.21**	**0.26**	0.22	0.11	0.02
ANA (0.10)	0	0.01	0.01	0.02	0.03	0.05	0.07	0.21	**0.59**
SAR(200mg) (0.93)	**0.76**	0.11	0.05	0.03	0.02	0.02	0.01	0.01	0
SAR(200mg)+MTX (0.37)	0	0.04	0.08	0.11	0.16	0.17	0.21	0.18	0.07
OKZ(240mg) (0. 25)	0.02	0.04	0.05	0.05	0.07	0.09	0.11	0.3	0.27
SAR(150mg)+MTX (0.61)	0.04	0.23	0.19	0.17	0.13	0.11	0.08	0.04	0.01
OKZ(60mg) (0.71)	0.14	0.34	0.16	0.12	0.09	0.07	0.05	0.03	0

The bold font represents the probability ranking of the therapies. MTX, Methotrexate; TCZ, Tocilizumab; ANA, Anakinra; SAR, Sarilumab; OKZ, Olokizumab.

#### 3.5.4 Serious infection

Serious Infection included 9 treatment measures, forming 12 closed loops (**Figure 6**). A total of 9,679 patients were included, with 225 cases. The convergence of the model showed that PSRF was equal to 1, and the density map shows that the multi-chain posterior density curves of each parameter are highly coincident, with smooth curves and no multi-peak or irregular shapes, indicating that the MCMC has good convergence (**Supplementary Figure S10-S11**). Pairwise comparison analysis showed that the incidence of serious infection after ANA treatment was significantly higher than with MTX (**Table 6**). Probabilistic ranking showed that SAR (200 mg) > OKZ (60 mg) > MTX > TCZ + MTC > TCZ > ANA. SUCRA comprehensive ranking showed that SAR (200 mg) (0.93) was superior to other interleukin inhibitors (**Table 8**). The node-splitting method showed that no significant inconsistency was found in each comparison group (**Supplementary Figure S12**).

**Figure 6:**
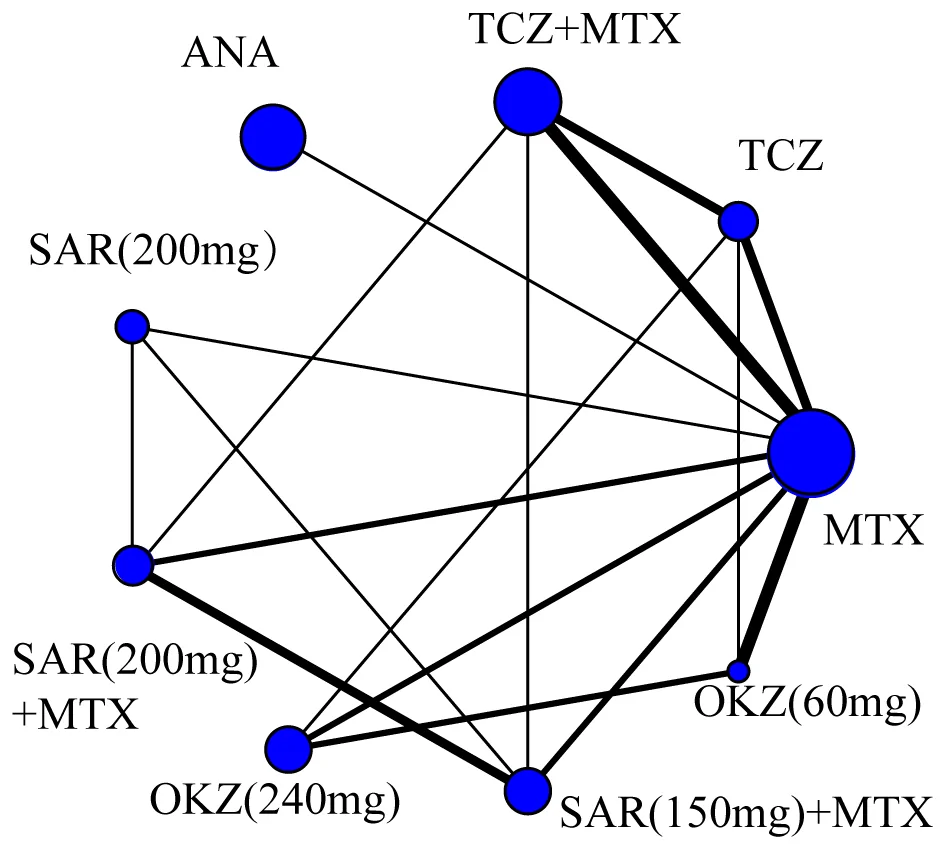
The network structure diagram of serious infection. MTX, Methotrexate; TCZ, Tocilizumab; ANA, Anakinra; SAR, Sarilumab; OKZ, Olokizumab.

**Table 8:** Probability ranking results for serious infections.

Drugs (SUCRA)	RANK1	RANK2	RANK3	RANK4	RANK5	RANK6	RANK7	RANK8	RANK9
MTX (0.70)	0.03	0.2	**0.36**	**0.27**	0.11	0.03	0.01	0	0
TCZ (0.39)	0	0.03	0.06	0.11	0.18	0.22	**0.24**	0.13	0.03
TCZ+MTX (0.39)	0	0.02	0.04	0.12	**0.21**	**0.26**	0.22	0.11	0.02
ANA (0.10)	0	0.01	0.01	0.02	0.03	0.05	0.07	**0.21**	**0.59**
SAR(200mg) (0.93)	**0.76**	0.11	0.05	0.03	0.02	0.02	0.01	0.01	0
SAR(200mg)+MTX (0.37)	0	0.04	0.08	0.11	0.16	0.17	0.21	0.18	0.07
OKZ(240mg) (0.25)	0.02	0.04	0.05	0.05	0.07	0.09	0.11	0.3	0.27
SAR(150mg)+MTX (0.61)	0.04	0.23	0.19	0.17	0.13	0.11	0.08	0.04	0.01
OKZ(60mg) (0.71)	0.14	**0.34**	0.16	0.12	0.09	0.07	0.05	0.03	0

The bold font represents the probability ranking of the therapies. MTX, Methotrexate; TCZ, Tocilizumab; ANA, Anakinra; SAR, Sarilumab; OKZ, Olokizumab.

#### 3.5.5 AEs leading to discontinuation

AEs leading to discontinuation included 10 kinds of treatment measures, forming 9 closed loops (**Figure 7**). A total of 12,038 patients were included, with 871 cases. The convergence of the model showed that PSRF was equal to 1.01, and the density map shows that the multi-chain posterior density curves of each parameter are highly coincident, with smooth curves and no multi-peak or irregular shapes, indicating that the MCMC has good convergence (**Supplementary Figures S13, S14**). Pairwise comparison analysis showed that AEs leading to discontinuation after MTX treatment was significantly lower than with TCZ + MTX, SAR (200 mg) + MTX, and SAR (150 mg) + MTX, but higher than with ANA. The incidence with TCZ and TCZ+MTX was significantly higher than with ANA. The incidence with ANA, ANA + MTX, and SAR (200 mg) was significantly lower than with SAR (200 mg) + MTX. The incidence with ANA, ANA + MTX, and SAR (200 mg) was also lower than with SAR (150 mg) + MTX. In addition, the incidence with ANA was lower than with ANA + MTX, SAR (200 mg), and OKZ (60 mg) (**Table 9**). The probability ranking showed that ANA > MTX > ANA + MTX > TCZ > TCZ + MTX > SAR (150 mg) + MTX > SAR (200 mg) + MTX. SUCRA comprehensive ranking showed that ANA (0.98) was superior to other interleukin inhibitors (**Table 10**). The node-splitting method showed that no significant inconsistency was found in each comparison group (**Supplementary Figure S15**).

**Figure 7:**
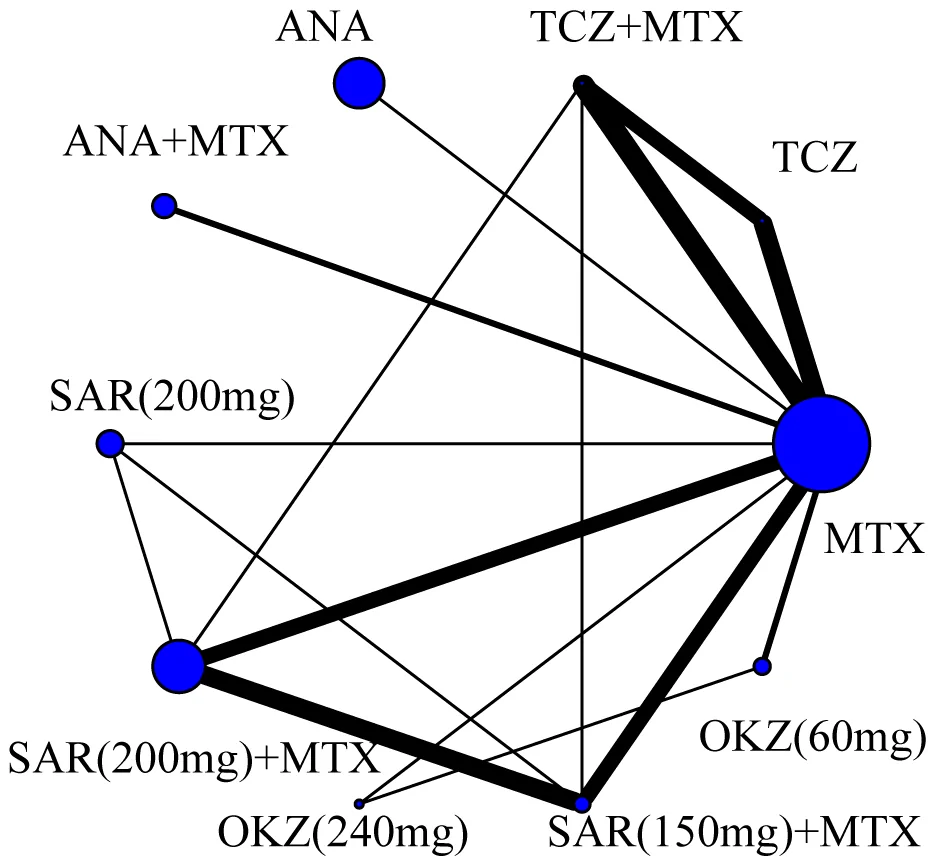
The network structure diagram of AEs leading to discontinuation. MTX, Methotrexate; TCZ, Tocilizumab; ANA, Anakinra; SAR, Sarilumab; OKZ, Olokizumab.

**Table 9:** NMA results for AEs leading to discontinuation and death.

MTX	0.64 (0.21, 1.82)	0.9 (0.36, 2.19)	1.32 (0.14, 28.65)	NA	0.81 (0.03, 7.13)	0.64 (0.2, 2)	0.97 (0.11, 6.04)	0.75 (0.24, 2.25)	0.87 (0.23, 3.37)
0.69 (0.48, 1.01)	TCZ	1.4 (0.44, 4.76)	2.12 (0.17, 53.19)	NA	1.25 (0.04, 14.88)	0.99 (0.21, 4.84)	1.55 (0.16, 11.24)	1.19 (0.26, 5.49)	1.4 (0.27, 7.45)
**0.48 (0.35, 0.68)**	0.7 (0.49, 1.01)	TCZ + MTX	1.47(0.13,34.82)	NA	0.89 (0.03, 9.12)	0.71 (0.18, 2.74)	1.07 (0.11, 8.33)	0.84 (0.22, 3.08)	0.98 (0.2, 5)
**2.87 (1.54, 5.23)**	**4.13 (2, 8.46)**	**5.93 (2.87, 11.72)**	ANA	NA	0.55 (0.01, 14.53)	0.48 (0.02, 5.93)	0.69 (0.02, 14.04)	0.56 (0.02, 7.02)	0.64 (0.02, 9.6)
0.82 (0.5, 1.35)	1.19 (0.63, 2.23)	1.71 (0.92, 3.07)	**0.29 (0.13, 0.63)**	ANA + MTX	NA	NA	NA	NA	NA
0.72 (0.37, 1.52)	1.05 (0.48, 2.38)	1.5 (0.71, 3.3)	**0.25 (0.1, 0.65)**	0.88 (0.38, 2.13)	SAR (200mg)	0.81(0.08,21.13)	1.27 (0.05, 49.08)	0.93 (0.1, 25.05)	1.14 (0.08, 35.95)
**0.32 (0.22, 0.46)**	**0.46 (0.28, 0.77)**	0.66 (0.41, 1.05)	**0.11 (0.06, 0.23)**	**0.39 (0.21, 0.72)**	**0.44 (0.22, 0.83)**	SAR (200mg) + MTX	1.53 (0.14, 13.41)	1.18 (0.35, 4.04)	1.36 (0.25, 8.12)
0.7 (0.1, 6.32)	1.01 (0.14, 9.3)	1.45 (0.2, 13.64)	0.25 (0.03, 2.39)	0.85 (0.11, 8)	0.96 (0.12, 9.48)	2.18 (0.31, 20.93)	OKZ (240mg)	0.78 (0.09, 8.23)	0.9 (0.13, 9)
**0.37 (0.26, 0.53)**	**0.53 (0.32, 0.89)**	0.76 (0.47, 1.21)	**0.13 (0.06, 0.26)**	**0.45 (0.24, 0.84)**	**0.51 (0.25, 0.98)**	1.15 (0.86, 1.57)	0.53 (0.06, 3.77)	SAR (150mg) + MTX	1.17(0.21, 6.65)
0.61 (0.28, 1.3)	0.88 (0.37, 2.06)	1.27 (0.54, 2.9)	**0.21 (0.08, 0.57)**	0.74 (0.3, 1.85)	0.84 (0.29, 2.3)	1.92 (0.81, 4.37)	0.88 (0.1, 5.81)	1.66 (0.7, 3.77)	OKZ (60mg)

The left half represents AEs leading to discontinuation, and the right half represents death. Statistically significant differences are shown in bold. NA, Not Applicable. MTX, Methotrexate; TCZ, Tocilizumab; ANA, Anakinra; SAR, Sarilumab; OKZ, Olokizumab.

**Table 10:** Probability ranking results for AEs leading to discontinuation.

Drugs (SUCRA)	RANK1	RANK2	RANK3	RANK4	RANK5	RANK6	RANK7	RANK8	RANK9	RANK10
MTX (0.79)	0.00	**0.37**	**0.42**	0.18	0.04	0.00	0.00	0.00	0.00	0.00
TCZ (0.53)	0.00	0.01	0.08	0.20	**0.31**	**0.25**	0.13	0.01	0.00	0.00
TCZ+MTX (0.29)	0.00	0.00	0.00	0.01	0.04	0.17	**0.32**	**0.37**	0.07	0.02
ANA (0.98)	**0.89**	0.11	0.00	0.00	0.00	0.00	0.00	0.00	0.00	0.00
ANA+MTX(0.64)	0.00	0.12	0.23	**0.25**	0.20	0.12	0.05	0.02	0.00	0.00
SAR(200mg) (0.56)	0.00	0.11	0.13	0.18	0.19	0.17	0.13	0.07	0.01	0.00
SAR(200mg)+MTX (0.05)	0.00	0.00	0.00	0.00	0.00	0.00	0.02	0.07	0.30	**0.60**
OKZ(240mg) (0.51)	0.11	0.23	0.06	0.06	0.07	0.07	0.07	0.07	0.05	0.21
SAR(150mg)+MTX (0.15)	0.00	0.00	0.00	0.00	0.00	0.02	0.08	0.27	**0.51**	0.12
OKZ(60mg) (0.45)	0.00	0.04	0.08	0.12	0.16	0.19	0.19	0.12	0.05	0.04

The bold font represents the probability ranking of the therapies. MTX, Methotrexate; TCZ, Tocilizumab; ANA, Anakinra; SAR, Sarilumab; OKZ, Olokizumab.

#### 3.5.6 Death

Death included 9 therapeutic measures and formed 12 closed loops (**Figure 8**). A total of 11,853 patients were included, with 76 cases. The convergence of the model showed that PSRF was equal to 1.01, and the density map shows that the multi-chain posterior density curves of each parameter are highly coincident, with smooth curves and no multi-peak or irregular shapes, indicating that the MCMC has good convergence (**Supplementary Figures S16, S17**). Pairwise comparison analysis showed no statistical difference between any two treatment measures. Due to the uncertainty, the probabilistic ranking and SUCRA comprehensive ranking were used only to explore the trend analysis (**Table 9**). The probability ranking showed that SAR (200 mg) > SAR (200 mg) + MTX > TCZ > SAR (150 mg) + MTX > OKZ (240 mg) > ANA. The SUCRA comprehensive ranking showed that SAR (200 mg) + MTX (0.64) tended to outperform other interventions (**Table 11**). The node-splitting method showed that no significant inconsistency was found in each comparison group (**Supplementary Figure S18**).

**Figure 8:**
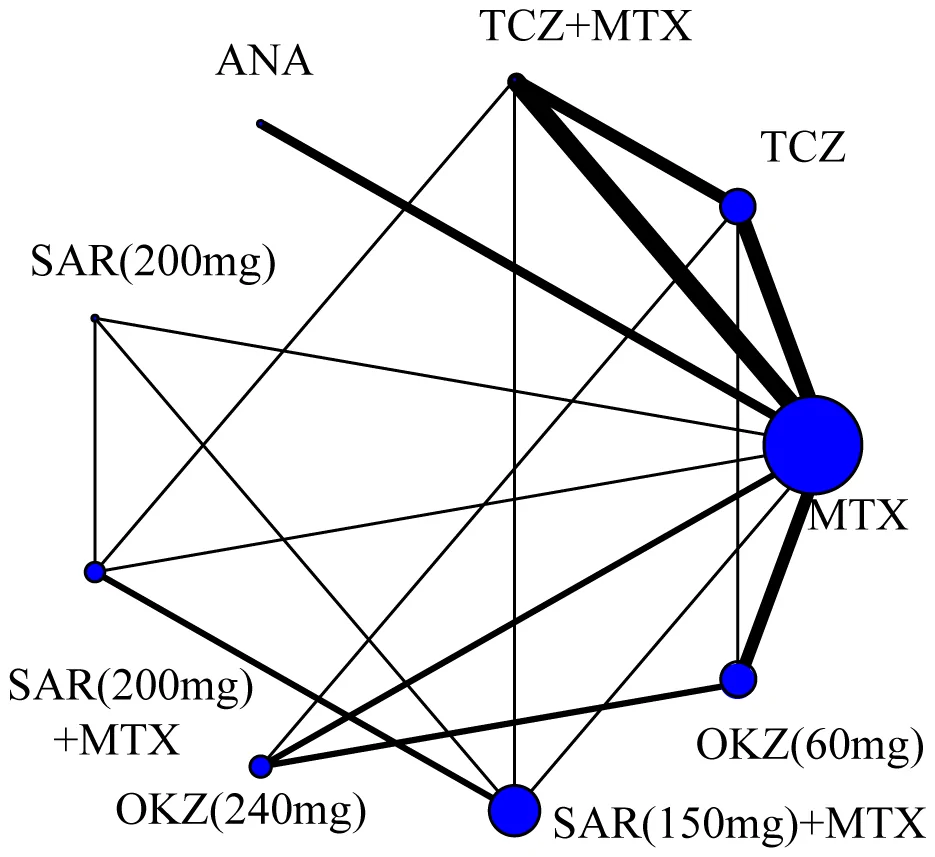
The network structure diagram of Death. MTX, Methotrexate; TCZ, Tocilizumab; ANA, Anakinra; SAR, Sarilumab; OKZ, Olokizumab.

**Table 11:** Probability ranking results for death.

Drugs (SUCRA)	RANK1	RANK2	RANK3	RANK4	RANK5	RANK6	RANK7	RANK8	RANK9
MTX (0.38)	0.00	0.01	0.05	0.12	**0.19**	**0.25**	**0.22**	0.13	0.03
TCZ (0.64)	0.15	0.19	**0.18**	0.14	0.11	0.09	0.07	0.05	0.02
TCZ+MTX (0.45)	0.03	0.07	0.11	0.14	0.16	0.16	0.14	0.12	0.06
ANA (0.35)	0.12	0.08	0.06	0.06	0.05	0.06	0.07	0.12	**0.37**
SAR(200mg) (0.52)	**0.26**	0.10	0.07	0.06	0.05	0.06	0.08	0.13	0.19
SAR(200mg)+MTX (0.64)	0.14	**0.20**	0.17	0.14	0.11	0.09	0.08	0.05	0.02
OKZ(240mg) (0.44)	0.14	0.10	0.08	0.08	0.07	0.08	0.10	**0.16**	0.19
SAR(150mg)+MTX (0.55)	0.08	0.14	0.16	**0.15**	0.13	0.12	0.11	0.08	0.04
OKZ(60mg) (0.48)	0.08	0.11	0.12	0.11	0.11	0.11	0.13	0.14	0.09

The bold font represents the probability ranking of the therapies. MTX, Methotrexate; TCZ, Tocilizumab; ANA, Anakinra; SAR, Sarilumab; OKZ, Olokizumab.

#### 3.5.7 General disorders and administration site conditions

General disorders and administration site conditions included 9 treatment measures, forming 9 closed loops (**Figure 9**). A total of 6,840 patients were included, with 2,104 cases. The convergence of the model showed that PSRF was equal to 1.01, and the density map shows that the multi-chain posterior density curves of each parameter are highly coincident, with smooth curves and no multi-peak or irregular shapes, indicating that the MCMC has good convergence (**Supplementary Figures S19, S20**). Pairwise comparison analysis showed that the incidence with MTX was lower than with ANA, ANA + MTX, SAR (200 mg) + MTX, OKZ (240 mg), SAR (150 mg) + MTX, and OKZ (60 mg). The incidence with TCZ was also lower than with ANA, ANA + MTX, SAR (200 mg) + MTX, OKZ (240 mg), and SAR (150 mg) + MTX. The incidence with TCZ was also lower than with ANA, ANA + MTX, SAR (200 mg) + MTX, and SAR (150 mg) + MTX (**Table 12**). The probability ranking showed that MTX > TCZ + MTX > OKZ (60 mg) > OKZ (240 mg) > ANA > ANA + MTX. The probability ranking and SUCRA values of TCZ (0.87) were superior to other interleukin inhibitors (**Table 13**). The node-splitting method showed that no significant inconsistency was found in each comparison group (**Supplementary Figure S21**).

**Figure 9:**
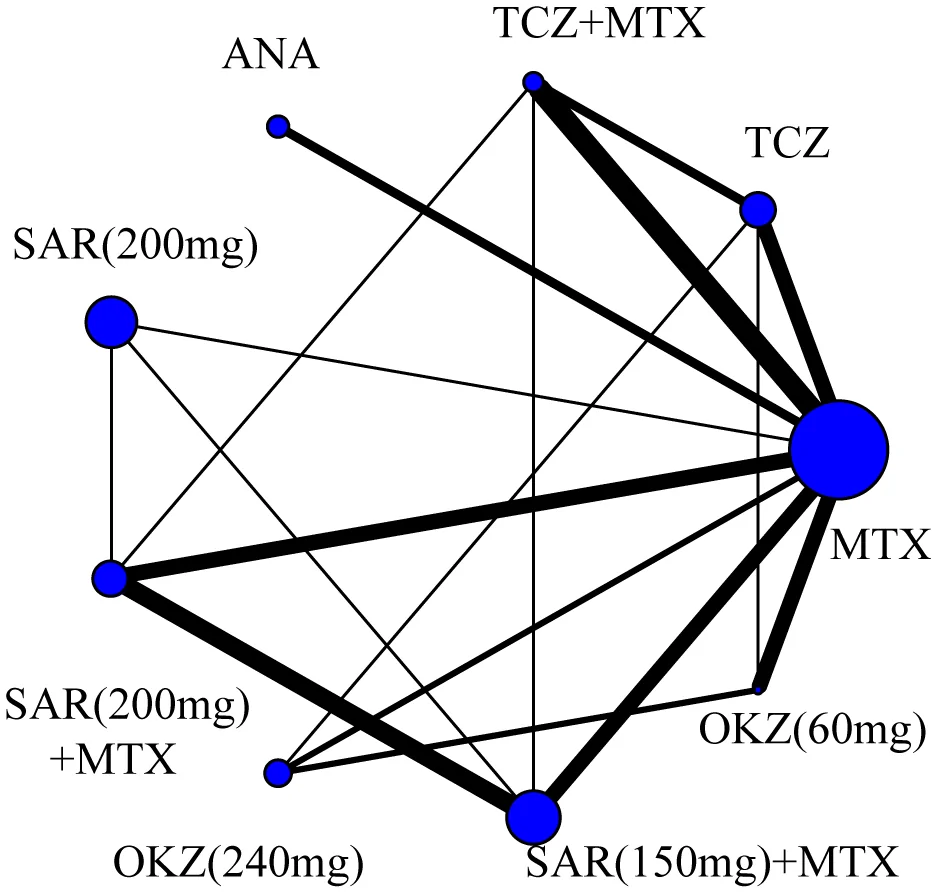
The network structure diagram of General disorders and administration site conditions. MTX, Methotrexate; TCZ, Tocilizumab; ANA, Anakinra; SAR, Sarilumab; OKZ, Olokizumab.

**Table 12:** NMA results for general disorders and administration site conditions and abnormal laboratory investigations.

MTX	1.12 (0.51, 2.47)	**3.18 (1.77, 5.46)**	NA	NA	**2.39 (1.45, 4.01)**	NA	**2.01 (1.2, 3.38)**	**2.52 (1.28, 5.11)**
0.92 (0.48, 1.7)	TCZ	**2.84 (1.04, 7.13)**	NA	NA	2.13 (0.84, 5.44)	NA	1.79 (0.7, 4.59)	2.25 (0.8, 6.43)
0.79 (0.54, 1.18)	0.85 (0.44, 1.78)	TCZ+MTX	NA	NA	0.75 (0.36, 1.66)	NA	0.63 (0.3, 1.4)	0.79 (0.34, 2.02)
**0.18 (0.12, 0.28)**	**0.2 (0.1, 0.43)**	**0.23 (0.13, 0.41)**	ANA	NA	NA	NA	NA	NA
**0.17 (0.09, 0.34)**	**0.19 (0.08, 0.48)**	**0.22 (0.1, 0.47)**	0.94 (0.45, 2.07)	ANA+MTX	NA	NA	NA	NA
**0.15 (0.04, 0.49)**	**0.17 (0.04, 0.62)**	**0.19 (0.05, 0.62)**	0.84 (0.2, 2.84)	0.88 (0.2, 3.26)	SAR(200mg)+MTX	NA	0.84 (0.52, 1.34)	1.05 (0.45, 2.52)
**0.34 (0.13, 0.86)**	**0.37 (0.14, 0.99)**	0.43 (0.16, 1.16)	1.86 (0.66, 5.08)	1.97 (0.62, 5.93)	2.24(0.49,11.28)	OKZ(240mg)	NA	NA
**0.16 (0.04, 0.52)**	**0.18 (0.04, 0.66)**	**0.21 (0.05, 0.67)**	0.9 (0.22, 3.04)	0.95 (0.21, 3.51)	1.07(0.44,2.61)	0.48 (0.1, 2.15)	SAR(150mg)+MTX	1.26 (0.53, 3.02)
**0.39 (0.17, 0.84)**	0.42 (0.17, 1.02)	0.49 (0.19, 1.16)	2.13 (0.83, 5.09)	2.24 (0.77, 6.03)	2.57(0.59,11.71)	1.13 (0.45, 2.83)	2.39(0.55,11.01)	OKZ(60mg)

The left half represents general disorders and administration site conditions, and the right half represents abnormal laboratory investigations. Statistically significant differences are shown in bold. NA, Not Applicable. MTX, Methotrexate; TCZ, Tocilizumab; ANA, Anakinra; SAR, Sarilumab; OKZ, Olokizumab.

**Table 13:** Probability ranking results for general disorders and administration site conditions.

Drugs (SUCRA)	RANK1	RANK2	RANK3	RANK4	RANK5	RANK6	RANK7
MTX (0.93)	**0.54**	**0.40**	0.06	0.00	0.00	0.00	0.00
TCZ (0.87)	0.38	0.30	0.28	0.03	0.01	0.00	0.00
TCZ+MTX (0.79)	0.07	0.28	**0.57**	0.07	0.01	0.00	0.00
ANA (0.23)	0.00	0.00	0.00	0.01	0.05	**0.28**	**0.29**
ANA+MTX (0.21)	0.00	0.00	0.00	0.02	0.05	0.21	0.27
SAR(200mg)+MTX(0.18)	0.00	0.00	0.00	0.04	0.07	0.14	0.16
OKZ(240mg) (0.49)	0.01	0.01	0.03	0.29	**0.40**	0.12	0.08
SAR(150mg)+MTX(0.22)	0.00	0.00	0.00	0.06	0.09	0.17	0.16
OKZ(60mg) (0.54)	0.00	0.01	0.04	**0.47**	0.32	0.08	0.04

The bold font represents the probability ranking of the therapies. MTX, Methotrexate; TCZ, Tocilizumab; ANA, Anakinra; SAR, Sarilumab; OKZ, Olokizumab.

#### 3.5.8 Abnormal laboratory investigations

Abnormal laboratory investigations included 6 treatment measures, forming one closed loop (**Figure 10**). A total of 5,268 patients were included, with 627 cases. The convergence of the model shows that PSRF is equal to 1, and the density map shows that the multi-chain posterior density curves of each parameter are highly coincident, with smooth curves and no multi-peak or irregular shapes, indicating that the MCMC has good convergence (**Supplementary Figures S22, S23**). Pairwise comparison analysis showed that the abnormal laboratory investigation after TCZ + MTX treatment was significantly higher than with MTX and TCZ + MTX. SAR (200 mg) + MTX, SAR (150 mg) + MTX, and OKZ (60 mg) were also associated with significantly higher incidence than MTX. (**Table 12**). Probability ranking showed MTX > TCZ + MTX > OKZ (60 mg) > OKZ (240 mg) > ANA. SUCRA comprehensive ranking showed that TCZ (0.87) may be superior to other treatment measures (**Table 14**). The direct and indirect comparisons in the closed loop are from the same study, and the inconsistency test could not be performed using the node-splitting method. No significant inconsistency was found using the global inconsistency test (p = 0.670).

**Figure 10:**
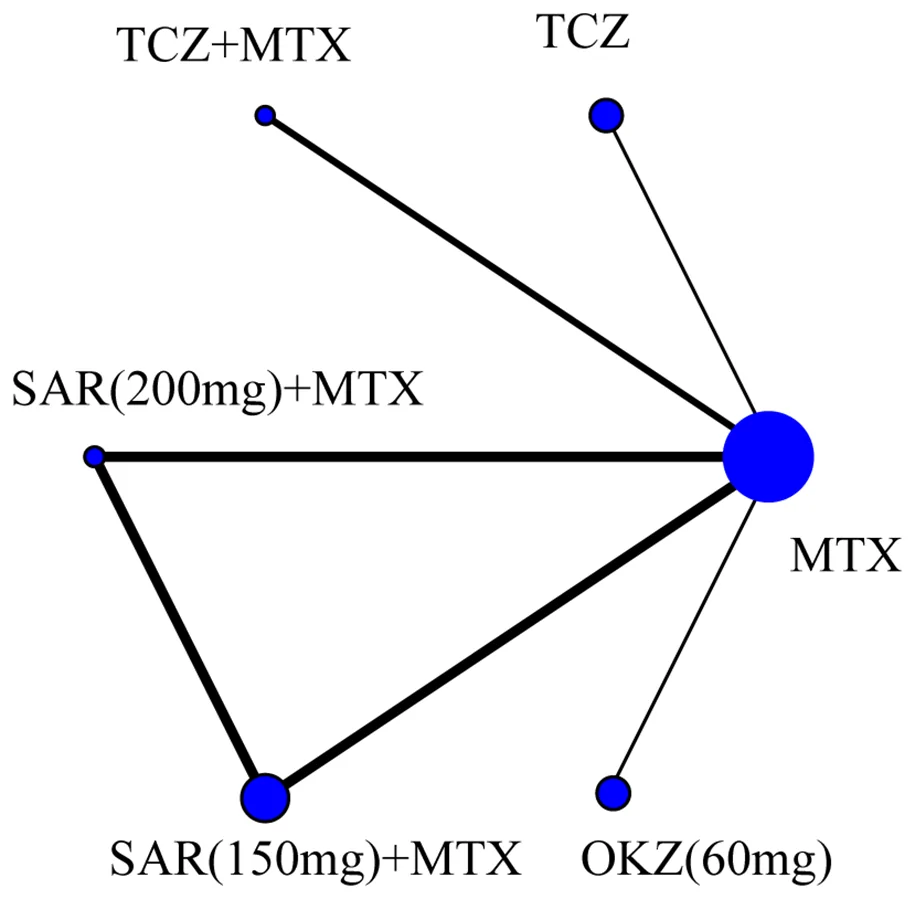
The network structure diagram of Abnormal laboratory investigations. MTX, Methotrexate; TCZ, Tocilizumab; ANA, Anakinra; SAR, Sarilumab; OKZ, Olokizumab.

**Table 14:** Probability ranking results for abnormal laboratory investigations.

Drugs (SUCRA)	RANK1	RANK2	RANK3	RANK4	RANK5	RANK6
MTX (0.92)	**0.63**	0.36	0.01	0.00	0.00	0.00
TCZ (0.83)	0.36	**0.52**	0.07	0.03	0.02	0.01
TCZ+MTX (0.12)	0.00	0.01	0.06	0.10	0.24	**0.60**
SAR(200mg)+MTX (0.31)	0.00	0.01	0.13	**0.40**	**0.32**	0.13
SAR(150mg)+MTX (0.50)	0.00	0.06	**0.52**	0.28	0.10	0.03
OKZ(60mg) (0.29)	0.00	0.03	0.22	0.19	0.31	0.24

The bold font represents the probability ranking of the therapies. MTX, Methotrexate; TCZ, Tocilizumab; ANA, Anakinra; SAR, Sarilumab; OKZ, Olokizumab.

#### 3.5.9 Publication bias, heterogeneity test, and sensitivity analysis

The comparison-correction funnel plot was not completely symmetrical, suggesting mild publication bias and small-study effects (**Figure 11**). The reason for the large heterogeneity in the ANA + MTX treatment group for AEs may be the different doses of the two studies (36, 38). After excluding one study, the overall heterogeneity for AEs leading to discontinuation decreased (I^2^ = 0.0%, p = 0.790) (35).The reason is likely to be that the included patients used different DMARDs at baseline. The heterogeneity for general disorders and administration site conditions decreased after the removal of the TCZ + MTX group (I^2^ = 17.3%, p = 0.260) (20, 21, 29, 33). Differences in patients’ histories of drug treatment failure may have contributed to the heterogeneity of results. For gastrointestinal AEs, patients in the TCZ treatment group were resistant to MTX and TNF at baseline. After excluding one study, the heterogeneity in the TCZ treatment group for gastrointestinal AEs decreased (I^2^ = 0.0%, p = 0.479) (18). After excluding two studies, the heterogeneity for increased ALT in laboratory investigations decreased (I^2^ = 0.0%, p = 0.851) (25, 32). We performed a sensitivity analysis of the main outcomes. After sequentially excluding each individual study, neither the direction nor the statistical significance of the combined effect size changed substantially, suggesting that the results were robust (**Supplementary Figures S24, S31**).

**Figure 11:**
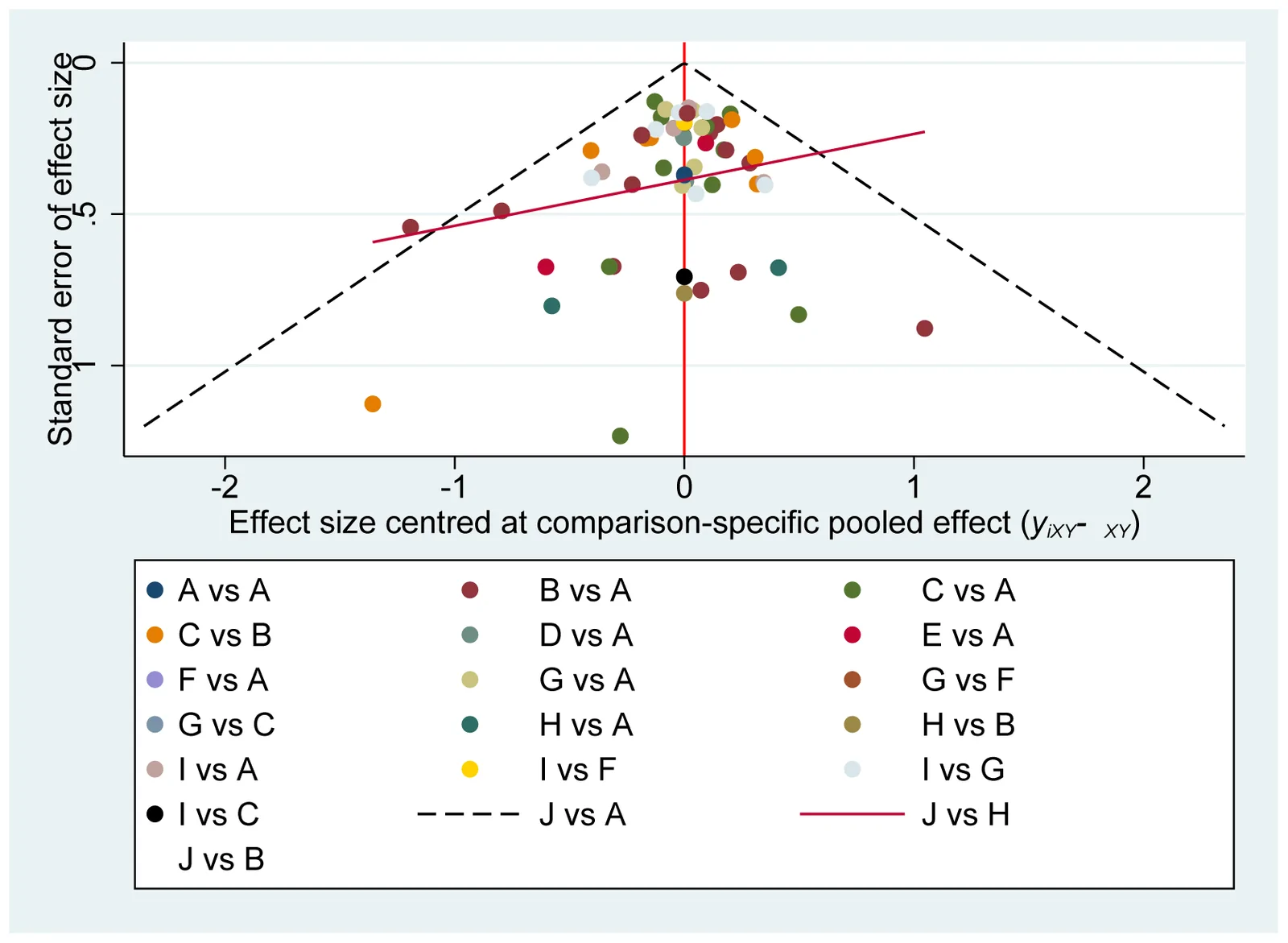
Comparison-correction funnel plot. A,MTX; B, TCZ; C, TCZ+MTX; D, ANA; E, ANA+MTX; F, SAR(200mg); G, SAR(200mg)+MTX; H, OKZ(240mg); I, SAR(150mg)+MTX; J, OKZ(60mg).

## 4 Discussion

In this network meta-analysis, the risk of bias in the included articles was generally low, although some concerns were identified. Heterogeneity and inconsistency were not significant, and the main outcome GRADE score was moderate and low quality under conservative estimation (**Table 15**). We analyzed whether there were differences in the performance of four interleukin inhibitors, comprising a total of 10 interventions, across a variety of adverse events, including AEs, SAEs, infections, serious infections, AEs leading to discontinuation, death, general disorders and administration site conditions, and abnormal laboratory investigations. We performed a direct comparative analysis of all outcomes and a network analysis of the outcomes of at least five studies. When analyzing the data, there were three articles that evaluated OKZ at a dose of 64 mg. Considering the dose-response relationship is not much different, we unified OKZ (60 mg) for analysis (40, 42, 43). Although the control group included in the analysis received either MTX monotherapy or placebo + MTX, both were unified and categorized as MTX. The majority of analyses showed low heterogeneity, and sensitivity analysis indicated that the conclusions were stable. It is worth noting that there was a statistically significant difference in the combined effect size in the direct comparison of results for serious infections, whereas no statistically significant differences were observed in the subgroup analysis. This may be due to the reduction in the number of studies when the original data were divided into smaller subsets, resulting in insufficient statistical power. In addition, there was no statistical difference in the subgroup analysis and pairwise comparison of death, which may be because many articles reported that the death of patients was due to complications. Therefore, whether there was a difference in drug-related mortality among treatments still needs further study. We observed that the incidence of AEs leading to discontinuation with ANA was lower than with MTX in both the direct comparison and network analysis. This may be because only one study reported the incidence of this outcome with ANA (35). Due to the large number of samples in this earlier study and the wide range of population included, slight methodological heterogeneity may have been over-amplified or even reversed. It is worth noting that the abnormal laboratory investigation network contained few treatment nodes and limited direct evidence. This weakens confidence in ranking-based conclusions. In the direct comparisons, sarilumab-containing regimens appeared to be associated with higher incidences of overall AEs, SAEs, infections, AEs leading to discontinuation, and ALT elevation in several comparisons. For the results of the network comparison, the conclusion should not be based solely on the probability ranking and SUCRA to determine which is better, but should also consider the specific clinical context, cost, and convenience.

**Table 15:** Results of GRADE.

Result	Risk of bias	Inconsistency	Indirectness	Imprecision	Publication bias	OR (95%CI)	No. Intervention	No. Control	Final Rating
AE	Serious	Not serious	Not serious	Not serious	Not serious	1.15(1.09, 1.21)	7728/10101 (76.5%)	3142/4658 (67.5%)	Moderate
SAEs	Serious	Not serious	Not serious	Not serious	Not serious	1.22(1.08, 1.38)	1487/9996 (14.9%)	442/4348 (10.2%)	Moderate
Infection	Serious	Not serious	Not serious	Not serious	Not serious	1.10(1.02, 1.18)	3446/9176 (37.6%)	1459/4292 (34.0%)	Moderate
Serious infection	Serious	Not serious	Not serious	Serious	Not serious	1.35(1.00, 1.83)	175/6886 (2.5%)	50/2793 (1.8%)	Low
AEs leading to discontinuation	Serious	Serious	Not serious	Not serious	Not serious	1.65(1.32, 2.06)	667/8341 (8.0%)	204/3697 (5.5%)	Low
Death	Serious	Not serious	Not serious	Serious	Not serious	0.82(0.52, 1.30)	50/8283 (0.6%)	26/3570 (0.7%)	Low
General disorders and administration site conditions	Serious	Serious	Not serious	Not serious	Not serious	1.85(1.49, 2.29)	1754/4565 (38.4%)	350/2275 (15.4%)	Low
Abnormal laboratory investigations	Serious	Not serious	Not serious	Not serious	Not serious	1.99(1.59, 2.48)	470/3255 (14.4%)	157/2013 (7.8%)	Moderate

OR, Odds Ratio; CI, Confidence Interval.

Previous studies have shown that there is no difference in the risk of cardiovascular disease among patients with RA treated with interleukin inhibitors compared with those treated with TNF inhibitors or JAK inhibitors (50). Unlike TNF-α inhibitors, a Mendelian randomization study found a causal relationship between reduced IL-6 signaling and a reduced risk of tuberculosis (14, 51). In contrast to our conclusion, a meta-analysis found that ANA was associated with a significantly higher incidence of serious infections than placebo in the treatment of RA (52). This may be due to the different inclusion criteria. In order to ensure clinical and methodological homogeneity, we excluded long-term follow-up results after the end of the treatment phase (53). Consistent with our findings, a meta-analysis reported that ANA did not increase the risk of malignant tumors (54). In addition, studies have shown that the use of TCZ to inhibit the IL-6 pathway affects metabolism and increases body weight and body mass index (BMI) (55). A Japanese study reported that TCZ may cause mild elevations in blood lipid levels or abnormal liver function in patients with RA but does not lead to serious cardiovascular events (56, 57). It has been reported that although TCZ has lower hepatotoxicity than ANA, it can also cause liver damage (58). Long-term follow-up of patients receiving SAR also found elevated blood lipid levels, elevated ALT, and tumorigenesis (59). It is worth noting that long-term follow-up of patients receiving OKZ identified positive results in the examination for Mycobacterium tuberculosis (60).

The use of IL-6 inhibitors may temporarily reduce the neutrophil count but does not appear to affect neutrophil functions associated with host defense (61, 62). RA is a potential risk of cancer. Extensive use of biological agents may accelerate the induction of lymphoma, accompanied by demyelinating diseases of the central nervous system, elevated liver transaminases, and hyperlipidemia; however, the severity of the latter disease may be mild to moderate (63). Studies have reported that the use of IL-6 inhibitors may increase creatine kinase, although to a lesser extent than Janus kinase inhibitors (64). In some cases, the use of interleukin inhibitors reduces C-reactive protein levels, which may make the assessment of RA activity more difficult (65). In contrast to these adverse effects, the use of biological agents may prevent bone loss and bone erosion (66).

A strength of this study is the inclusion of the recently listed drug OKZ and the systematic and comprehensive comparison of a broad range of adverse events. However, this study has several limitations. For some rare events, such as tumors, tuberculosis, and death, short-term randomized controlled trials are not enough to evaluate the long-term safety of drugs. There was mild publication bias in the included literature, and a small number of results were heterogeneous. There was sparse network connections in the results of laboratory tests, and inconsistency could not be assessed using the node-splitting method, which may lead to model instability. The number of reported events such as death, tumors, poisoning, and injuries was small, which directly affects the results of head-to-head comparison. Long-term follow-up data were not included, and the results could only be used for the interpretation of the first treatment endpoint. The included literature was relatively old, and more recent evidence was limited. Further studies are needed to evaluate low-incidence adverse events.

## 5 Conclusion

Available randomized trial evidence suggests that adverse-event profiles differ among interleukin inhibitor regimens used for rheumatoid arthritis. However, comparative safety conclusions are limited by heterogeneous patient populations, variable background MTX/DMARD use, short trial durations, sparse rare-event data, and reliance on indirect comparisons. Sarilumab-containing regimens showed increased odds of several adverse-event outcomes in direct comparisons, including serious adverse events and ALT elevation, whereas death, malignancy, sepsis, and other rare outcomes remain too imprecise for firm conclusions. SUCRA rankings should be interpreted as exploratory rather than definitive. Further long-term, adequately powered comparative safety studies are needed.

## Data Availability

The raw data supporting the conclusions of this article will be made available by the authors, without undue reservation.
