# Supplementary material for: Comparative safety of interleukin inhibitors for rheumatoid arthritis: a systematic review and network meta-analysis of randomized controlled trials
Source: Front Immunol. 2026 Sep 23;17:1866093. doi: 10.3389/fimmu.2026.1866093 (PMC13642689; doi:10.3389/fimmu.2026.1866093)
Supplement: Supplementary file 1 [file DataSheet1.pdf]

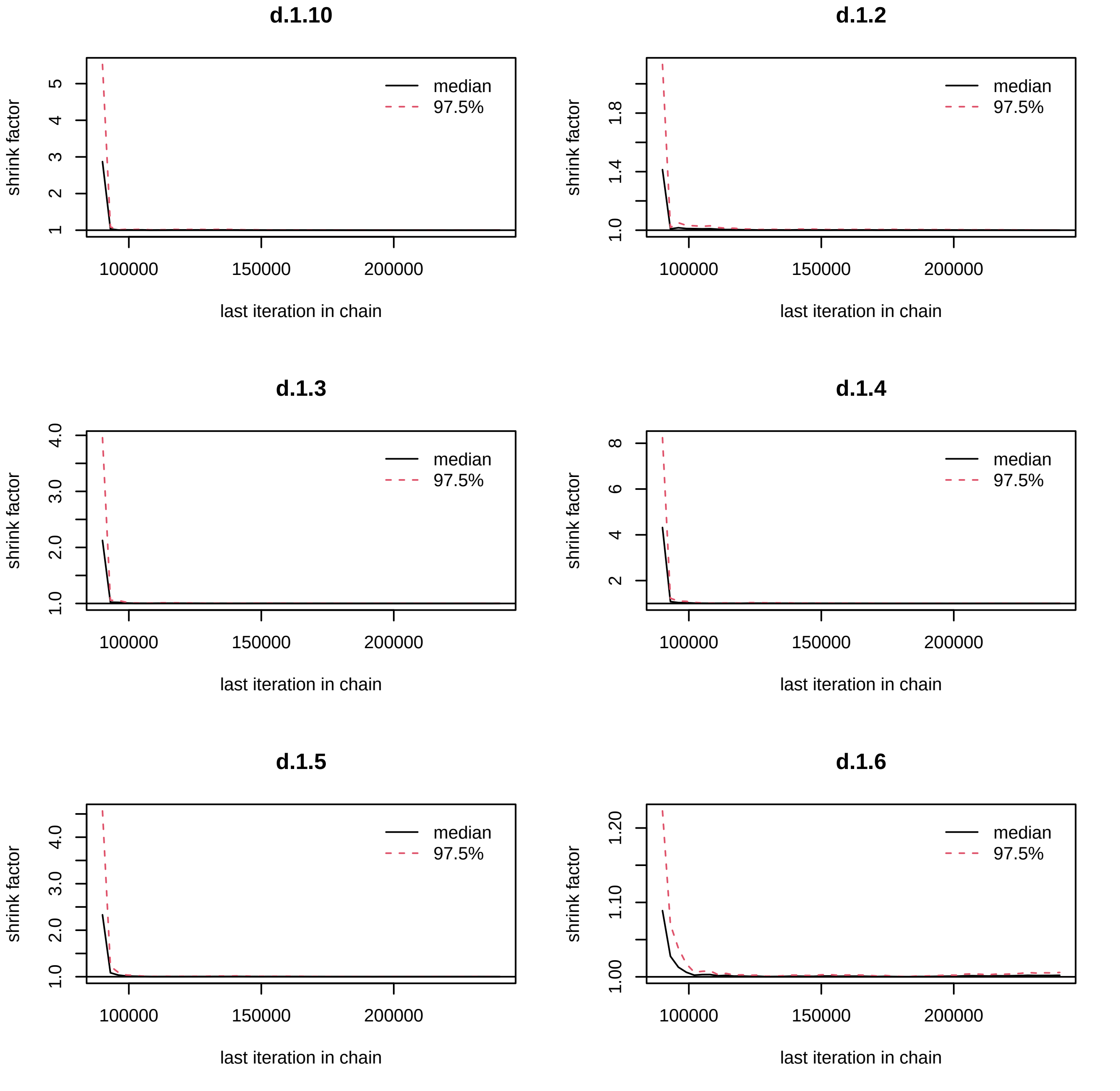
d.1.10
shrink factor
1
2
3
4
5
median
97.5%
100000
150000
200000
last iteration in chain
d.1.2
shrink factor
1.0
1.4
1.8
median
97.5%
100000
150000
200000
last iteration in chain
d.1.3
shrink factor
1.0
2.0
3.0
4.0
median
97.5%
100000
150000
200000
last iteration in chain
d.1.4
shrink factor
2
4
6
8
median
97.5%
100000
150000
200000
last iteration in chain
d.1.5
shrink factor
1.0
2.0
3.0
4.0
median
97.5%
100000
150000
200000
last iteration in chain
d.1.6
shrink factor
1.00
1.10
1.20
median
97.5%
100000
150000
200000
last iteration in chain

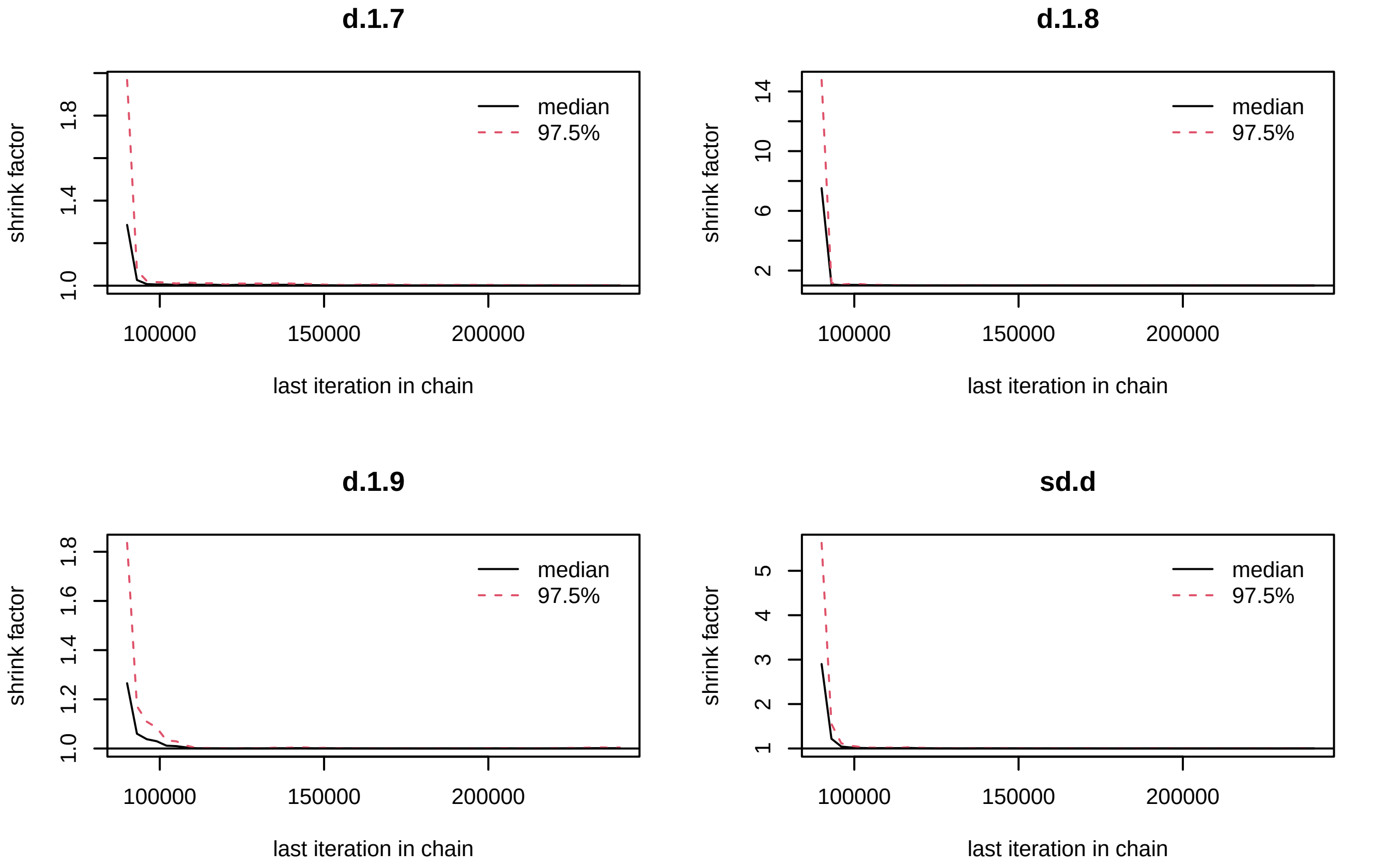

d.1.7
shrink factor
1.0
1.4
1.8
median
97.5%
100000
150000
200000
last iteration in chain
d.1.8
shrink factor
2
6
10
14
median
97.5%
100000
150000
200000
last iteration in chain
d.1.9
shrink factor
1.0
1.2
1.4
1.6
1.8
median
97.5%
100000
150000
200000
last iteration in chain
sd.d
shrink factor
1
2
3
4
5
median
97.5%
100000
150000
200000
last iteration in chain
