# Supplementary material for: Comparative safety of interleukin inhibitors for rheumatoid arthritis: a systematic review and network meta-analysis of randomized controlled trials
Source: Front Immunol. 2026 Sep 23;17:1866093. doi: 10.3389/fimmu.2026.1866093 (PMC13642689; doi:10.3389/fimmu.2026.1866093)
Supplement: Supplementary file 2 [file DataSheet10.pdf]

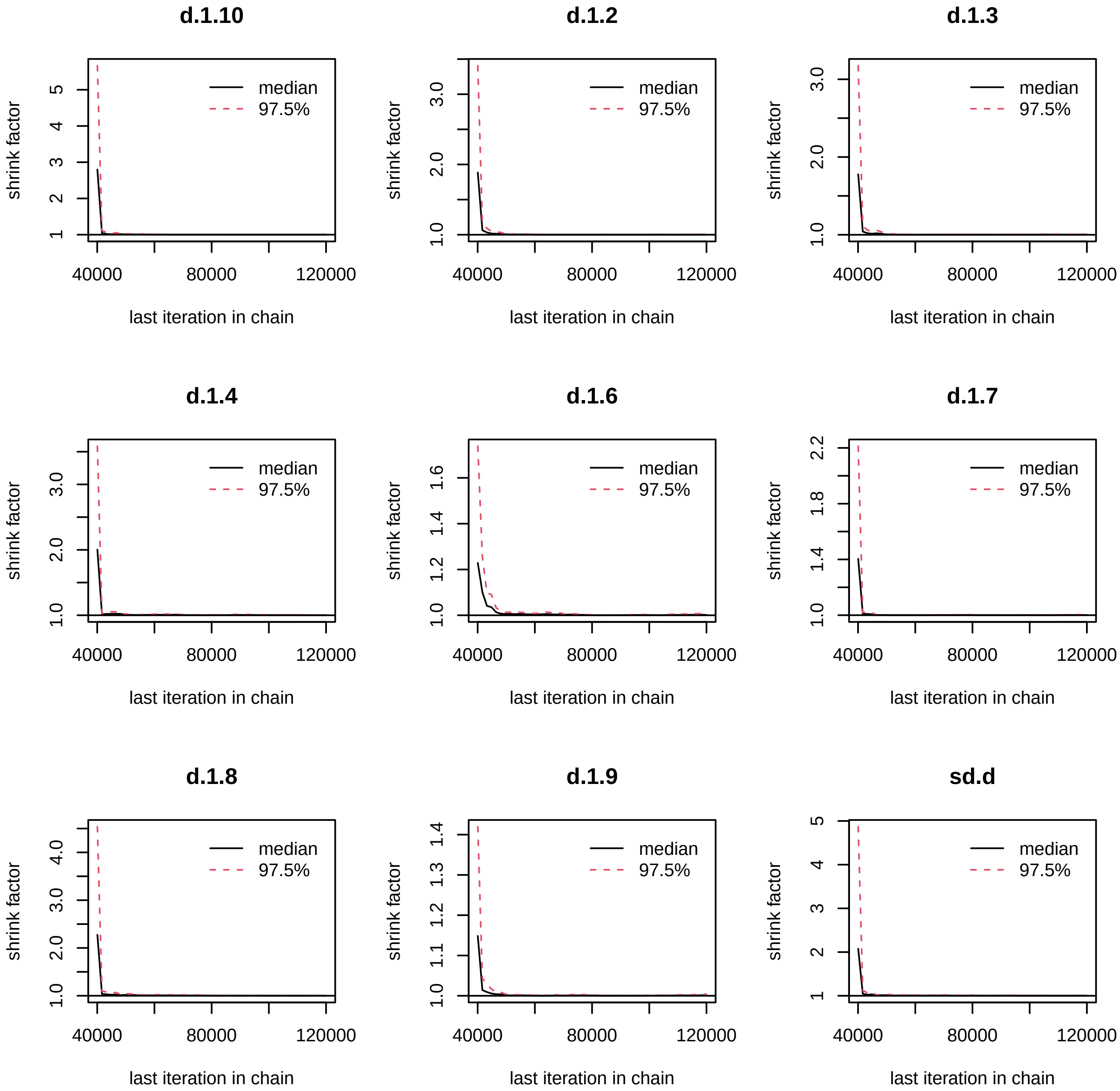
d.1.10
median
97.5%
shrink factor
1
2
3
4
5
40000
80000
120000
last iteration in chain
d.1.2
median
97.5%
shrink factor
1.0
2.0
3.0
40000
80000
120000
last iteration in chain
d.1.3
median
97.5%
shrink factor
1.0
2.0
3.0
40000
80000
120000
last iteration in chain
d.1.4
median
97.5%
shrink factor
1.0
2.0
3.0
40000
80000
120000
last iteration in chain
d.1.6
median
97.5%
shrink factor
1.0
1.2
1.4
1.6
40000
80000
120000
last iteration in chain
d.1.7
median
97.5%
shrink factor
1.0
1.4
1.8
2.2
40000
80000
120000
last iteration in chain
d.1.8
median
97.5%
shrink factor
1.0
2.0
3.0
4.0
40000
80000
120000
last iteration in chain
d.1.9
median
97.5%
shrink factor
1.0
1.1
1.2
1.3
1.4
40000
80000
120000
last iteration in chain
sd.d
median
97.5%
shrink factor
1
2
3
4
5
40000
80000
120000
last iteration in chain
