# Supplementary material for: Comparative safety of interleukin inhibitors for rheumatoid arthritis: a systematic review and network meta-analysis of randomized controlled trials
Source: Front Immunol. 2026 Sep 23;17:1866093. doi: 10.3389/fimmu.2026.1866093 (PMC13642689; doi:10.3389/fimmu.2026.1866093)
Supplement: Supplementary file 3 [file DataSheet11.pdf]

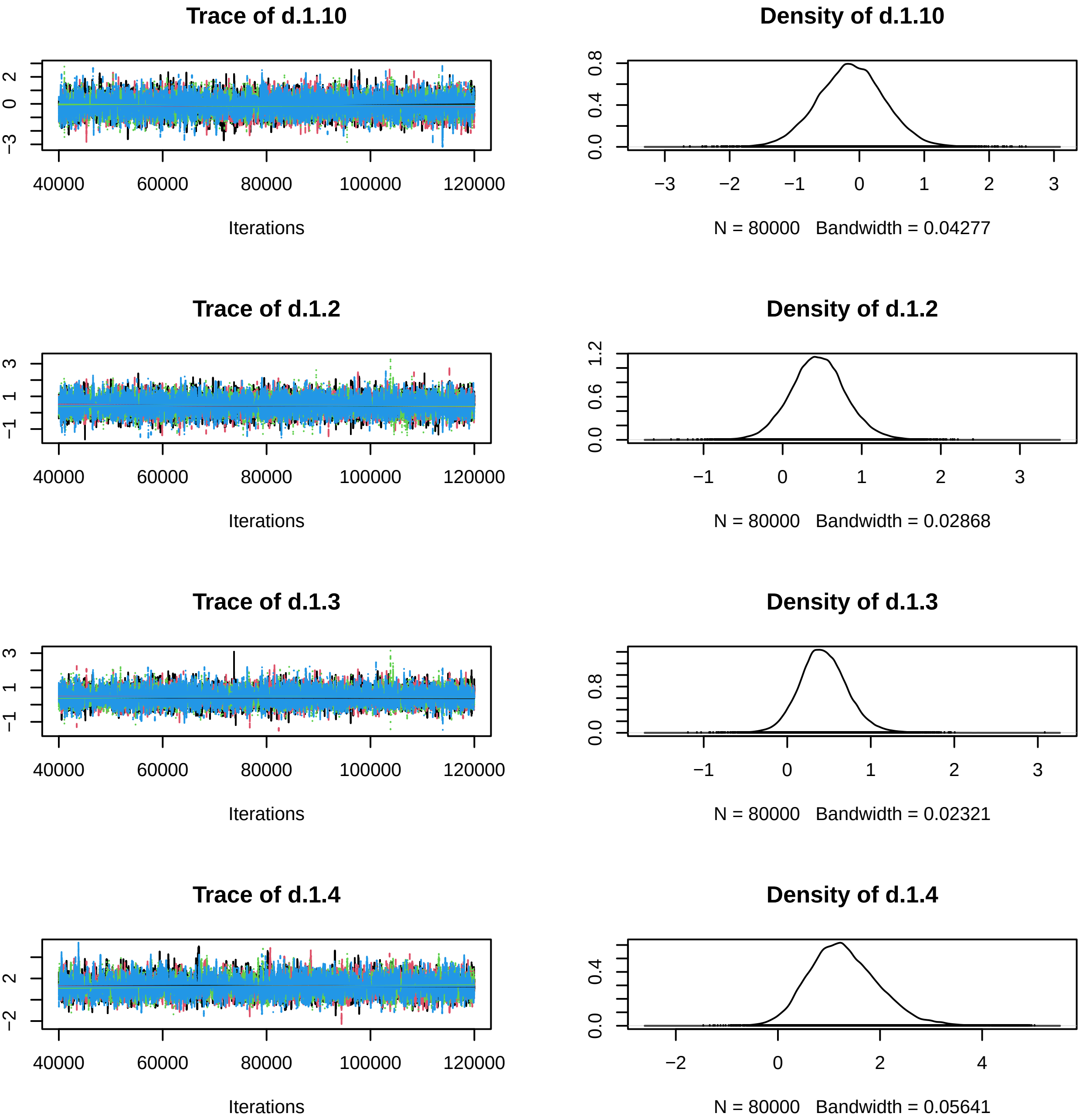
Trace of d.1.10
Iterations
Density of d.1.10
N = 80000 Bandwidth = 0.04277
Trace of d.1.2
Iterations
Density of d.1.2
N = 80000 Bandwidth = 0.02868
Trace of d.1.3
Iterations
Density of d.1.3
N = 80000 Bandwidth = 0.02321
Trace of d.1.4
Iterations
Density of d.1.4
N = 80000 Bandwidth = 0.05641

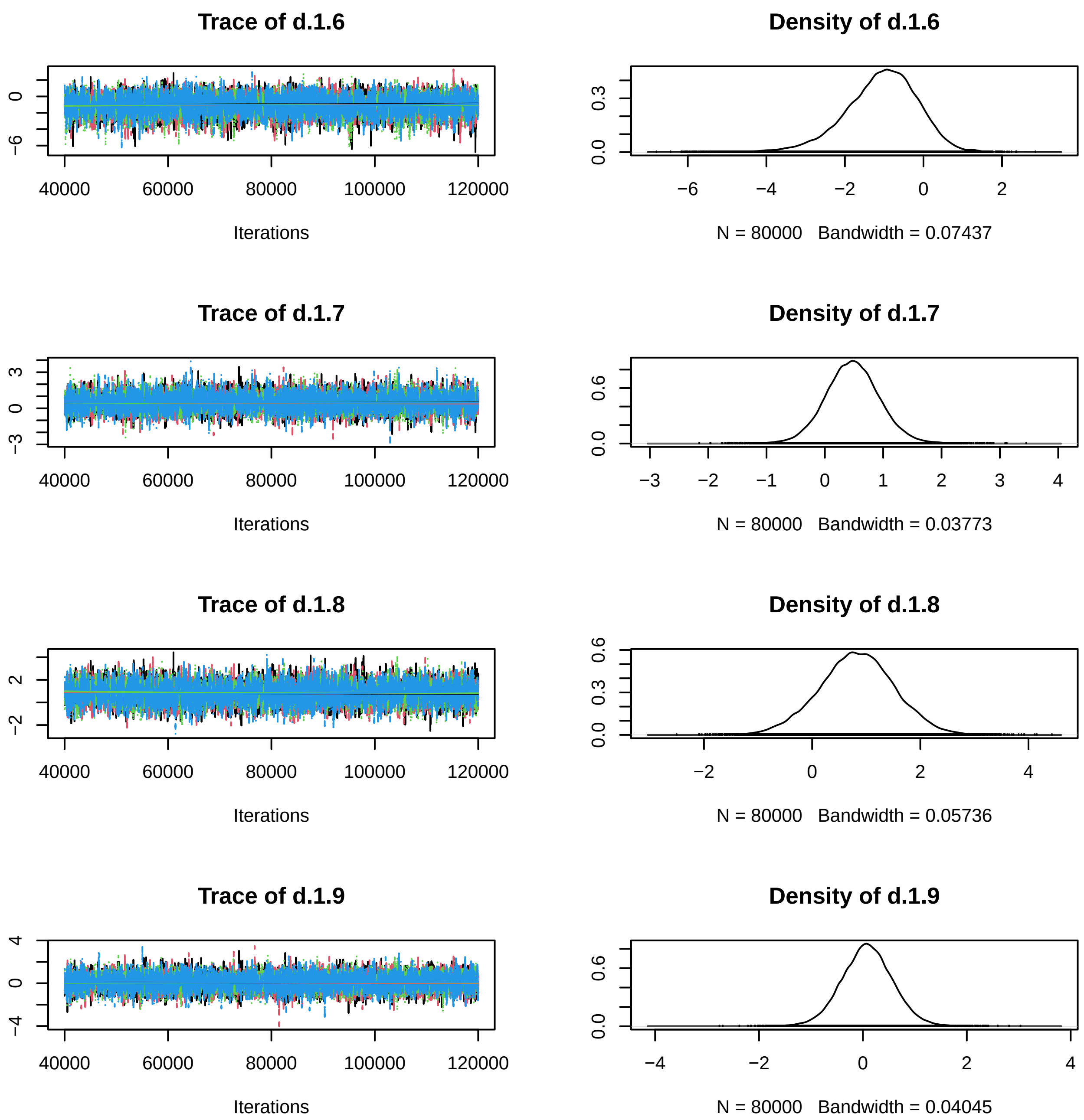

Trace of d.1.6
Iterations
Density of d.1.6
N = 80000 Bandwidth = 0.07437
Trace of d.1.7
Iterations
Density of d.1.7
N = 80000 Bandwidth = 0.03773
Trace of d.1.8
Iterations
Density of d.1.8
N = 80000 Bandwidth = 0.05736
Trace of d.1.9
Iterations
Density of d.1.9
N = 80000 Bandwidth = 0.04045

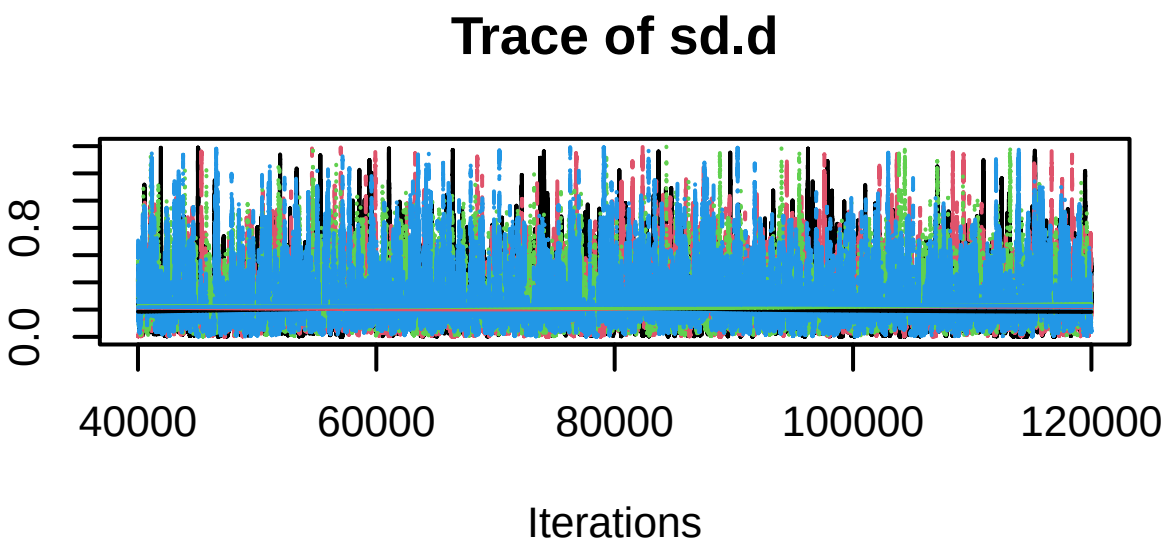
Trace of sd.d
0.0
0.8
40000
60000
80000
100000
120000
Iterations

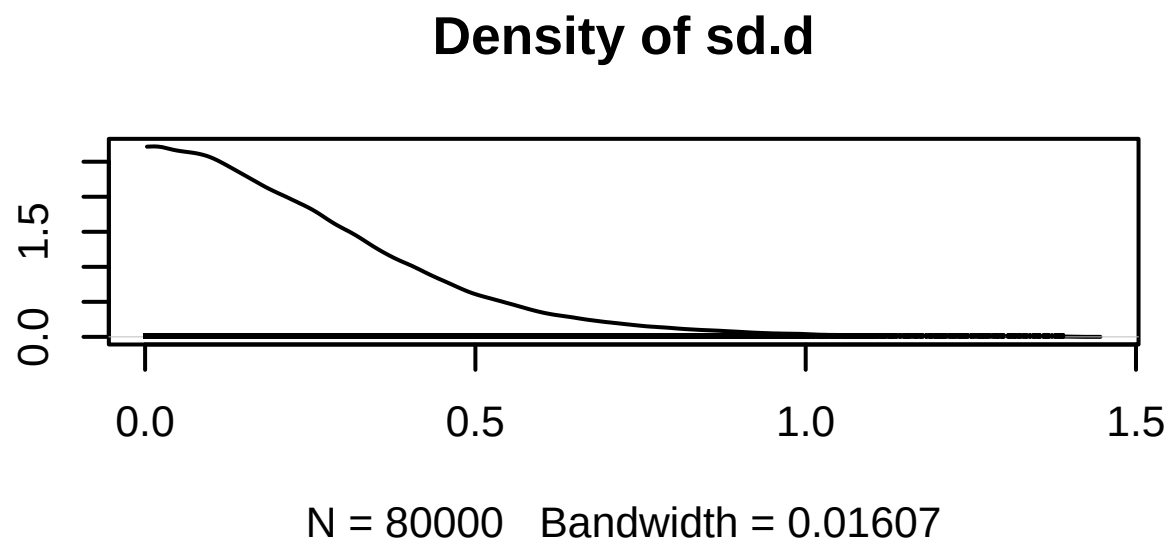
Density of sd.d
0.0
1.5
0.0
0.5
1.0
1.5
N = 80000 Bandwidth = 0.01607
