# Supplementary material for: Comparative safety of interleukin inhibitors for rheumatoid arthritis: a systematic review and network meta-analysis of randomized controlled trials
Source: Front Immunol. 2026 Sep 23;17:1866093. doi: 10.3389/fimmu.2026.1866093 (PMC13642689; doi:10.3389/fimmu.2026.1866093)
Supplement: Supplementary file 4 [file DataSheet12.pdf]

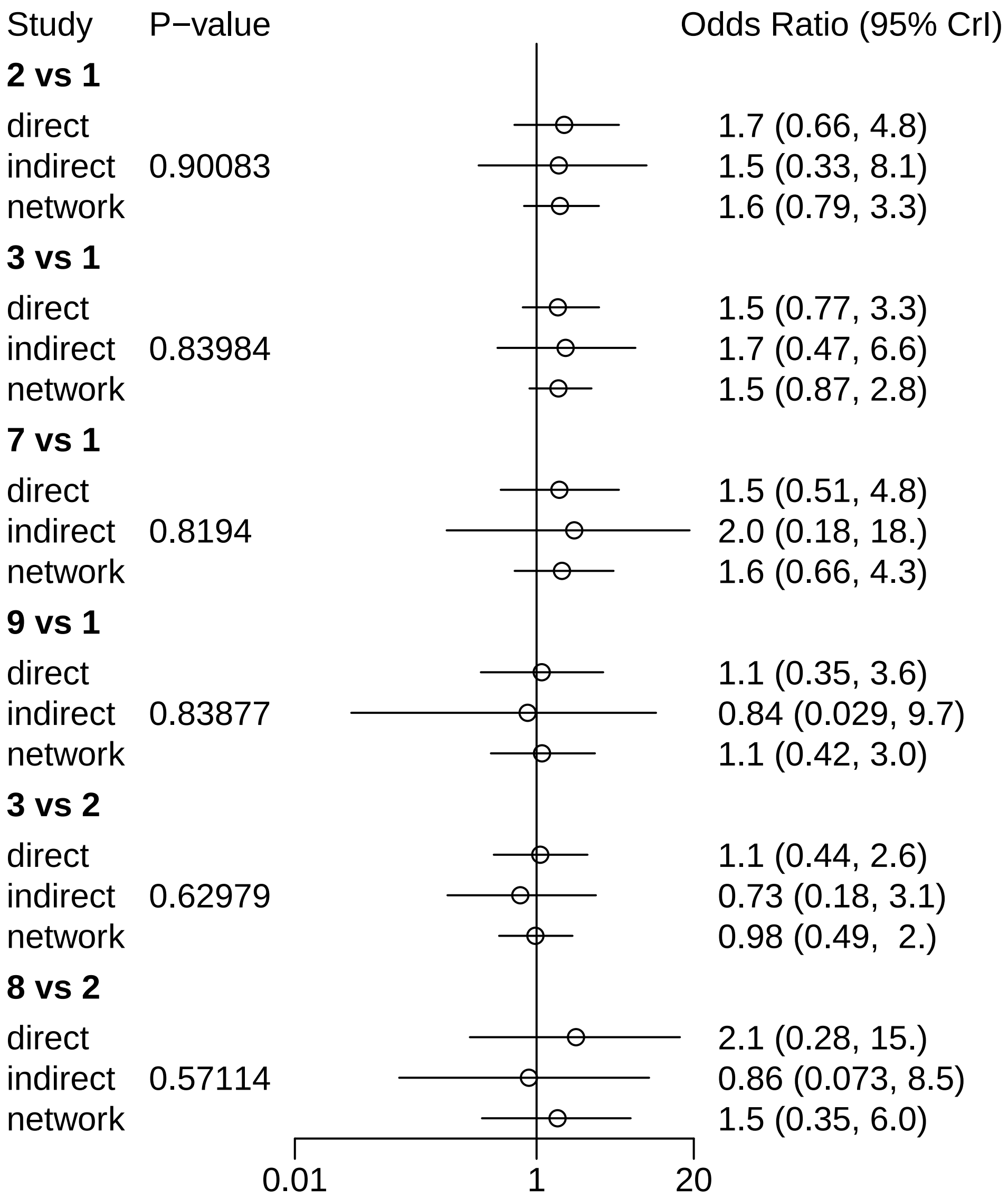

Study
P−value
Odds Ratio (95% CrI)
2 vs 1
direct
1.7 (0.66, 4.8)
indirect
0.90083
1.5 (0.33, 8.1)
network
1.6 (0.79, 3.3)
3 vs 1
direct
1.5 (0.77, 3.3)
indirect
0.83984
1.7 (0.47, 6.6)
network
1.5 (0.87, 2.8)
7 vs 1
direct
1.5 (0.51, 4.8)
indirect
0.8194
2.0 (0.18, 18.)
network
1.6 (0.66, 4.3)
9 vs 1
direct
1.1 (0.35, 3.6)
indirect
0.83877
0.84 (0.029, 9.7)
network
1.1 (0.42, 3.0)
3 vs 2
direct
1.1 (0.44, 2.6)
indirect
0.62979
0.73 (0.18, 3.1)
network
0.98 (0.49, 2.)
8 vs 2
direct
2.1 (0.28, 15.)
indirect
0.57114
0.86 (0.073, 8.5)
network
1.5 (0.35, 6.0)
0.01
1
20

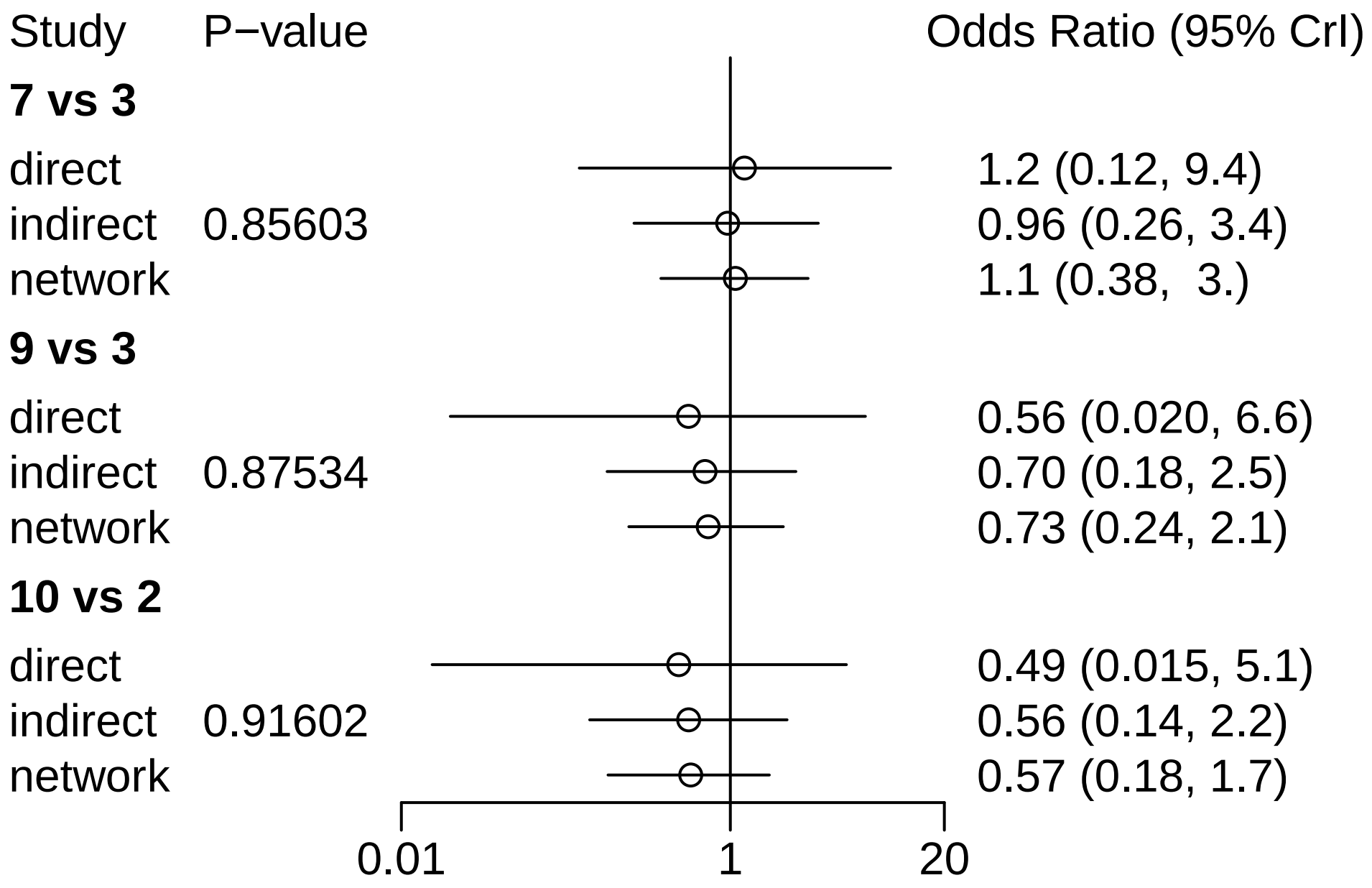

Study
P−value
Odds Ratio (95% CrI)
7 vs 3
direct
1.2 (0.12, 9.4)
indirect
0.85603
0.96 (0.26, 3.4)
network
1.1 (0.38, 3.)
9 vs 3
direct
0.56 (0.020, 6.6)
indirect
0.87534
0.70 (0.18, 2.5)
network
0.73 (0.24, 2.1)
10 vs 2
direct
0.49 (0.015, 5.1)
indirect
0.91602
0.56 (0.14, 2.2)
network
0.57 (0.18, 1.7)
0.01
1
20
