# Supplementary material for: Comparative safety of interleukin inhibitors for rheumatoid arthritis: a systematic review and network meta-analysis of randomized controlled trials
Source: Front Immunol. 2026 Sep 23;17:1866093. doi: 10.3389/fimmu.2026.1866093 (PMC13642689; doi:10.3389/fimmu.2026.1866093)
Supplement: Supplementary file 5 [file DataSheet13.pdf]

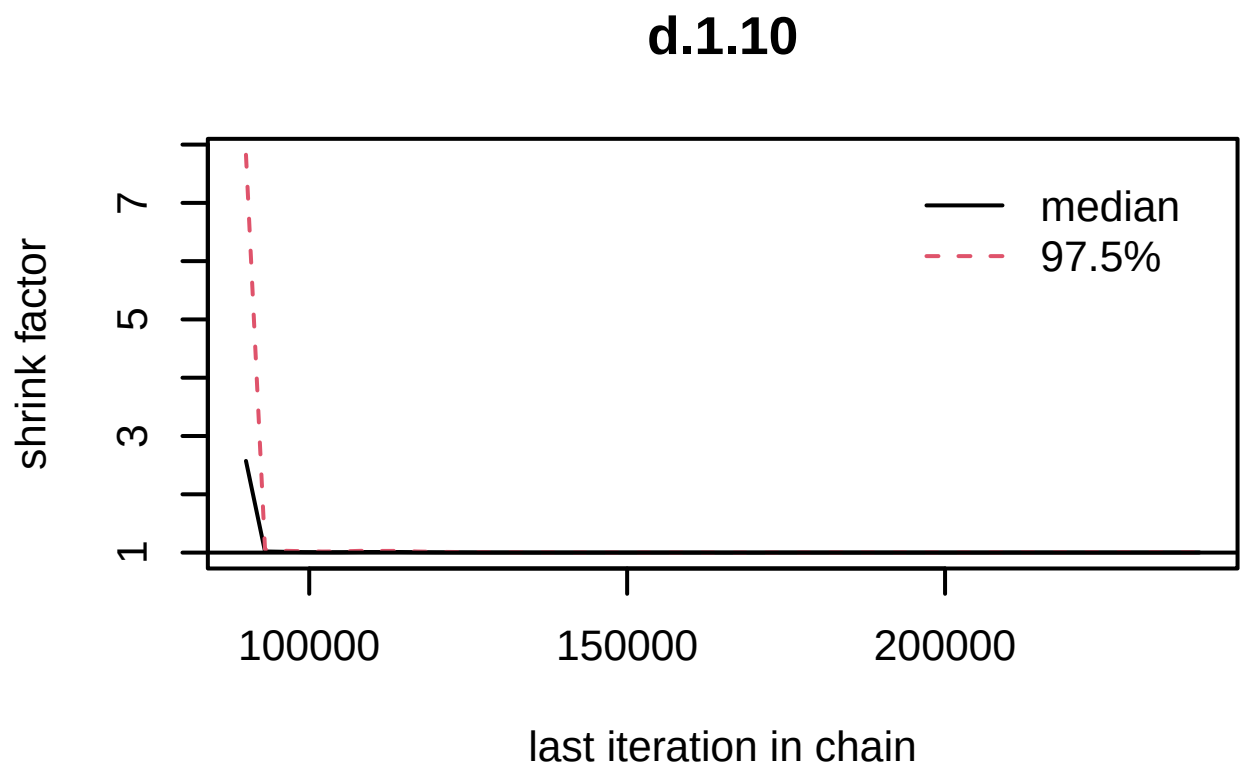
d.1.10
shrink factor
1
3
5
7
median
97.5%
100000
150000
200000
last iteration in chain

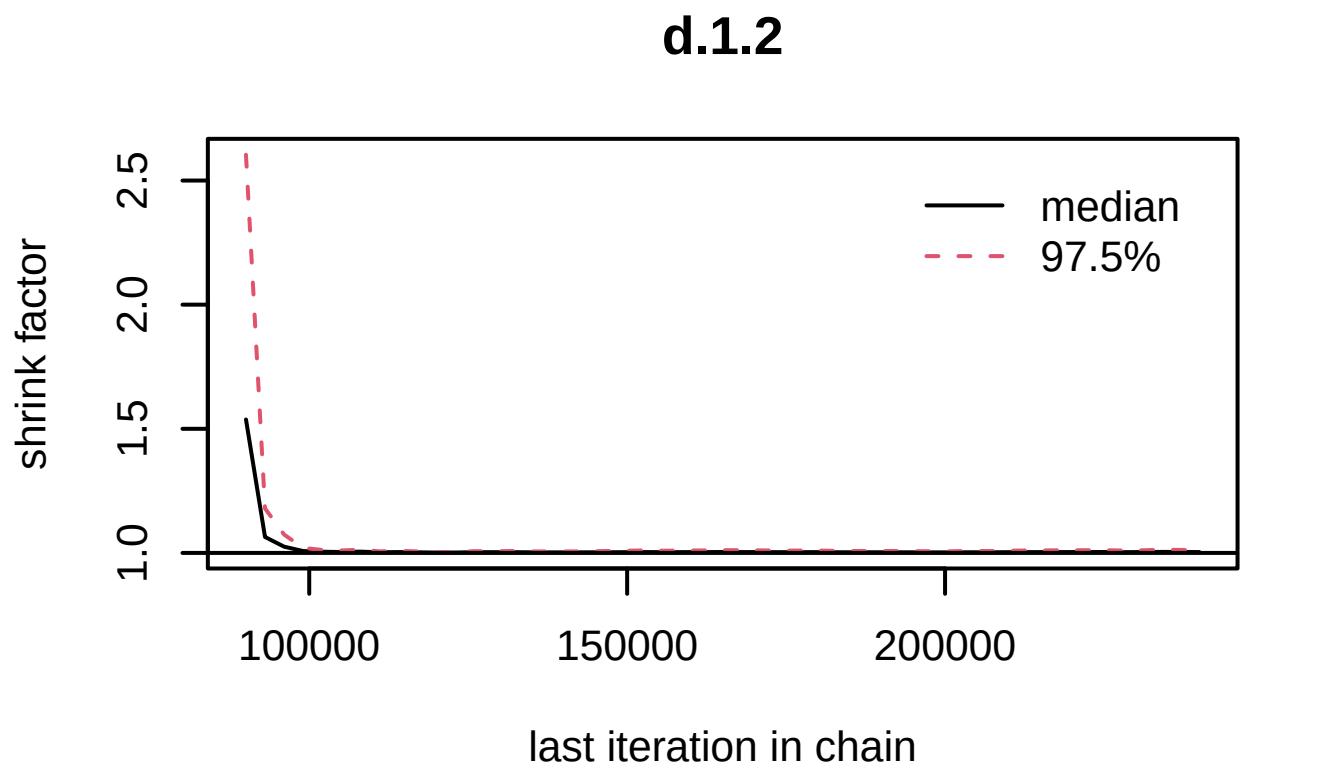
d.1.2
shrink factor
1.0
1.5
2.0
2.5
median
97.5%
100000
150000
200000
last iteration in chain

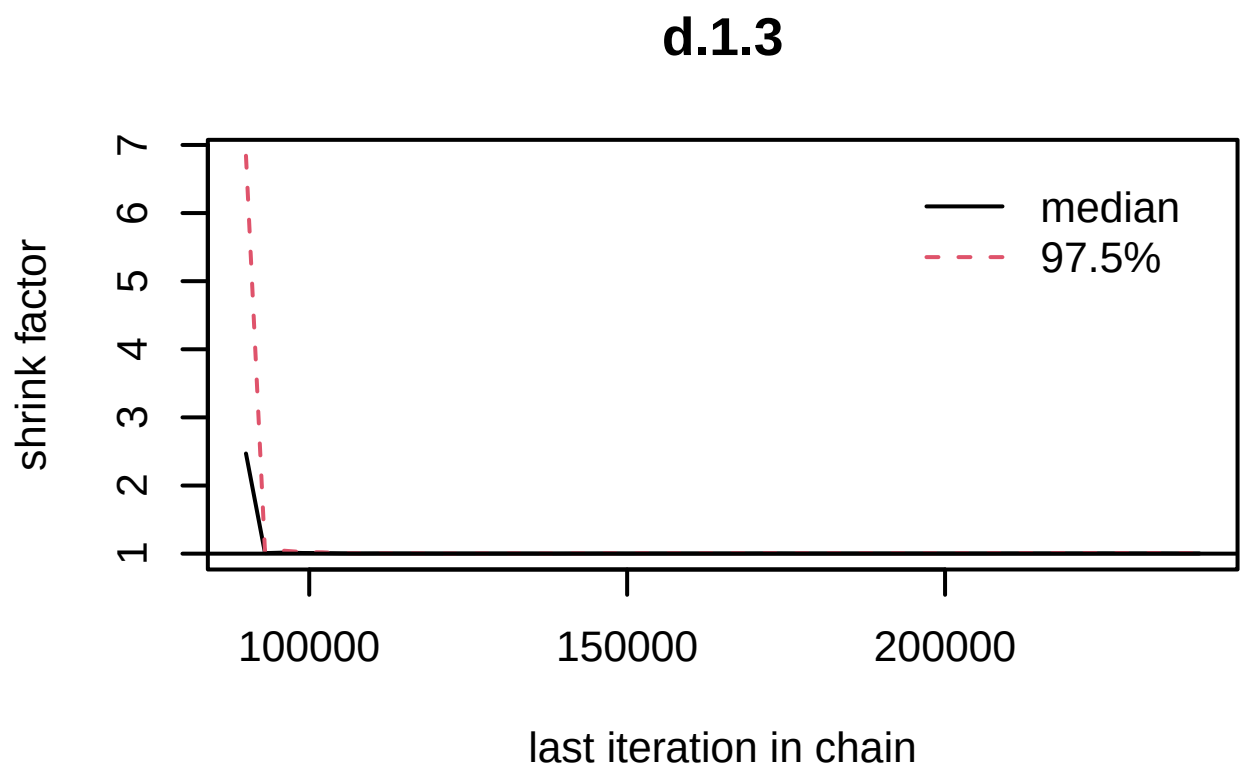
d.1.3
shrink factor
1
2
3
4
5
6
7
median
97.5%
100000
150000
200000
last iteration in chain

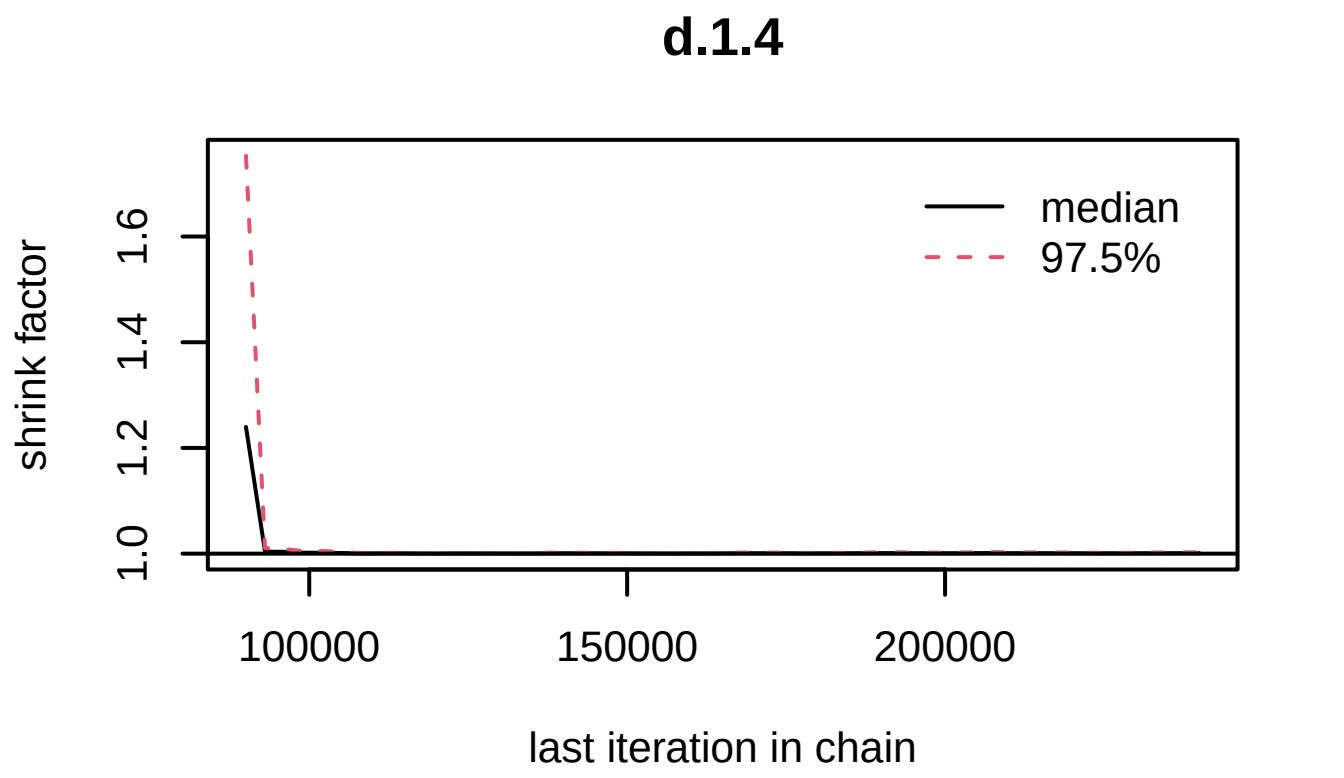
d.1.4
shrink factor
1.0
1.2
1.4
1.6
median
97.5%
100000
150000
200000
last iteration in chain

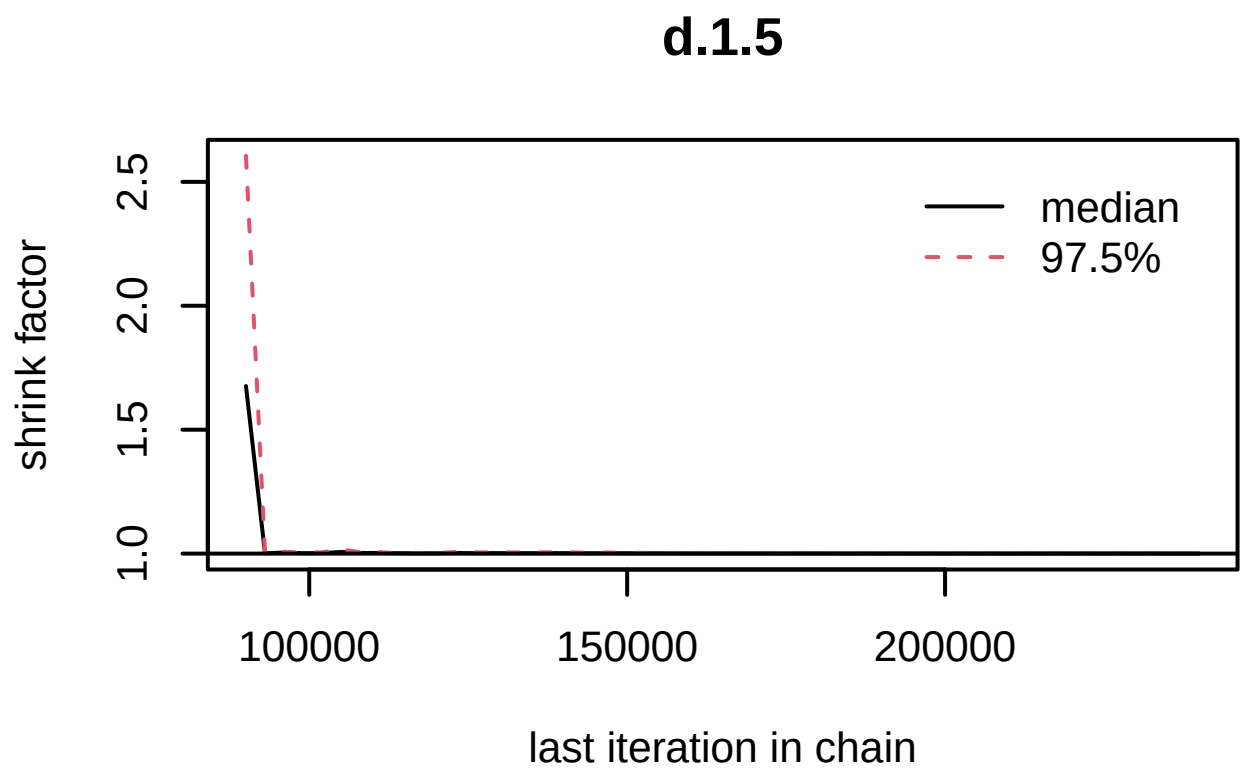
d.1.5
shrink factor
1.0
1.5
2.0
2.5
median
97.5%
100000
150000
200000
last iteration in chain

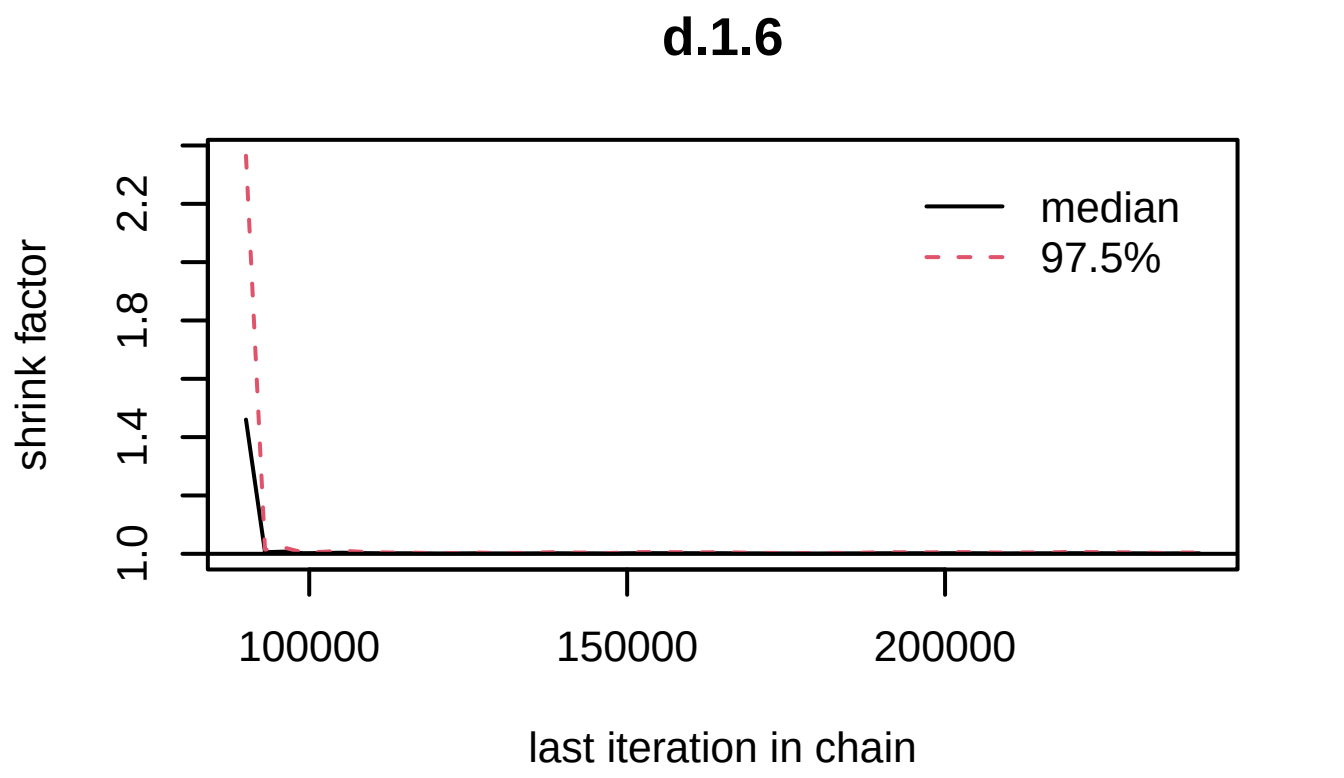
d.1.6
shrink factor
1.0
1.4
1.8
2.2
median
97.5%
100000
150000
200000
last iteration in chain

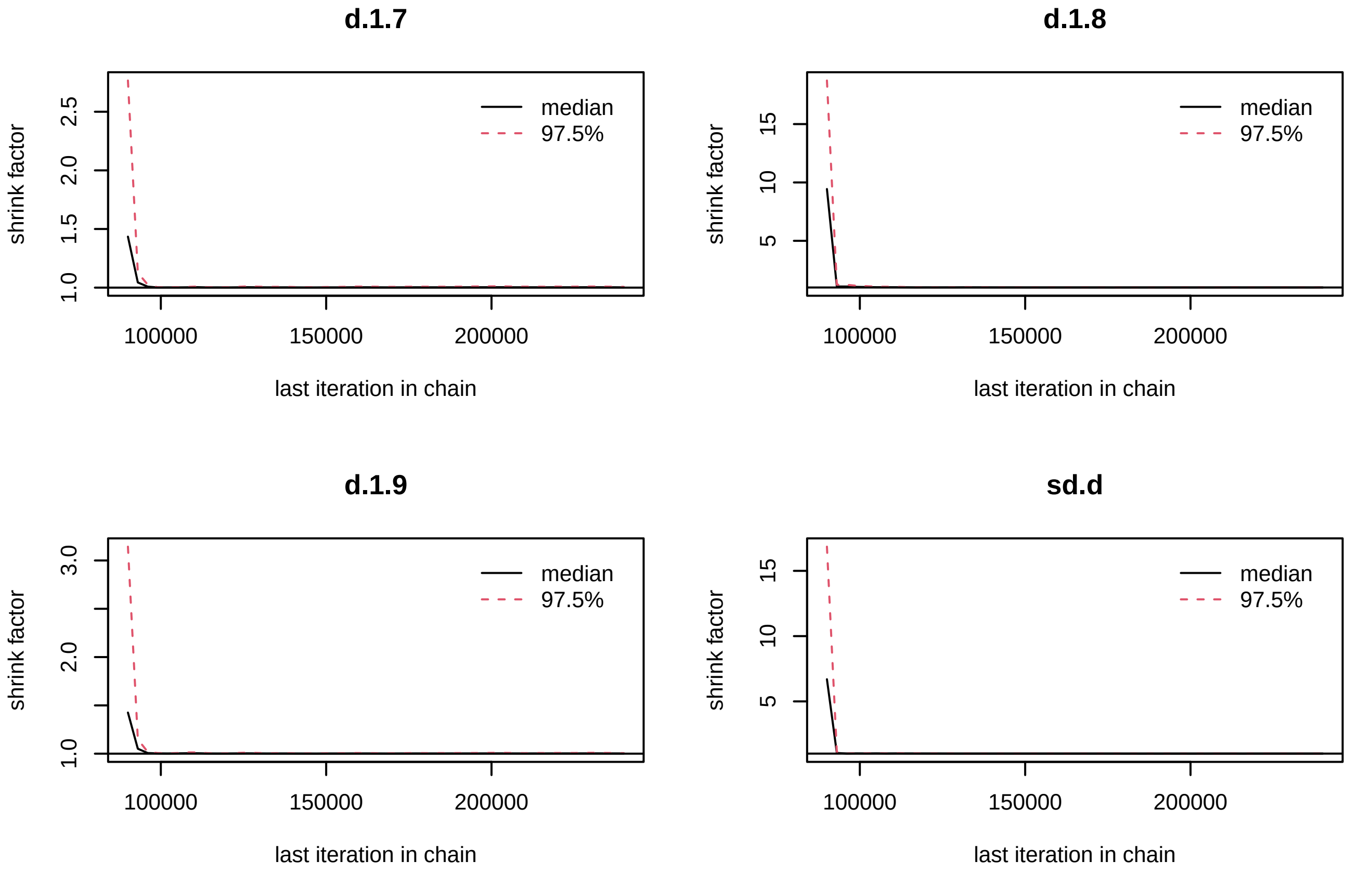
d.1.7
d.1.8
d.1.9
sd.d
median
97.5%
shrink factor
last iteration in chain
100000
150000
200000
1.0
1.5
2.0
2.5
3.0
5
10
15
