# Supplementary material for: Comparative safety of interleukin inhibitors for rheumatoid arthritis: a systematic review and network meta-analysis of randomized controlled trials
Source: Front Immunol. 2026 Sep 23;17:1866093. doi: 10.3389/fimmu.2026.1866093 (PMC13642689; doi:10.3389/fimmu.2026.1866093)
Supplement: Supplementary file 6 [file DataSheet14.pdf]

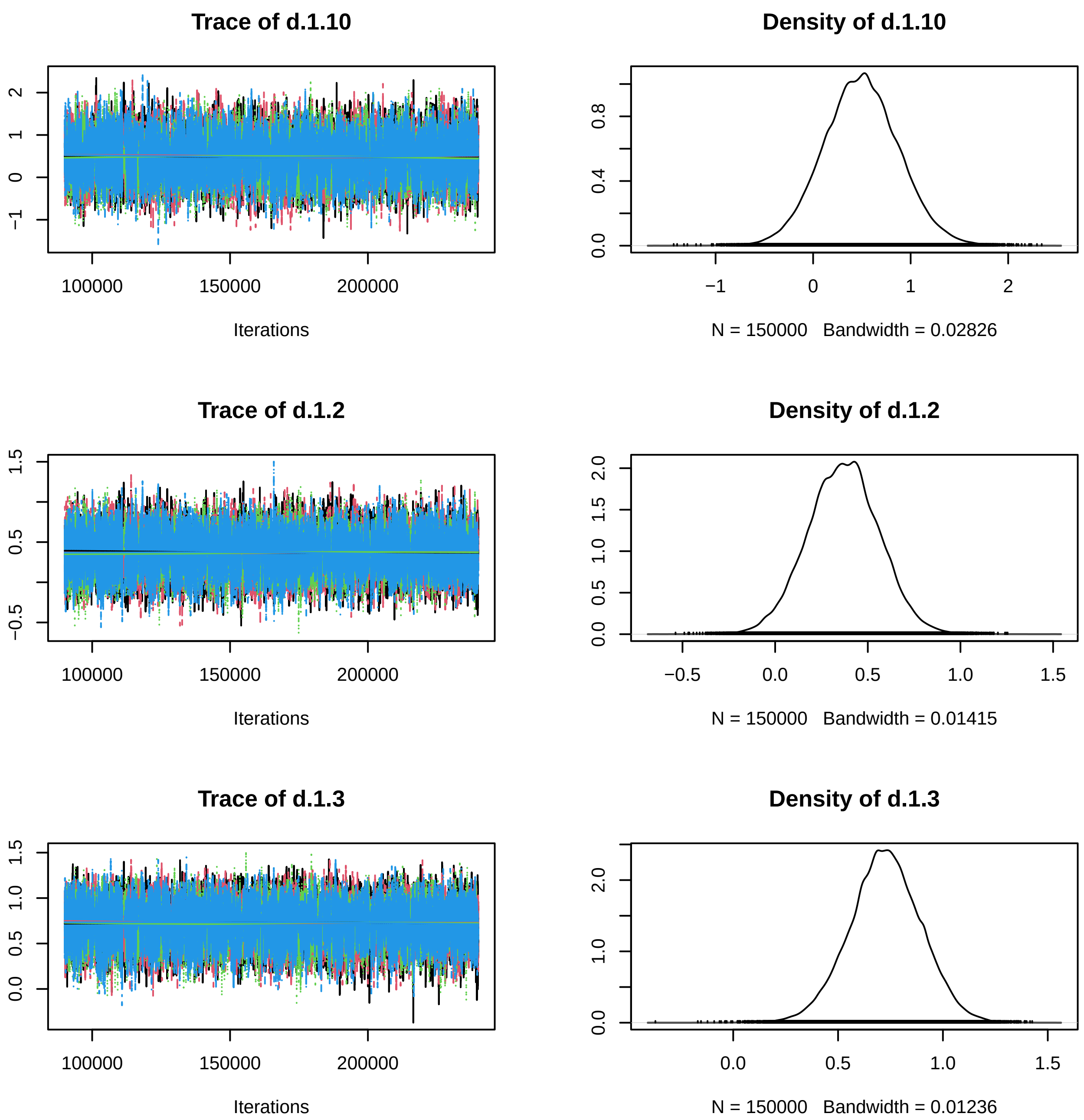
Trace of d.1.10
Iterations
Density of d.1.10
N = 150000 Bandwidth = 0.02826
Trace of d.1.2
Iterations
Density of d.1.2
N = 150000 Bandwidth = 0.01415
Trace of d.1.3
Iterations
Density of d.1.3
N = 150000 Bandwidth = 0.01236

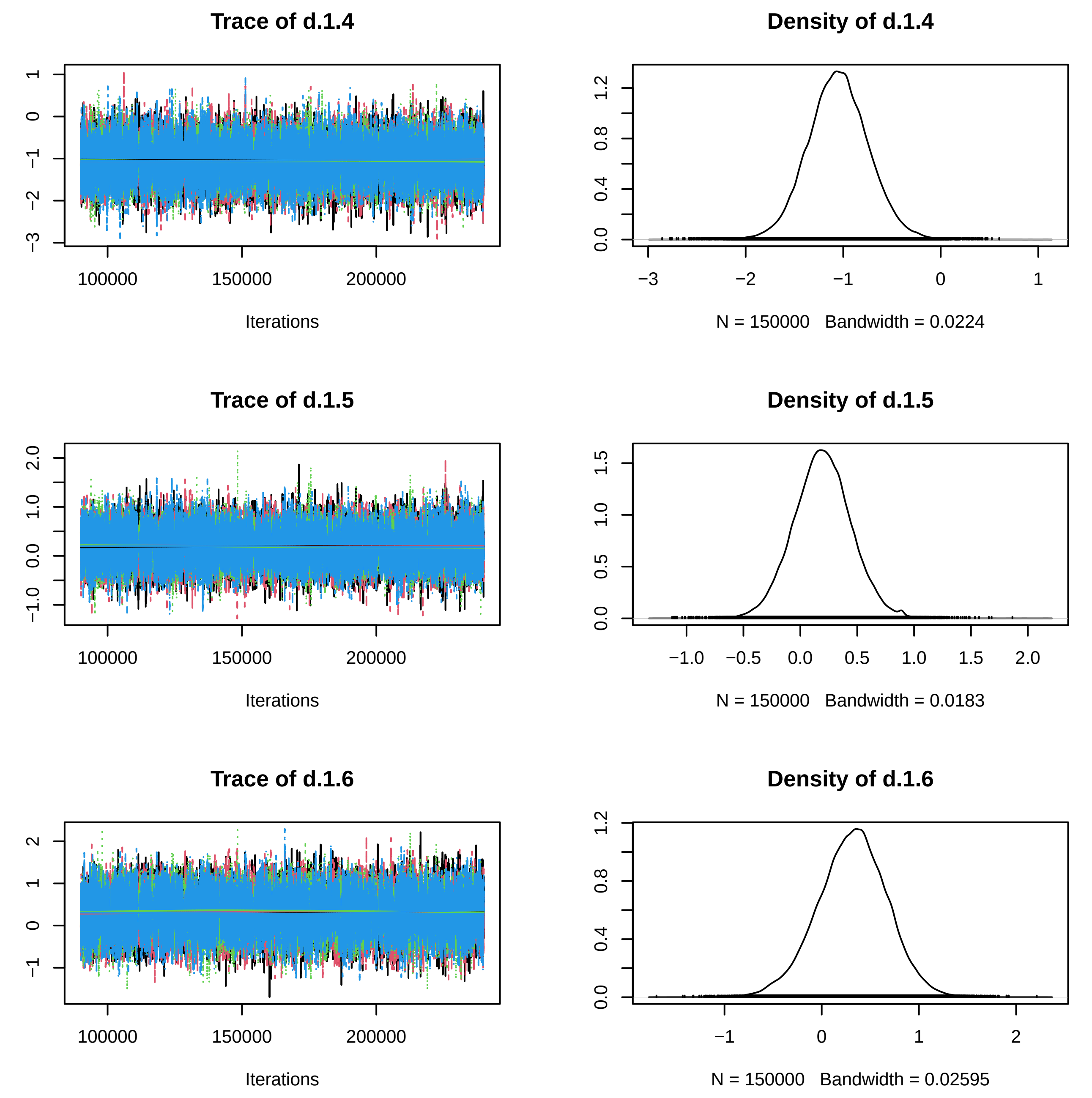
Trace of d.1.4
Density of d.1.4
Iterations
N = 150000 Bandwidth = 0.0224
Trace of d.1.5
Density of d.1.5
Iterations
N = 150000 Bandwidth = 0.0183
Trace of d.1.6
Density of d.1.6
Iterations
N = 150000 Bandwidth = 0.02595
100000
150000
200000

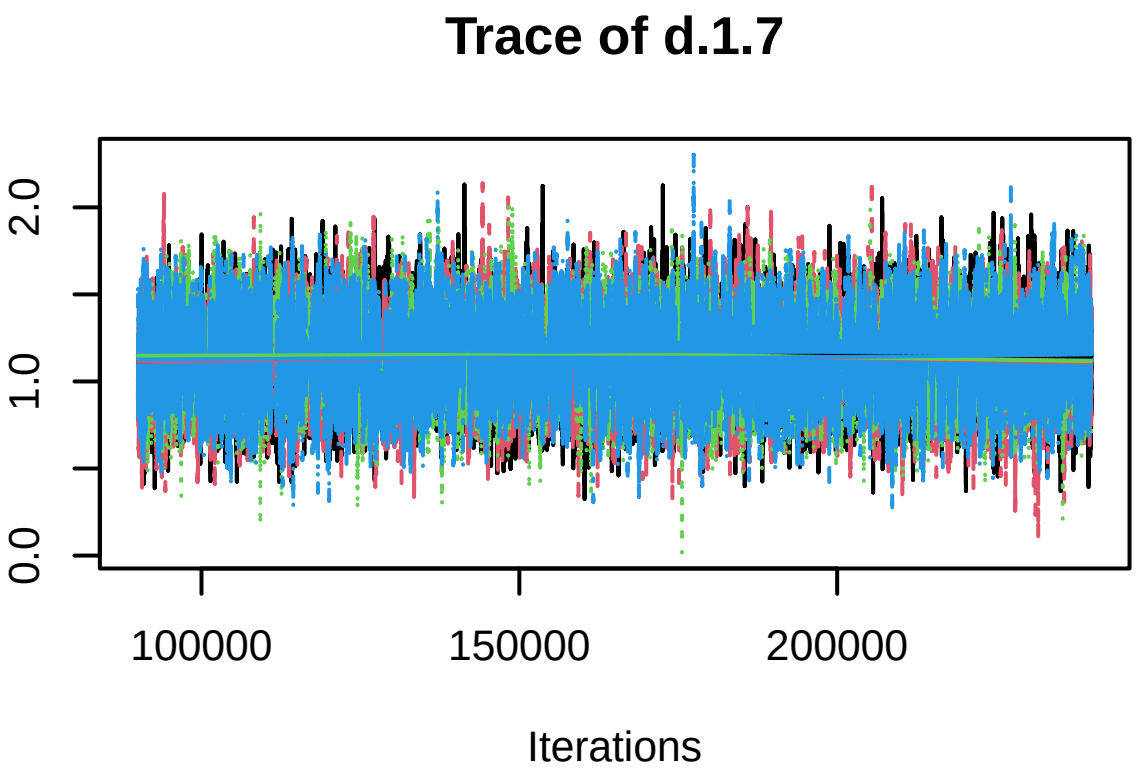
Trace of d.1.7
0.0
1.0
2.0
100000
150000
200000
Iterations
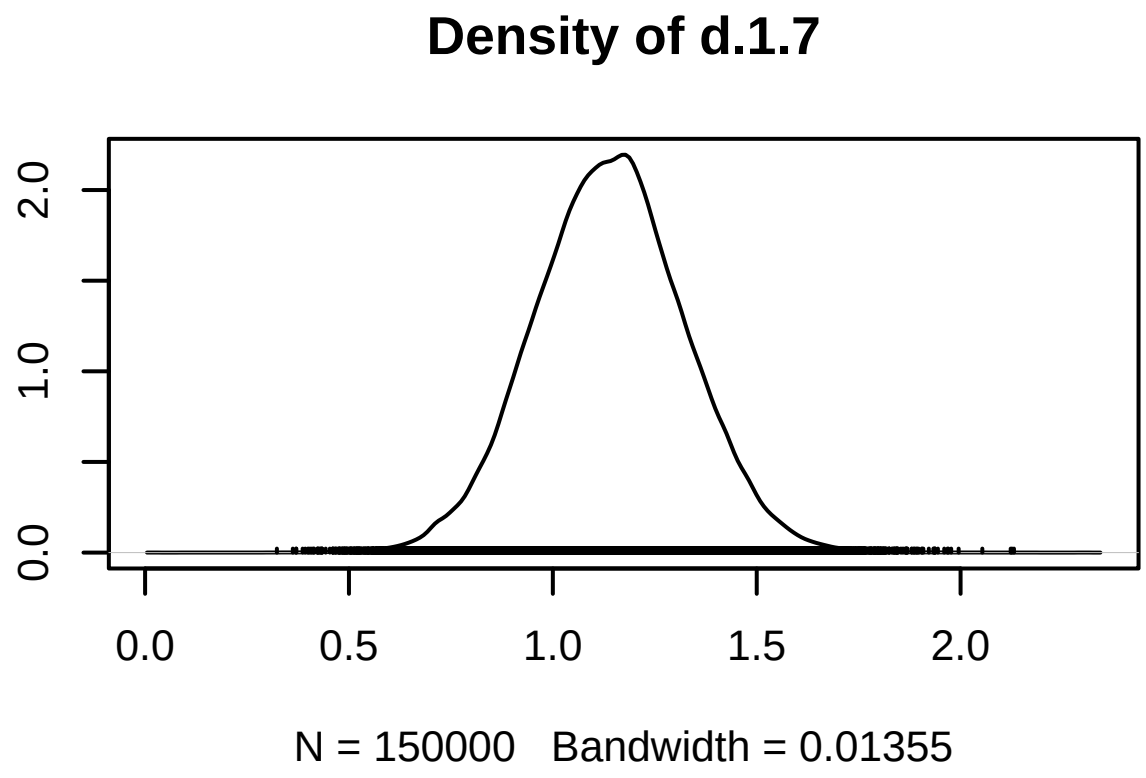
Density of d.1.7
0.0
1.0
2.0
0.0
0.5
1.0
1.5
2.0
N = 150000 Bandwidth = 0.01355
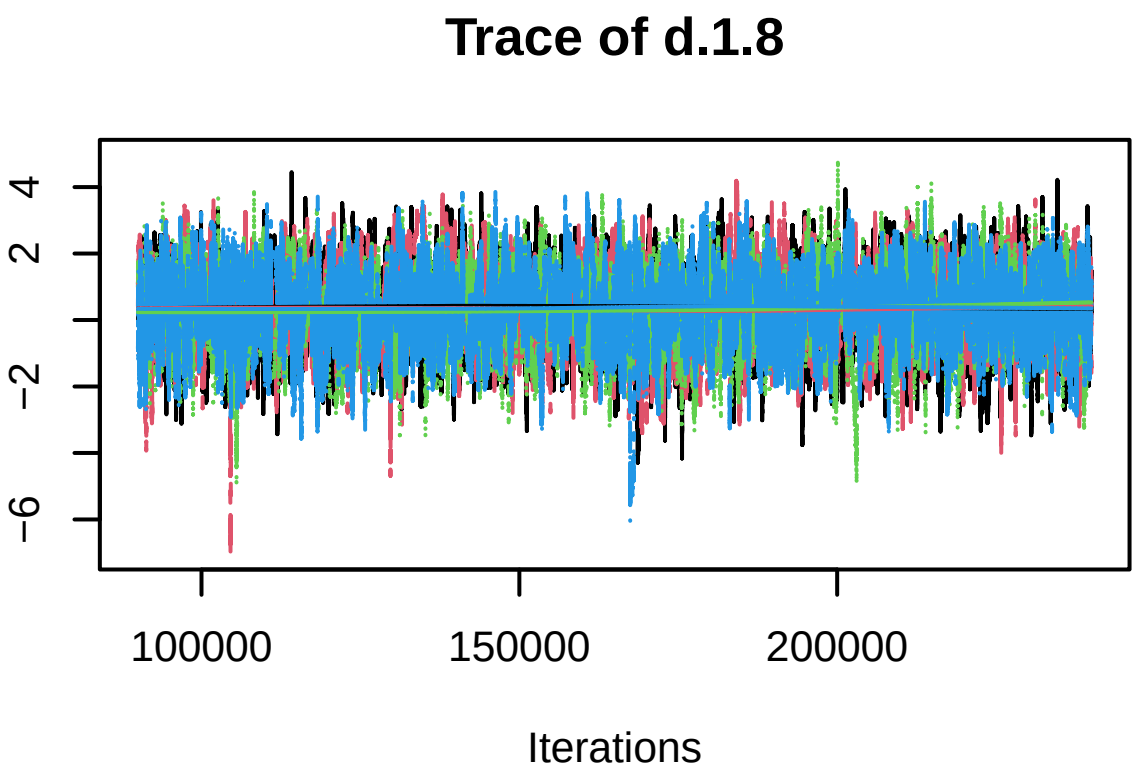
Trace of d.1.8
−6
−2
2
4
100000
150000
200000
Iterations
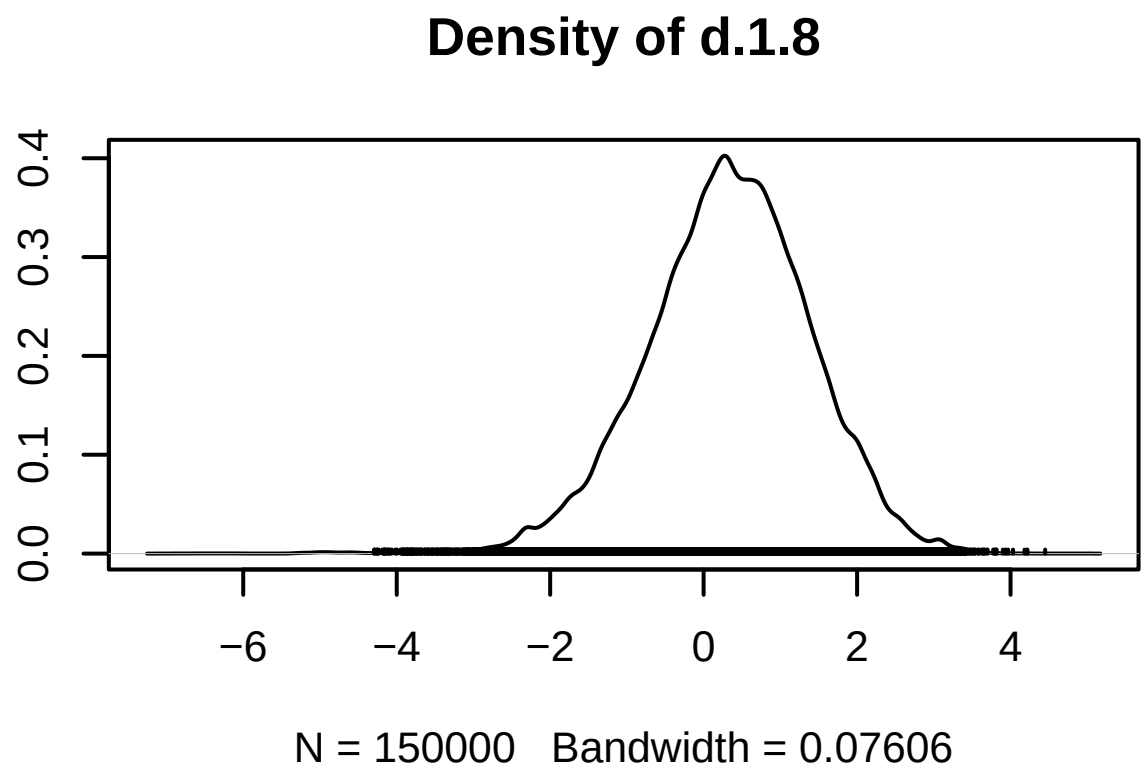
Density of d.1.8
0.0
0.1
0.2
0.3
0.4
−6
−4
−2
0
2
4
N = 150000 Bandwidth = 0.07606
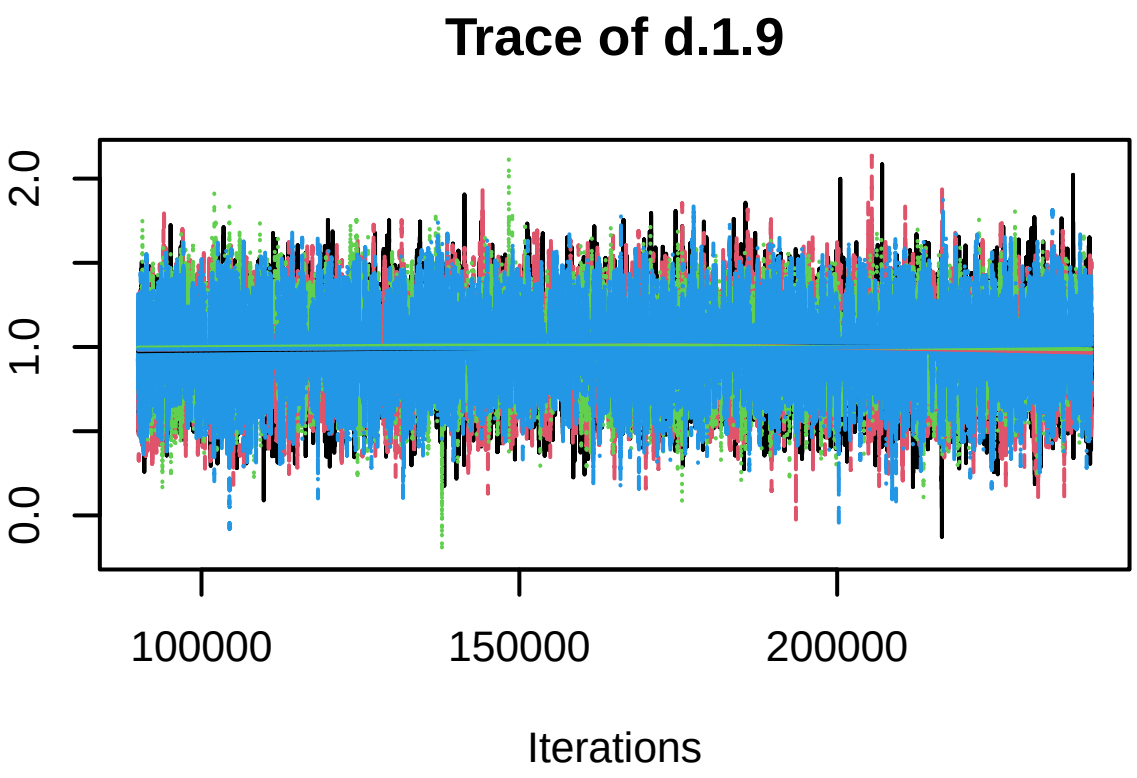
Trace of d.1.9
0.0
1.0
2.0
100000
150000
200000
Iterations
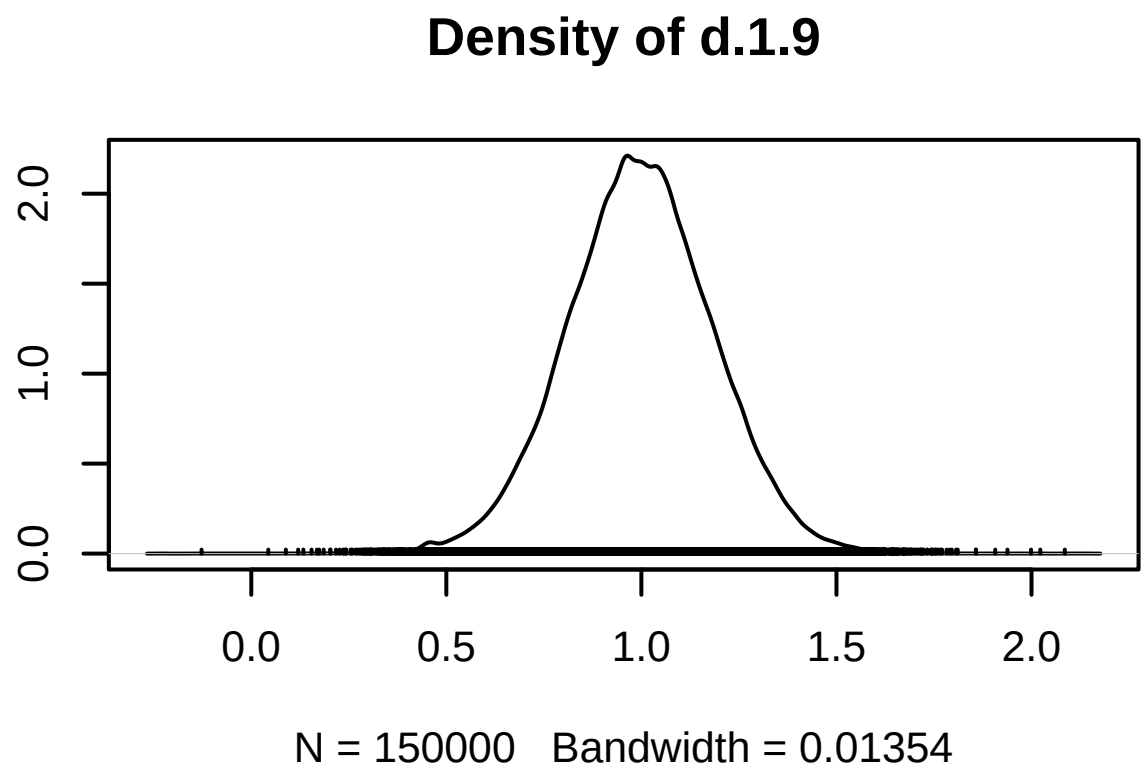
Density of d.1.9
0.0
1.0
2.0
0.0
0.5
1.0
1.5
2.0
N = 150000 Bandwidth = 0.01354

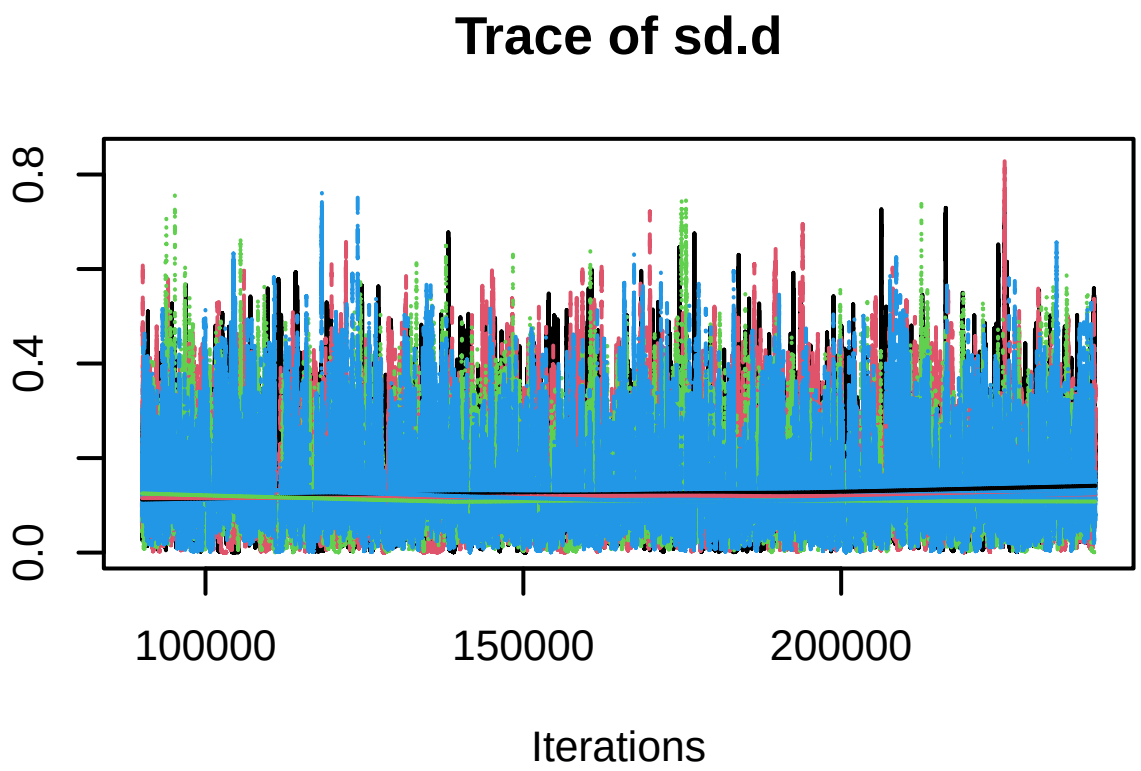
Trace of sd.d
0.0
0.4
0.8
100000
150000
200000
Iterations

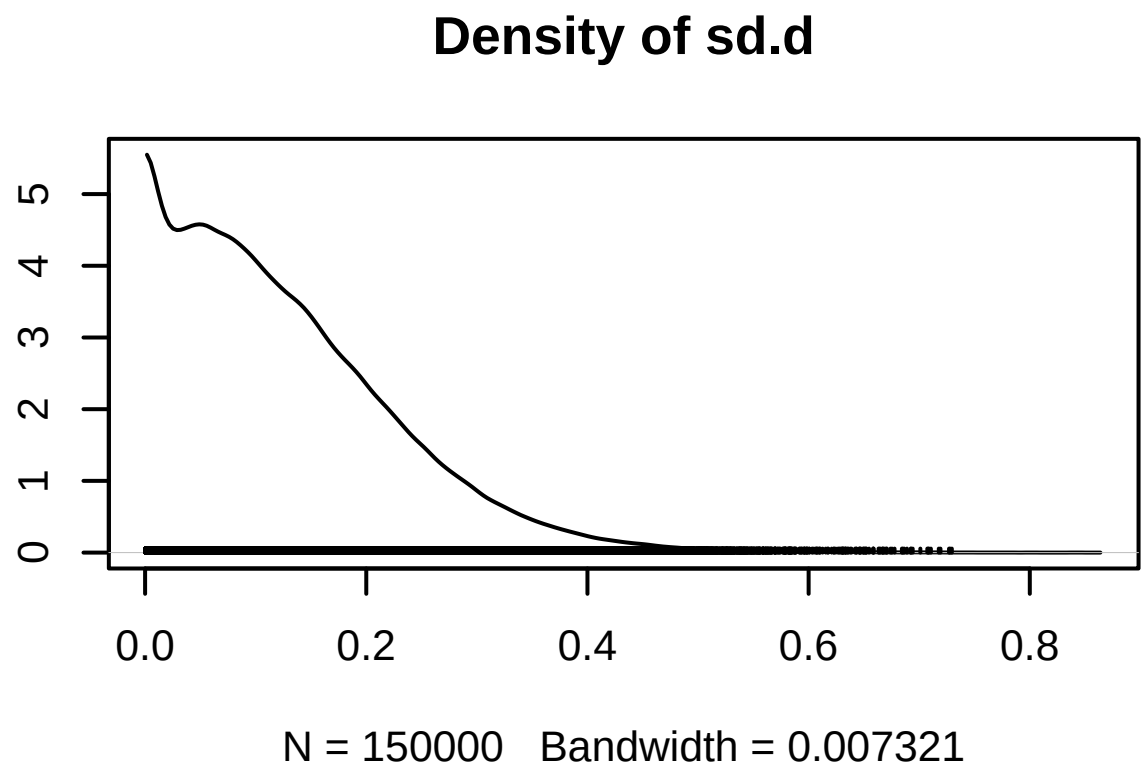
Density of sd.d
0
1
2
3
4
5
0.0
0.2
0.4
0.6
0.8
N = 150000 Bandwidth = 0.007321
