# Supplementary material for: Comparative safety of interleukin inhibitors for rheumatoid arthritis: a systematic review and network meta-analysis of randomized controlled trials
Source: Front Immunol. 2026 Sep 23;17:1866093. doi: 10.3389/fimmu.2026.1866093 (PMC13642689; doi:10.3389/fimmu.2026.1866093)
Supplement: Supplementary file 7 [file DataSheet15.pdf]

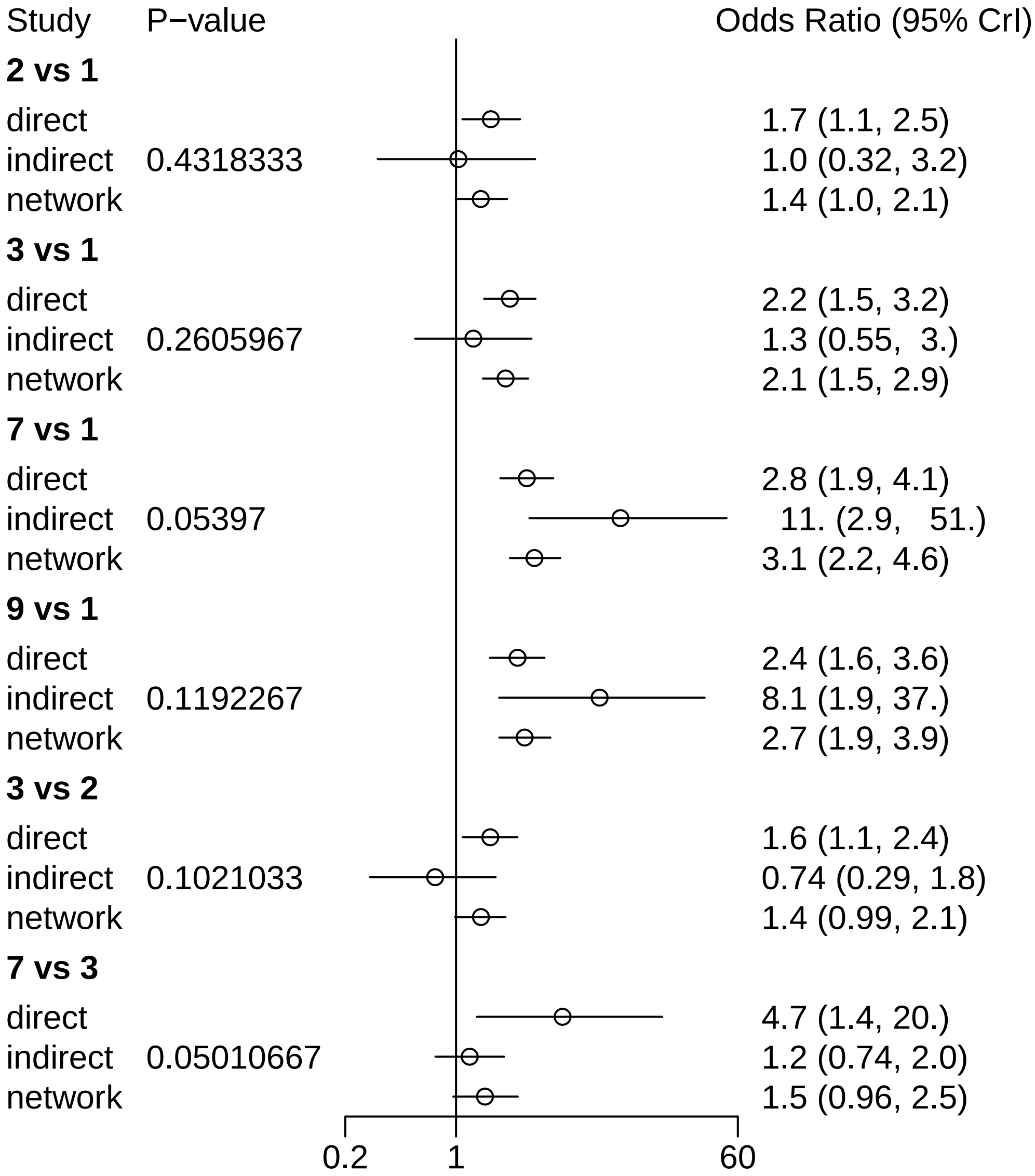

Study
P−value
Odds Ratio (95% CrI)
2 vs 1
direct
1.7 (1.1, 2.5)
indirect
0.4318333
1.0 (0.32, 3.2)
network
1.4 (1.0, 2.1)
3 vs 1
direct
2.2 (1.5, 3.2)
indirect
0.2605967
1.3 (0.55, 3.)
network
2.1 (1.5, 2.9)
7 vs 1
direct
2.8 (1.9, 4.1)
indirect
0.05397
11. (2.9, 51.)
network
3.1 (2.2, 4.6)
9 vs 1
direct
2.4 (1.6, 3.6)
indirect
0.1192267
8.1 (1.9, 37.)
network
2.7 (1.9, 3.9)
3 vs 2
direct
1.6 (1.1, 2.4)
indirect
0.1021033
0.74 (0.29, 1.8)
network
1.4 (0.99, 2.1)
7 vs 3
direct
4.7 (1.4, 20.)
indirect
0.05010667
1.2 (0.74, 2.0)
network
1.5 (0.96, 2.5)
0.2
1
60

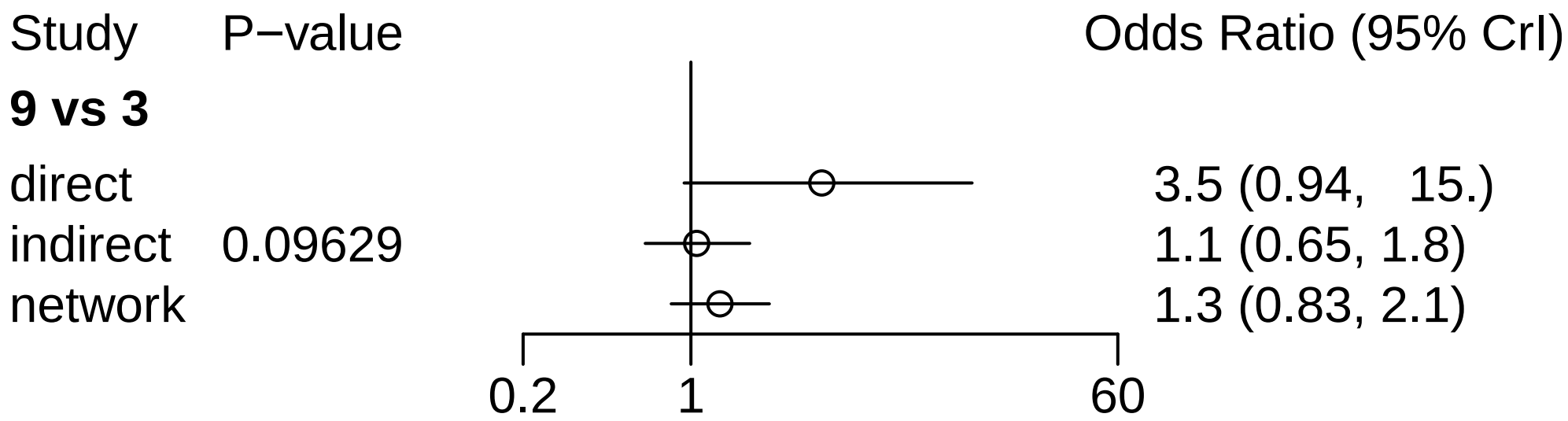
Study
P−value
Odds Ratio (95% CrI)
9 vs 3
direct
3.5 (0.94, 15.)
indirect
0.09629
1.1 (0.65, 1.8)
network
1.3 (0.83, 2.1)
0.2
1
60
