# Supplementary material for: Comparative safety of interleukin inhibitors for rheumatoid arthritis: a systematic review and network meta-analysis of randomized controlled trials
Source: Front Immunol. 2026 Sep 23;17:1866093. doi: 10.3389/fimmu.2026.1866093 (PMC13642689; doi:10.3389/fimmu.2026.1866093)
Supplement: Supplementary file 8 [file DataSheet2.pdf]

Trace of d.1.10

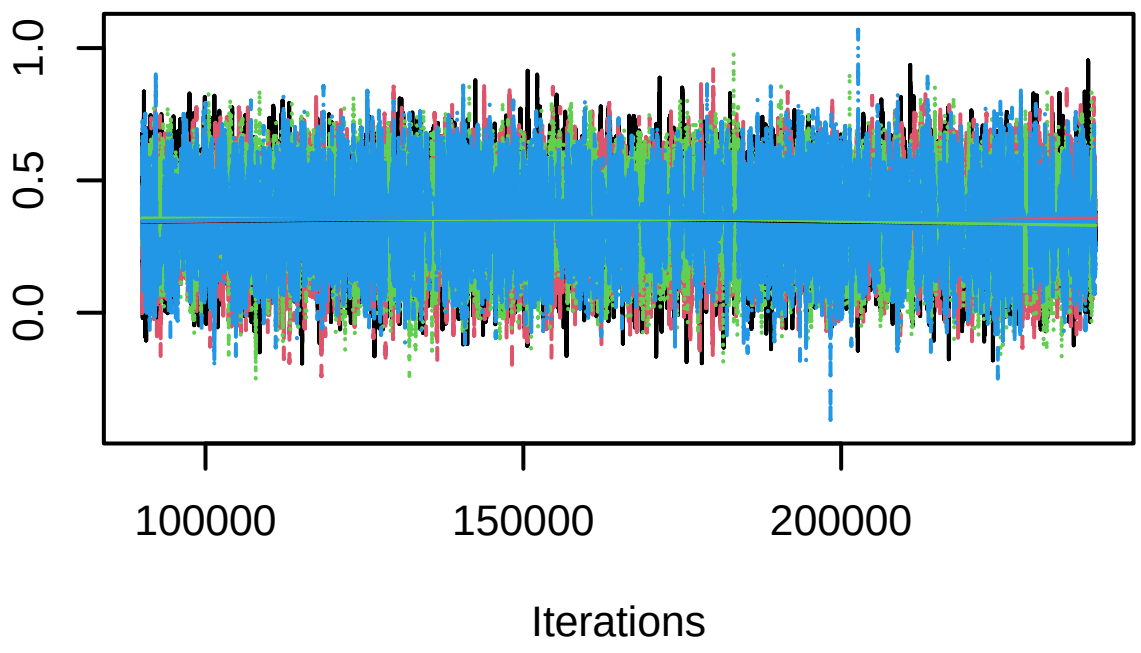

Density of d.1.10

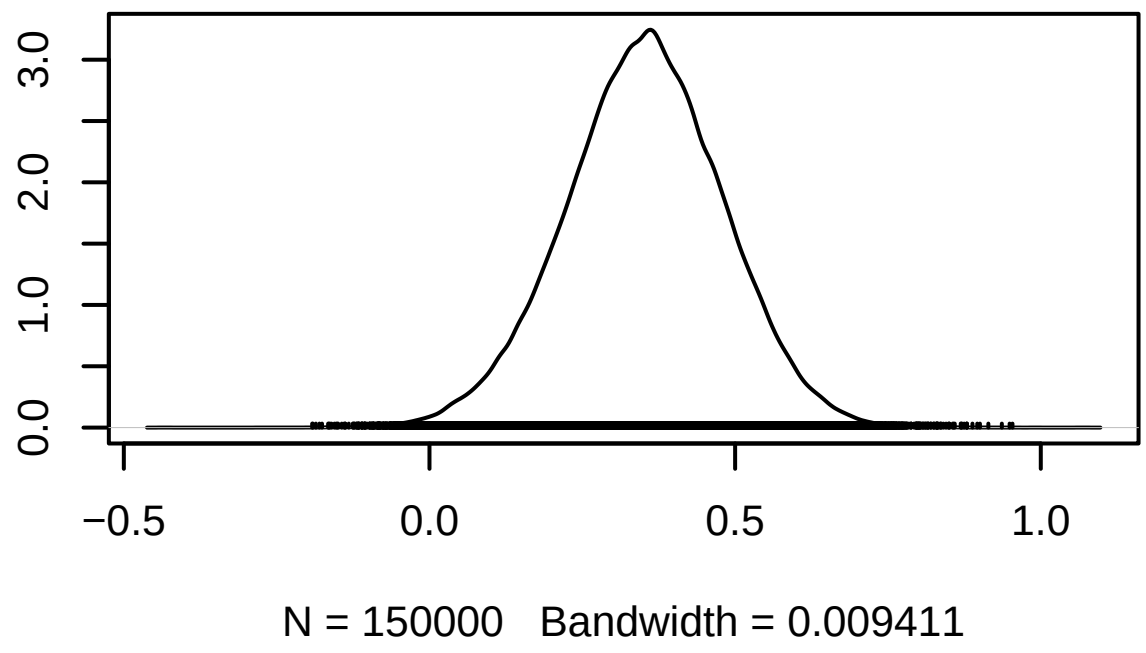

Trace of d.1.2

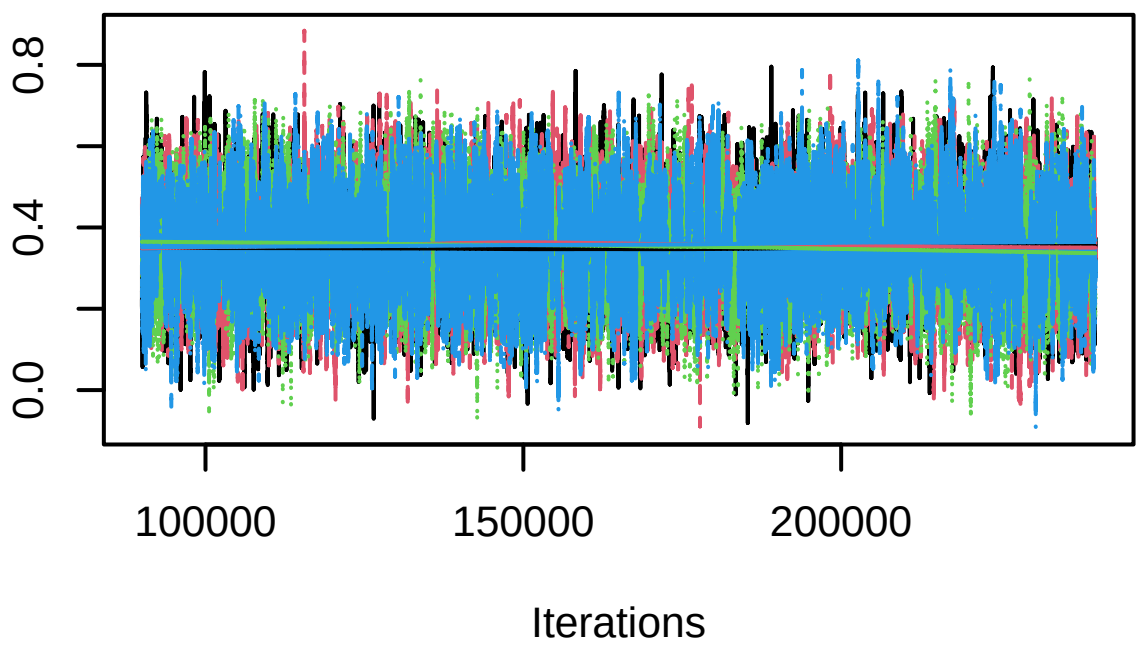

Density of d.1.2

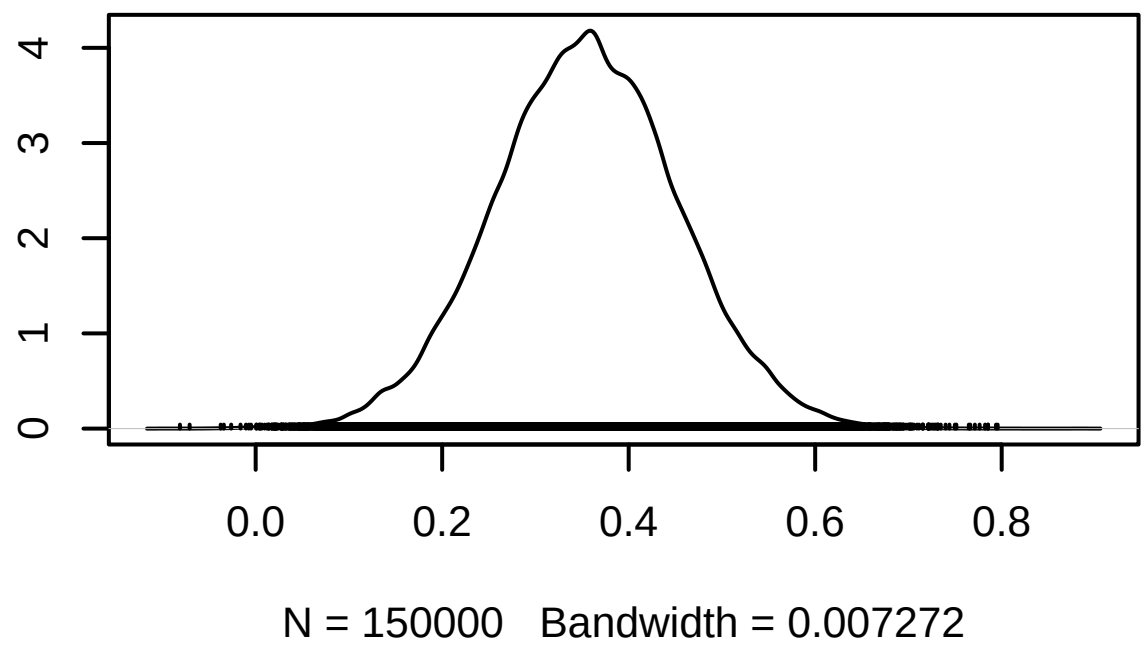

Trace of d.1.3

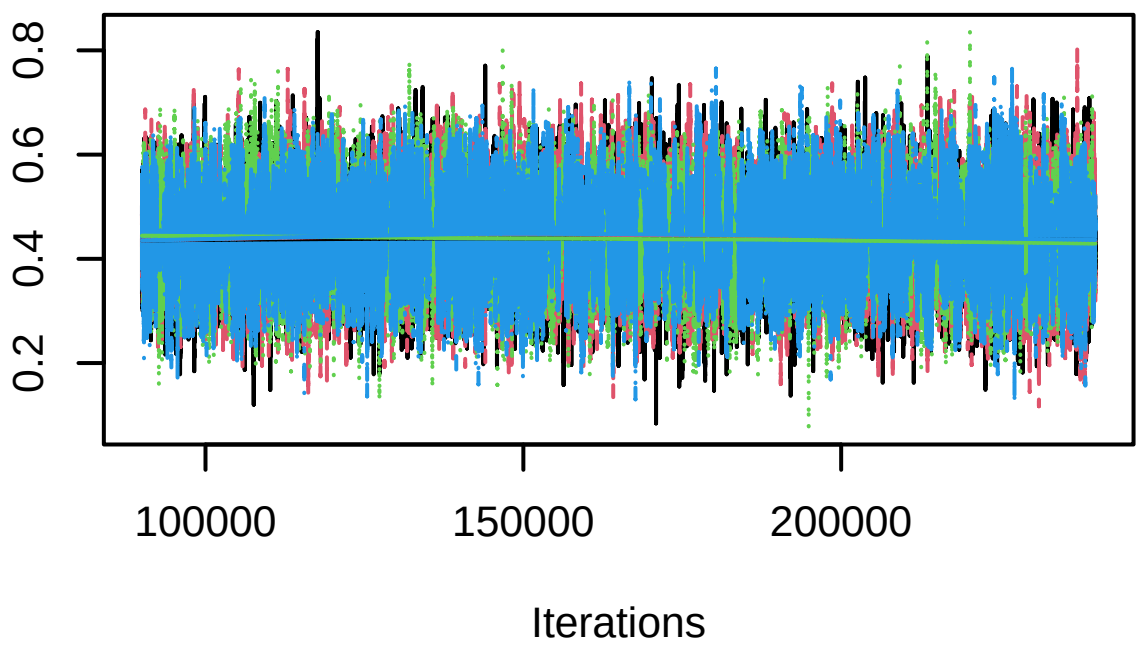

Density of d.1.3

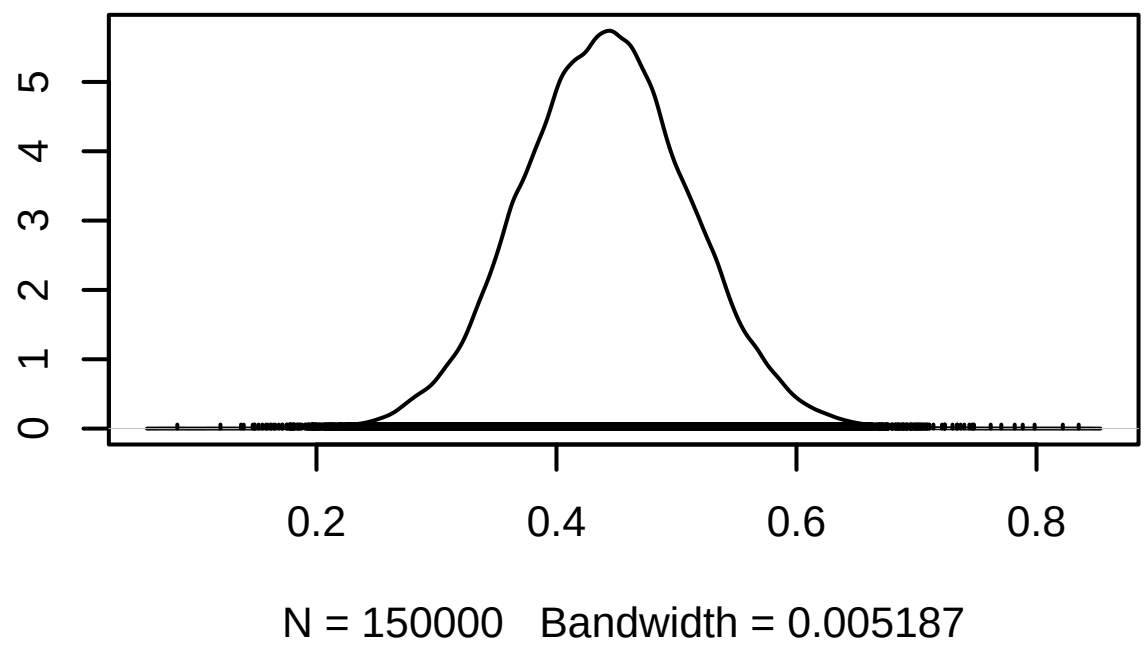

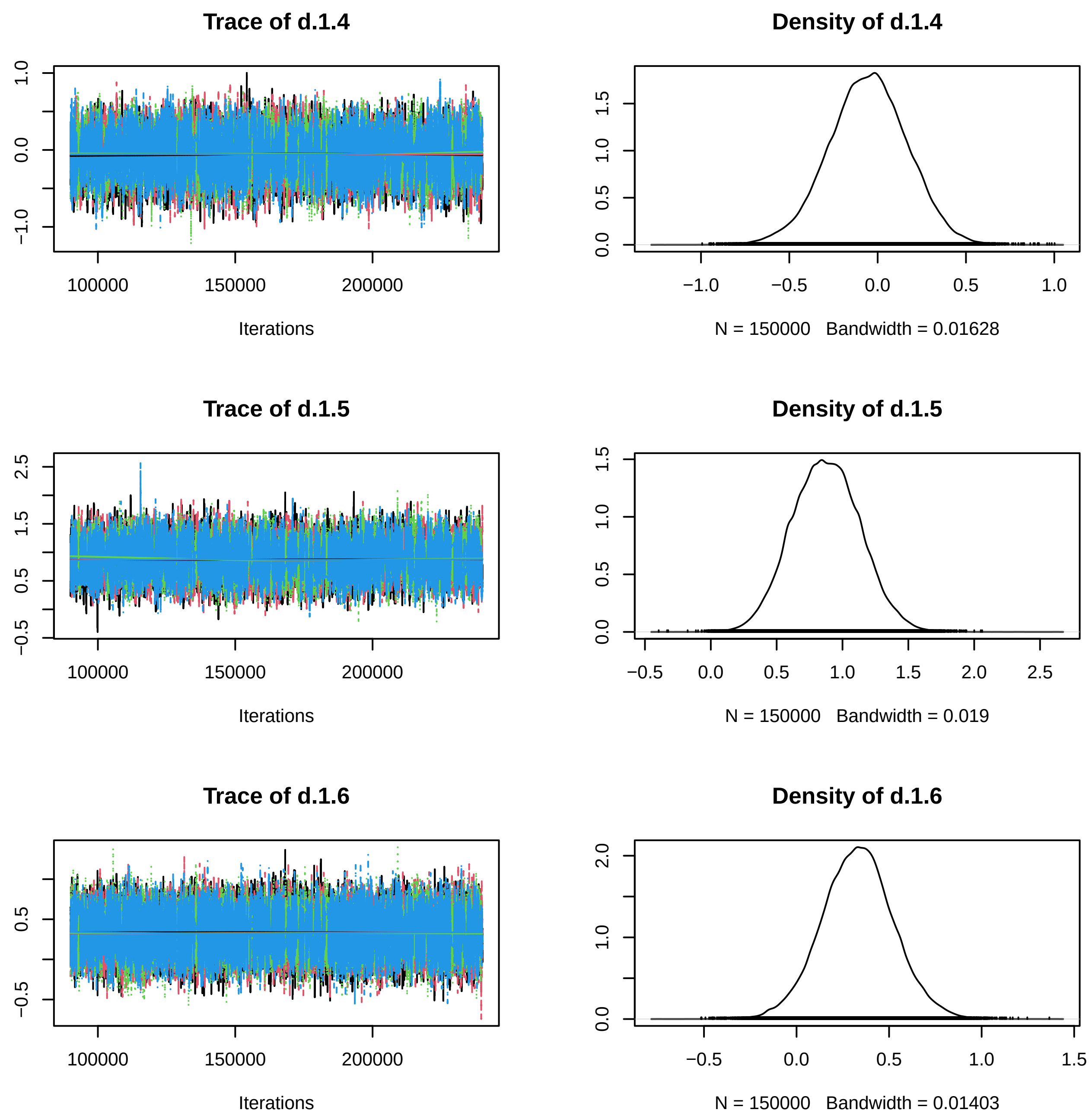

Trace of d.1.4
Iterations
Density of d.1.4
N = 150000 Bandwidth = 0.01628
Trace of d.1.5
Iterations
Density of d.1.5
N = 150000 Bandwidth = 0.019
Trace of d.1.6
Iterations
Density of d.1.6
N = 150000 Bandwidth = 0.01403

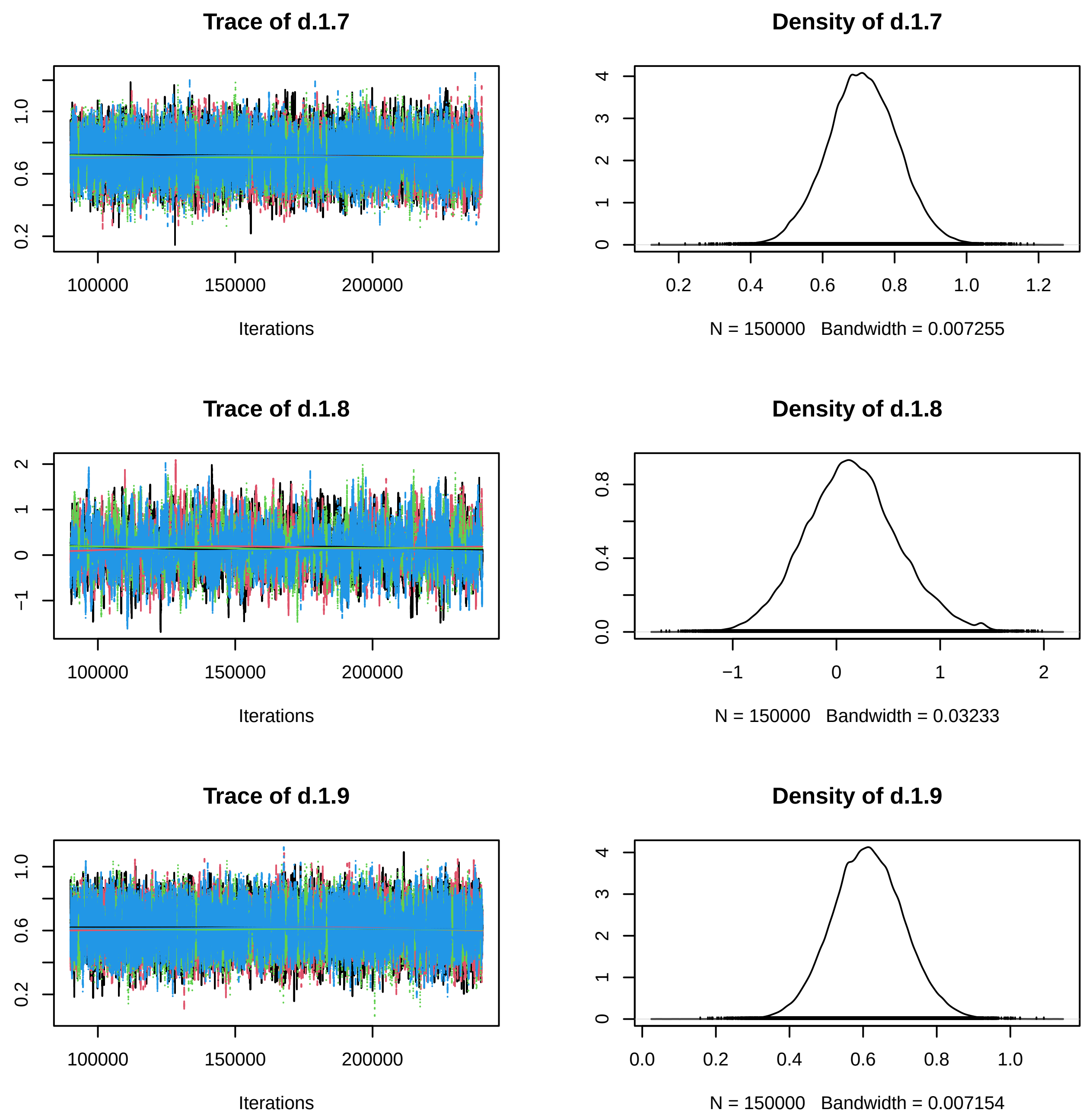
Trace of d.1.7
Iterations
Density of d.1.7
N = 150000 Bandwidth = 0.007255
Trace of d.1.8
Iterations
Density of d.1.8
N = 150000 Bandwidth = 0.03233
Trace of d.1.9
Iterations
Density of d.1.9
N = 150000 Bandwidth = 0.007154

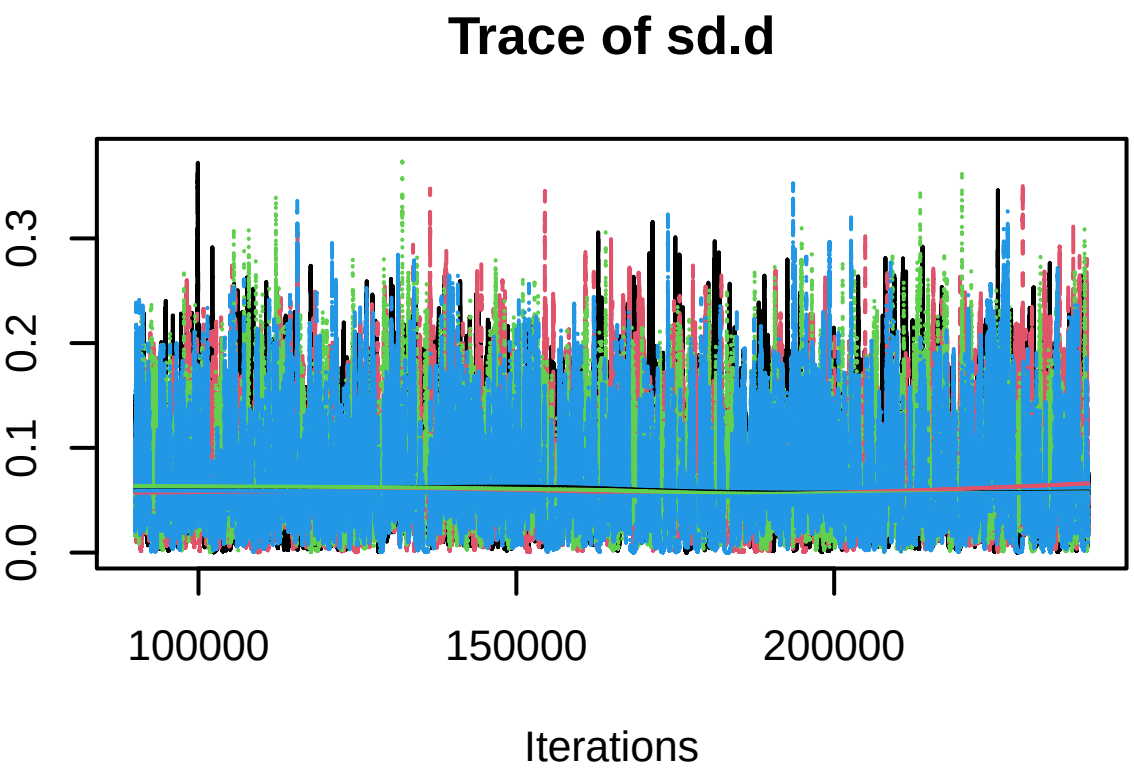
Trace of sd.d
0.0
0.1
0.2
0.3
100000
150000
200000
Iterations

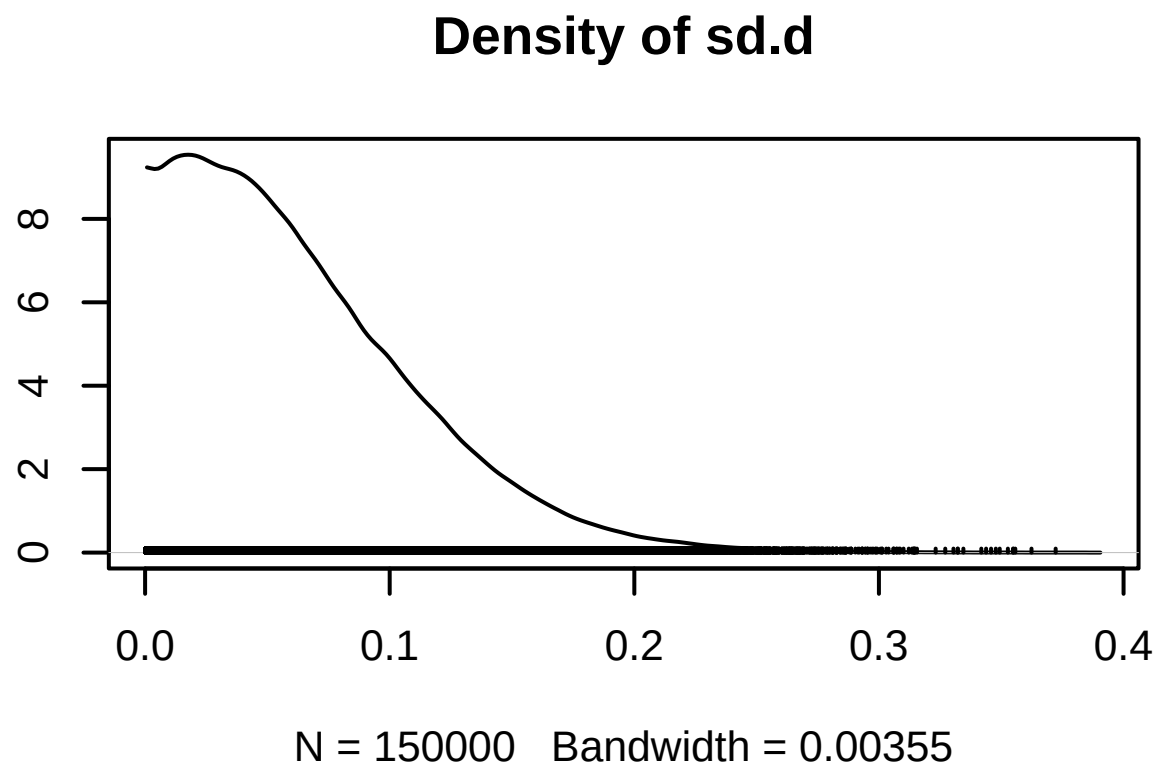
Density of sd.d
0
2
4
6
8
0.0
0.1
0.2
0.3
0.4
N = 150000 Bandwidth = 0.00355
