# Supplementary material for: Comparative safety of interleukin inhibitors for rheumatoid arthritis: a systematic review and network meta-analysis of randomized controlled trials
Source: Front Immunol. 2026 Sep 23;17:1866093. doi: 10.3389/fimmu.2026.1866093 (PMC13642689; doi:10.3389/fimmu.2026.1866093)
Supplement: Supplementary file 9 [file DataSheet3.pdf]

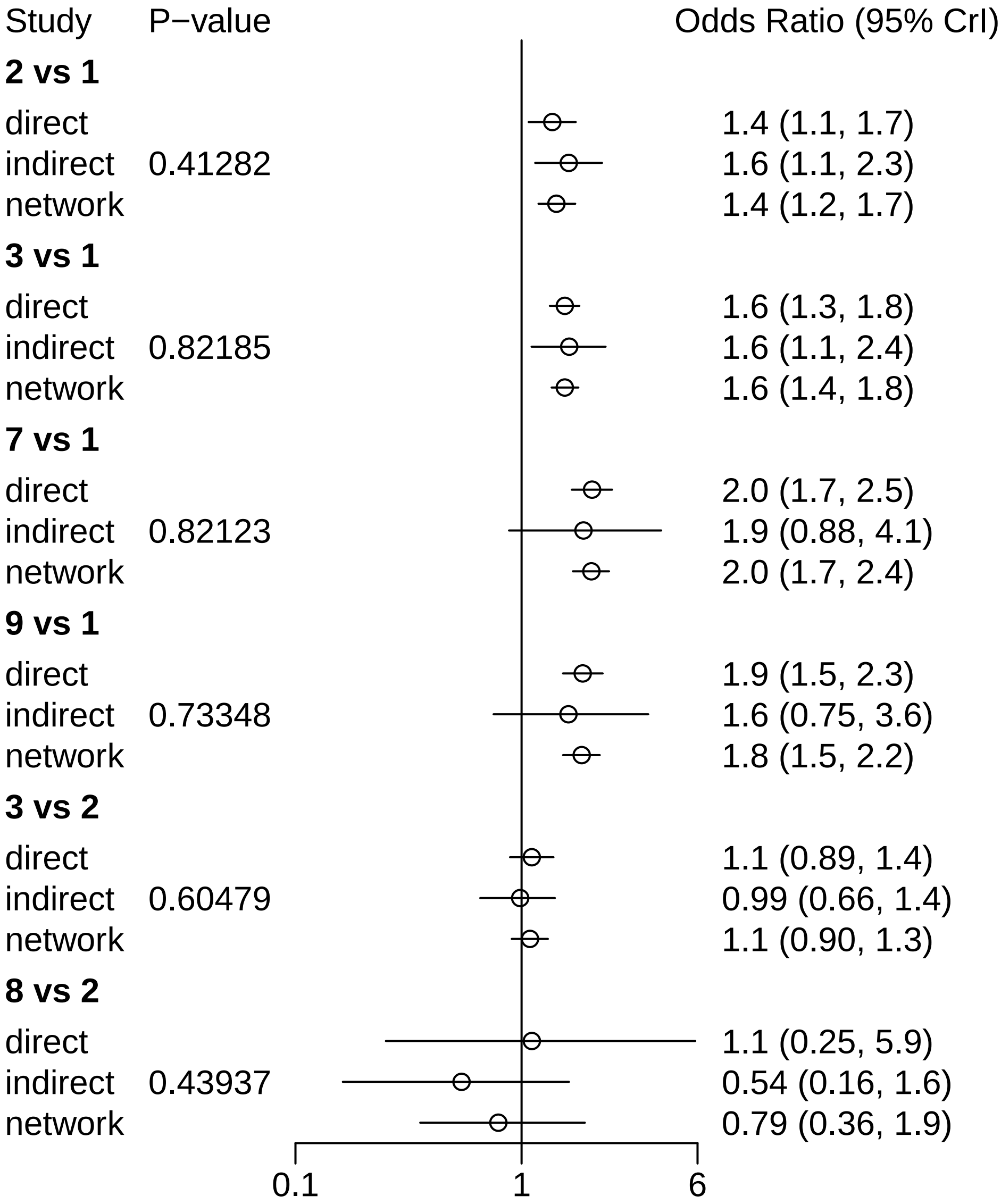

Study
P−value
Odds Ratio (95% CrI)
2 vs 1
direct
1.4 (1.1, 1.7)
indirect
0.41282
1.6 (1.1, 2.3)
network
1.4 (1.2, 1.7)
3 vs 1
direct
1.6 (1.3, 1.8)
indirect
0.82185
1.6 (1.1, 2.4)
network
1.6 (1.4, 1.8)
7 vs 1
direct
2.0 (1.7, 2.5)
indirect
0.82123
1.9 (0.88, 4.1)
network
2.0 (1.7, 2.4)
9 vs 1
direct
1.9 (1.5, 2.3)
indirect
0.73348
1.6 (0.75, 3.6)
network
1.8 (1.5, 2.2)
3 vs 2
direct
1.1 (0.89, 1.4)
indirect
0.60479
0.99 (0.66, 1.4)
network
1.1 (0.90, 1.3)
8 vs 2
direct
1.1 (0.25, 5.9)
indirect
0.43937
0.54 (0.16, 1.6)
network
0.79 (0.36, 1.9)
0.1
1
6

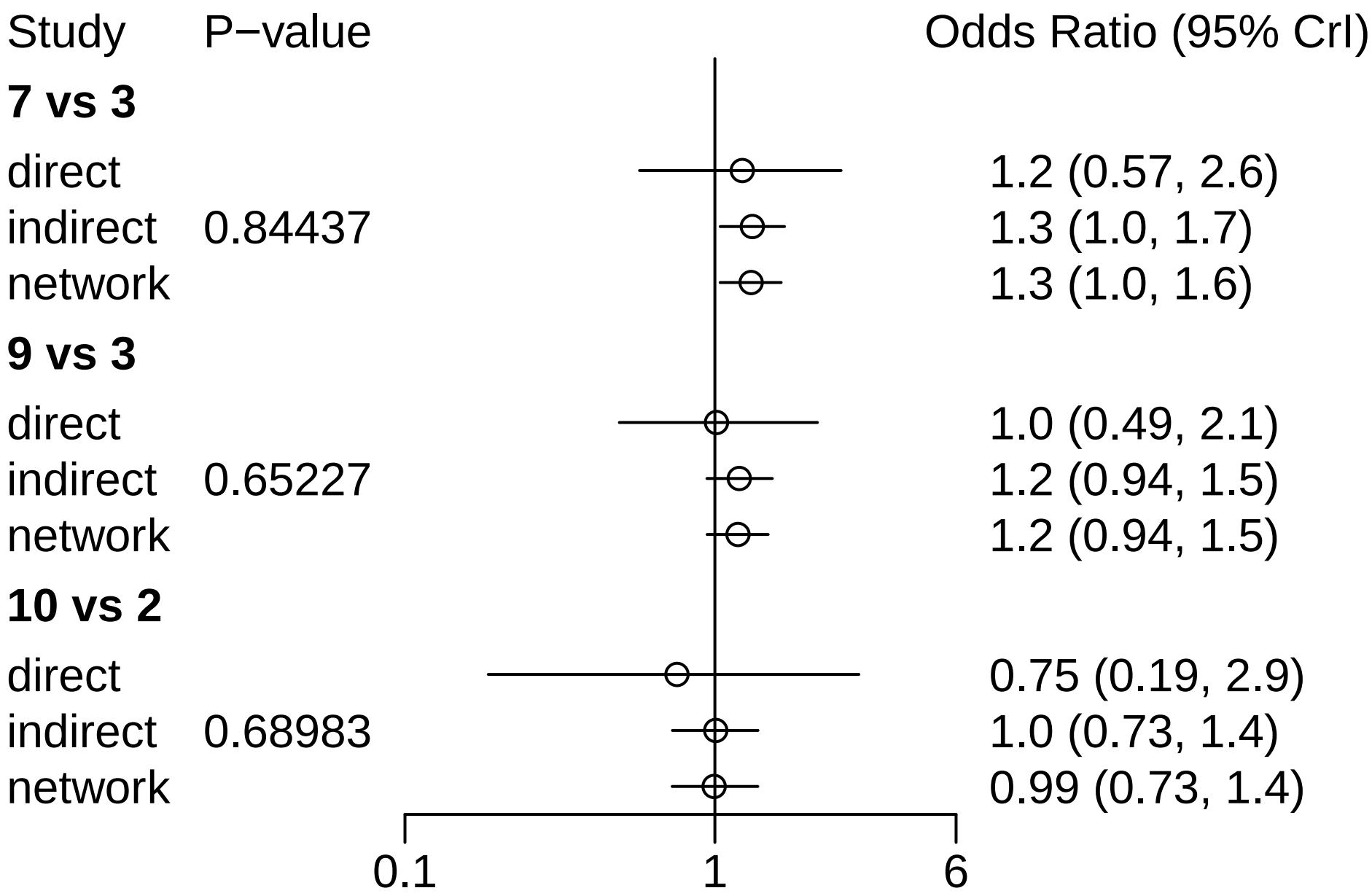

Study
P−value
Odds Ratio (95% CrI)
7 vs 3
direct
1.2 (0.57, 2.6)
indirect
0.84437
1.3 (1.0, 1.7)
network
1.3 (1.0, 1.6)
9 vs 3
direct
1.0 (0.49, 2.1)
indirect
0.65227
1.2 (0.94, 1.5)
network
1.2 (0.94, 1.5)
10 vs 2
direct
0.75 (0.19, 2.9)
indirect
0.68983
1.0 (0.73, 1.4)
network
0.99 (0.73, 1.4)
0.1
1
6
