# Supplementary material for: Comparative safety of interleukin inhibitors for rheumatoid arthritis: a systematic review and network meta-analysis of randomized controlled trials
Source: Front Immunol. 2026 Sep 23;17:1866093. doi: 10.3389/fimmu.2026.1866093 (PMC13642689; doi:10.3389/fimmu.2026.1866093)
Supplement: Supplementary file 11 [file DataSheet5.pdf]

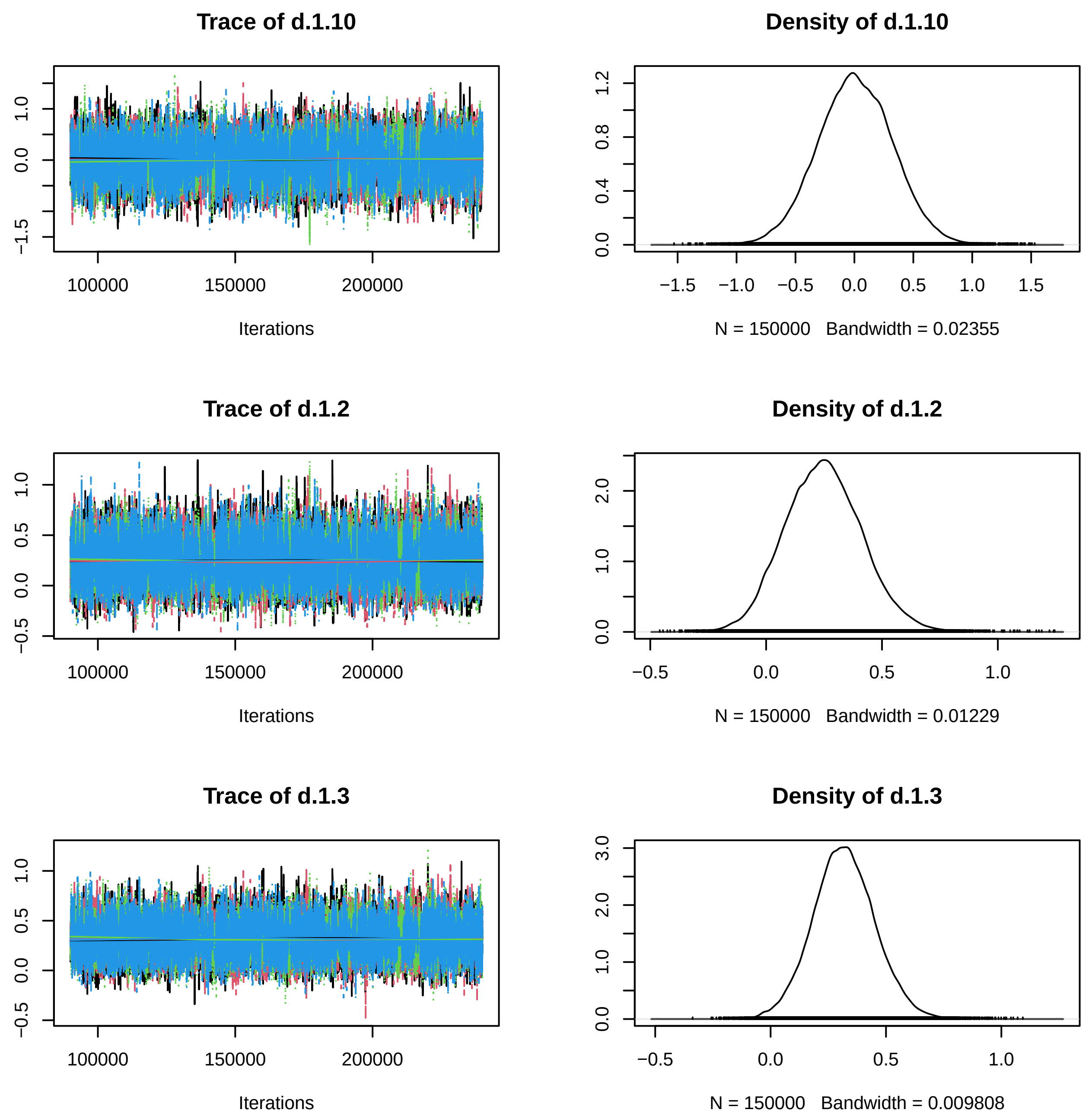
Trace of d.1.10
Iterations
Density of d.1.10
N = 150000 Bandwidth = 0.02355
Trace of d.1.2
Iterations
Density of d.1.2
N = 150000 Bandwidth = 0.01229
Trace of d.1.3
Iterations
Density of d.1.3
N = 150000 Bandwidth = 0.009808

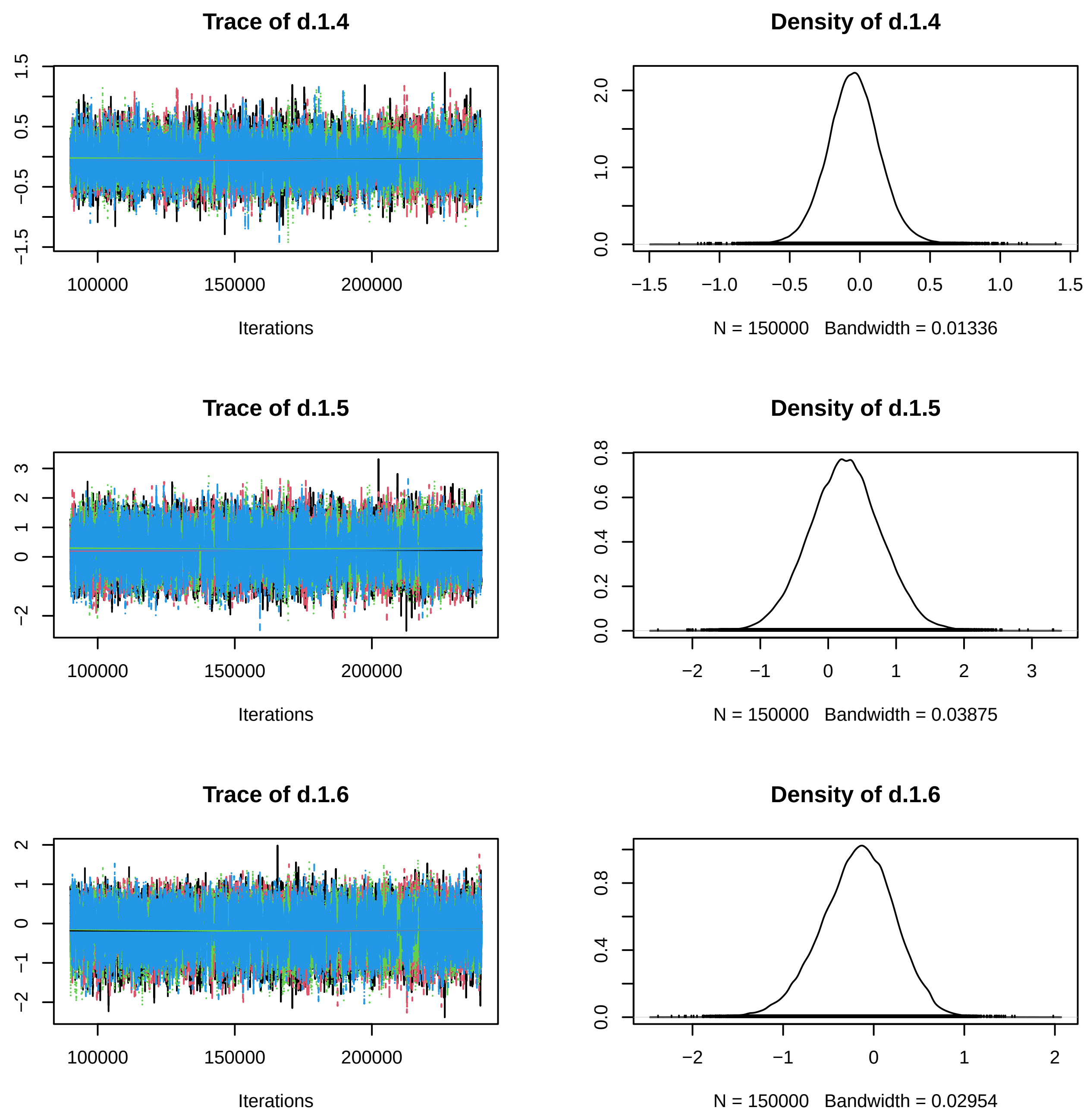
Trace of d.1.4
Iterations
Density of d.1.4
N = 150000 Bandwidth = 0.01336
Trace of d.1.5
Iterations
Density of d.1.5
N = 150000 Bandwidth = 0.03875
Trace of d.1.6
Iterations
Density of d.1.6
N = 150000 Bandwidth = 0.02954

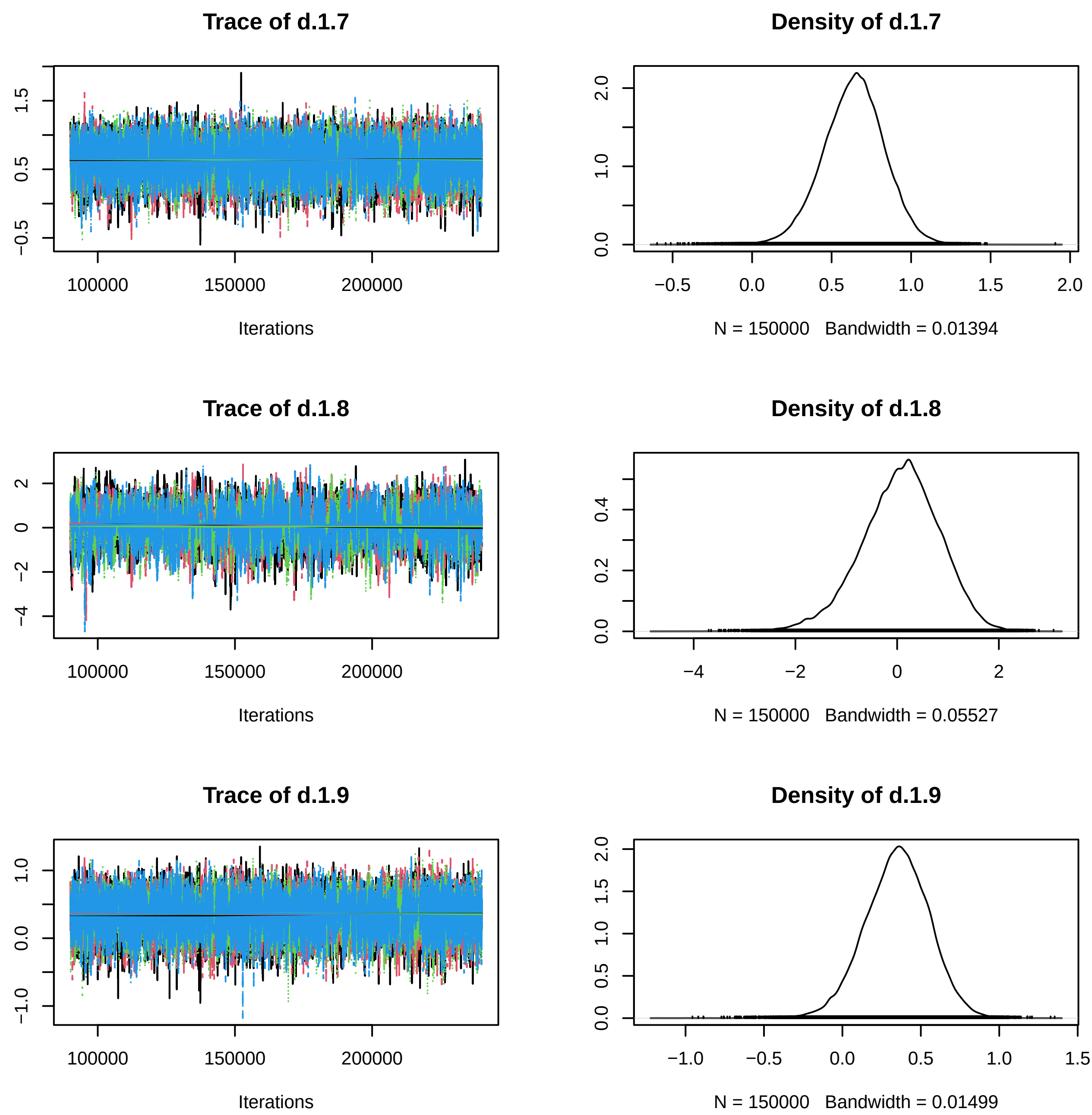
Trace of d.1.7
Iterations
Density of d.1.7
N = 150000 Bandwidth = 0.01394
Trace of d.1.8
Iterations
Density of d.1.8
N = 150000 Bandwidth = 0.05527
Trace of d.1.9
Iterations
Density of d.1.9
N = 150000 Bandwidth = 0.01499

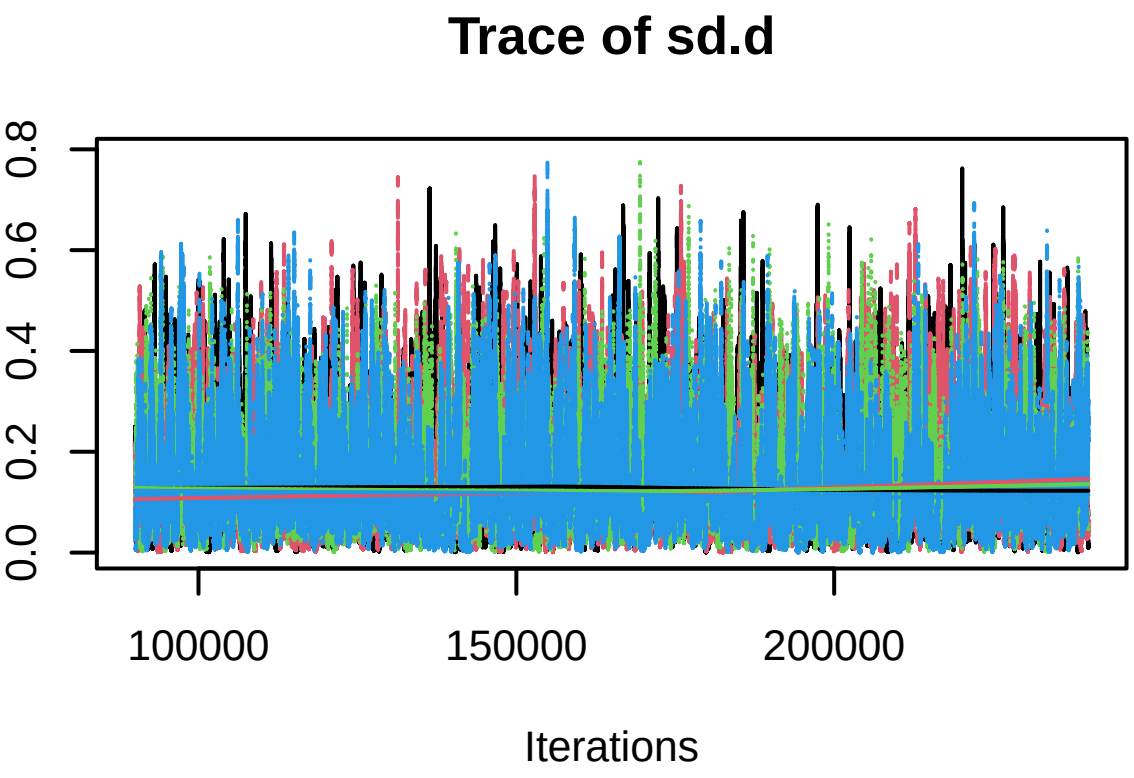
Trace of sd.d
0.0
0.2
0.4
0.6
0.8
100000
150000
200000
Iterations

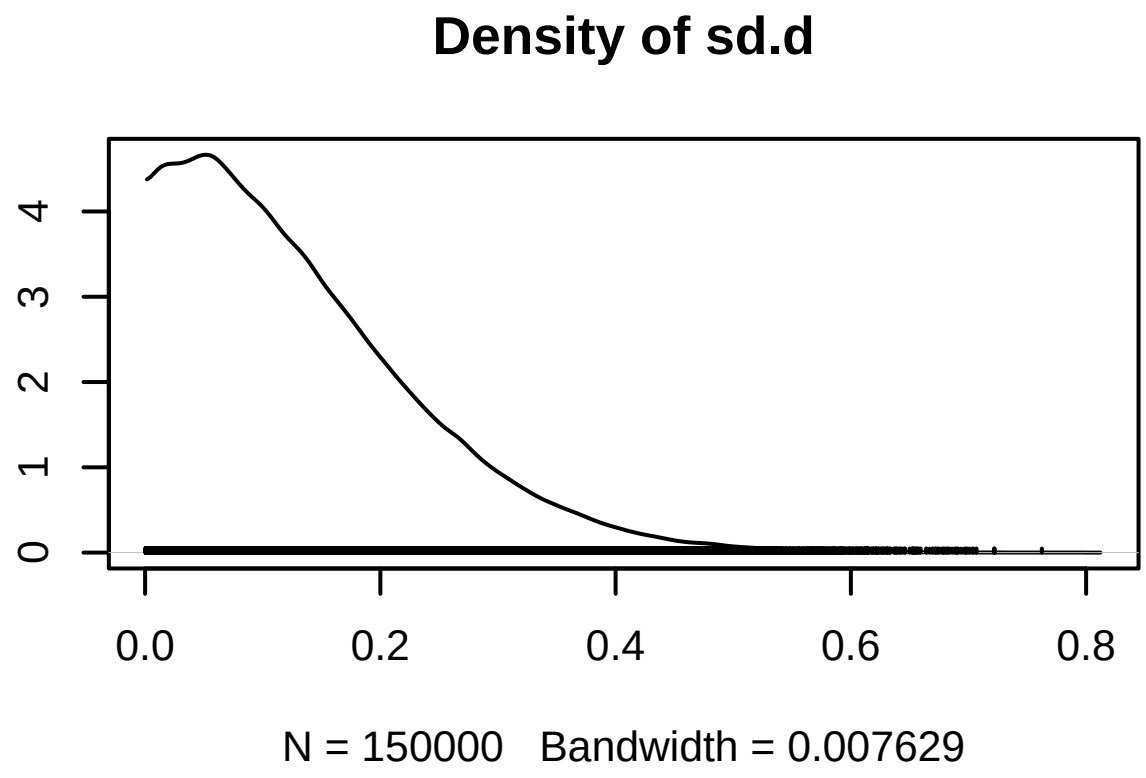
Density of sd.d
0
1
2
3
4
0.0
0.2
0.4
0.6
0.8
N = 150000 Bandwidth = 0.007629
