# Supplementary material for: Comparative safety of interleukin inhibitors for rheumatoid arthritis: a systematic review and network meta-analysis of randomized controlled trials
Source: Front Immunol. 2026 Sep 23;17:1866093. doi: 10.3389/fimmu.2026.1866093 (PMC13642689; doi:10.3389/fimmu.2026.1866093)
Supplement: Supplementary file 12 [file DataSheet6.pdf]

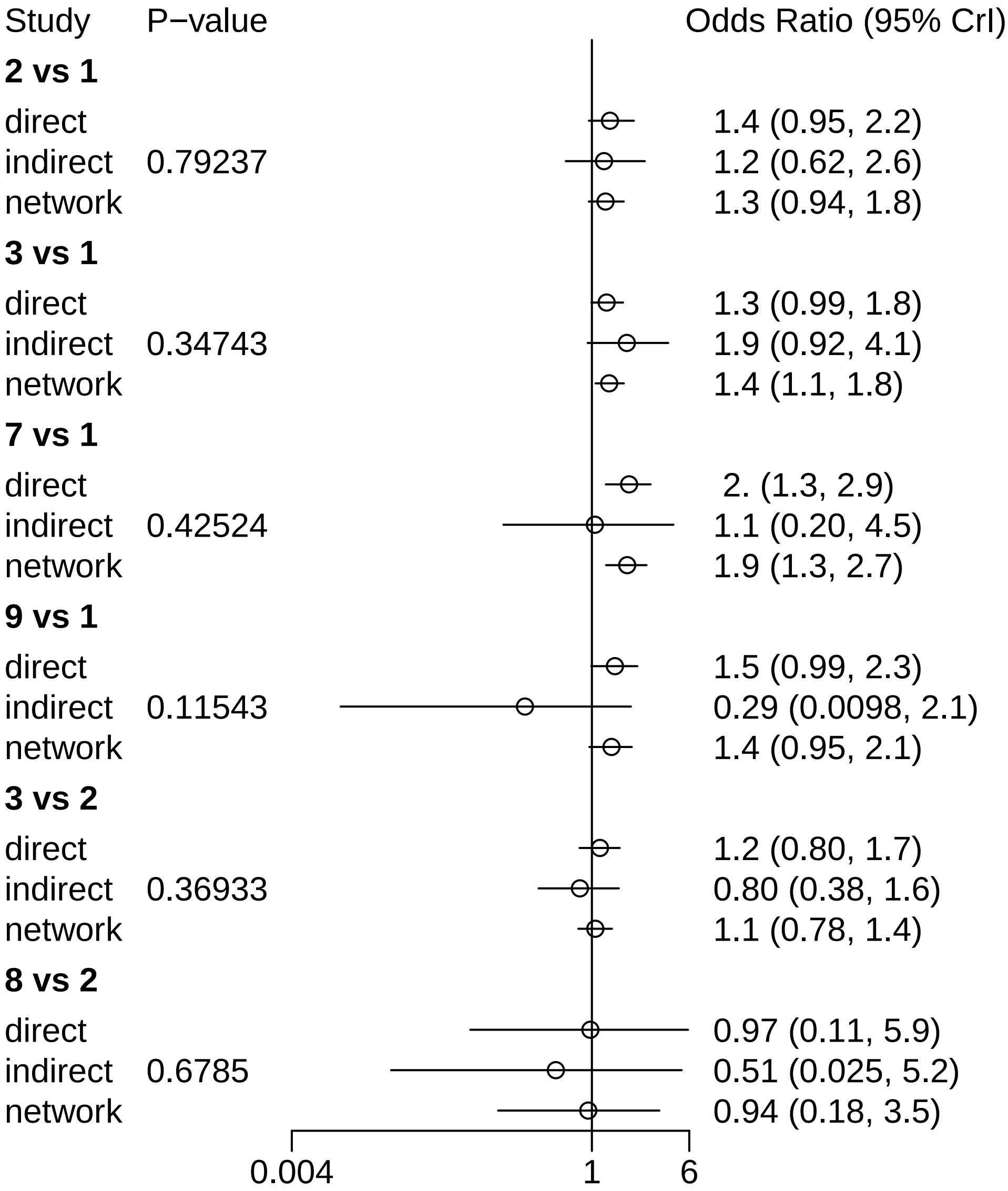
Study
P−value
Odds Ratio (95% CrI)
2 vs 1
direct 1.4 (0.95, 2.2)
indirect 0.79237 1.2 (0.62, 2.6)
network 1.3 (0.94, 1.8)
3 vs 1
direct 1.3 (0.99, 1.8)
indirect 0.34743 1.9 (0.92, 4.1)
network 1.4 (1.1, 1.8)
7 vs 1
direct 2. (1.3, 2.9)
indirect 0.42524 1.1 (0.20, 4.5)
network 1.9 (1.3, 2.7)
9 vs 1
direct 1.5 (0.99, 2.3)
indirect 0.11543 0.29 (0.0098, 2.1)
network 1.4 (0.95, 2.1)
3 vs 2
direct 1.2 (0.80, 1.7)
indirect 0.36933 0.80 (0.38, 1.6)
network 1.1 (0.78, 1.4)
8 vs 2
direct 0.97 (0.11, 5.9)
indirect 0.6785 0.51 (0.025, 5.2)
network 0.94 (0.18, 3.5)
0.004
1
6

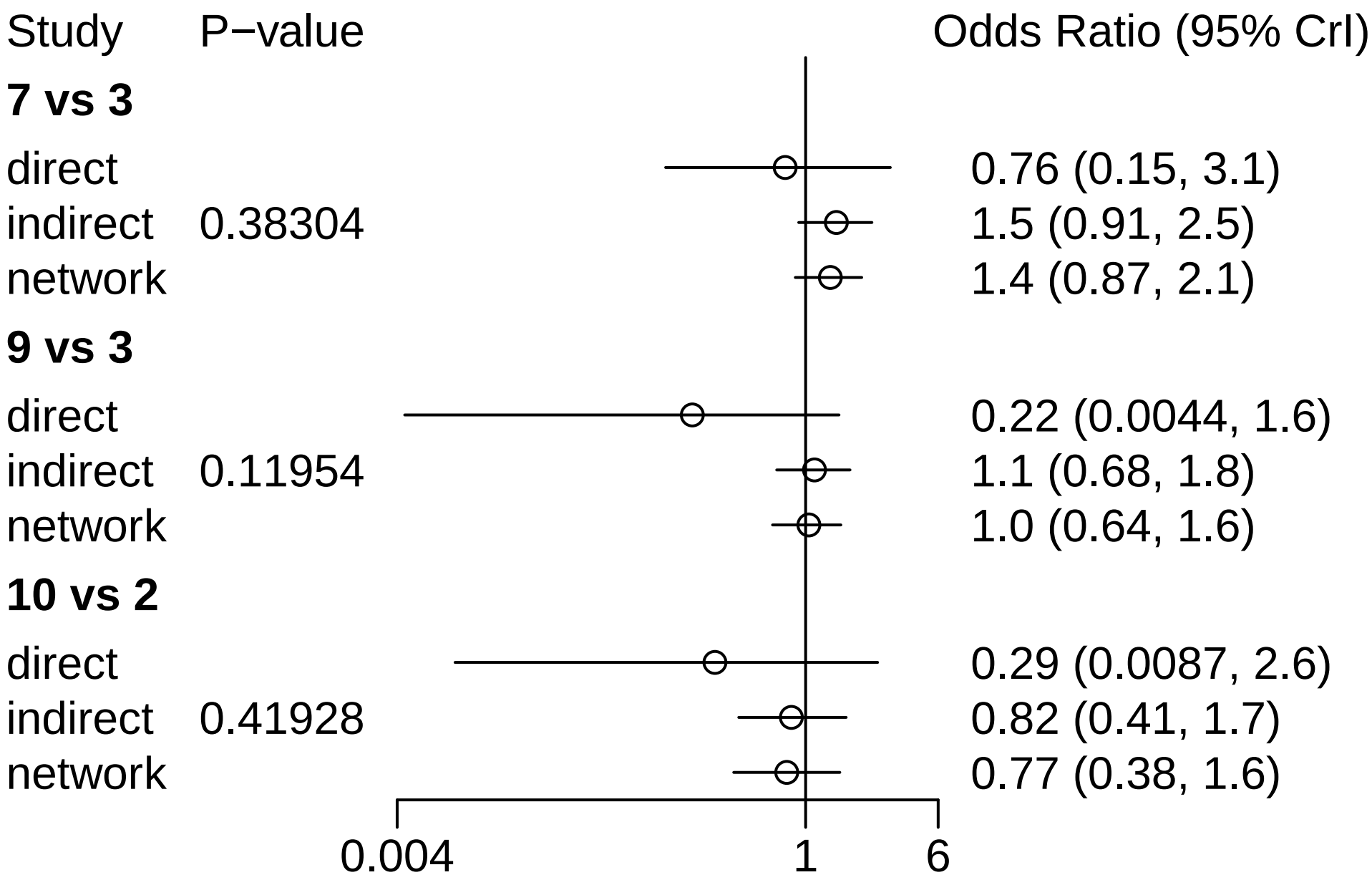
Study
P−value
Odds Ratio (95% CrI)
7 vs 3
direct
0.76 (0.15, 3.1)
indirect
0.38304
1.5 (0.91, 2.5)
network
1.4 (0.87, 2.1)
9 vs 3
direct
0.22 (0.0044, 1.6)
indirect
0.11954
1.1 (0.68, 1.8)
network
1.0 (0.64, 1.6)
10 vs 2
direct
0.29 (0.0087, 2.6)
indirect
0.41928
0.82 (0.41, 1.7)
network
0.77 (0.38, 1.6)
0.004
1
6
