# Supplementary material for: Comparative safety of interleukin inhibitors for rheumatoid arthritis: a systematic review and network meta-analysis of randomized controlled trials
Source: Front Immunol. 2026 Sep 23;17:1866093. doi: 10.3389/fimmu.2026.1866093 (PMC13642689; doi:10.3389/fimmu.2026.1866093)
Supplement: Supplementary file 13 [file DataSheet7.pdf]

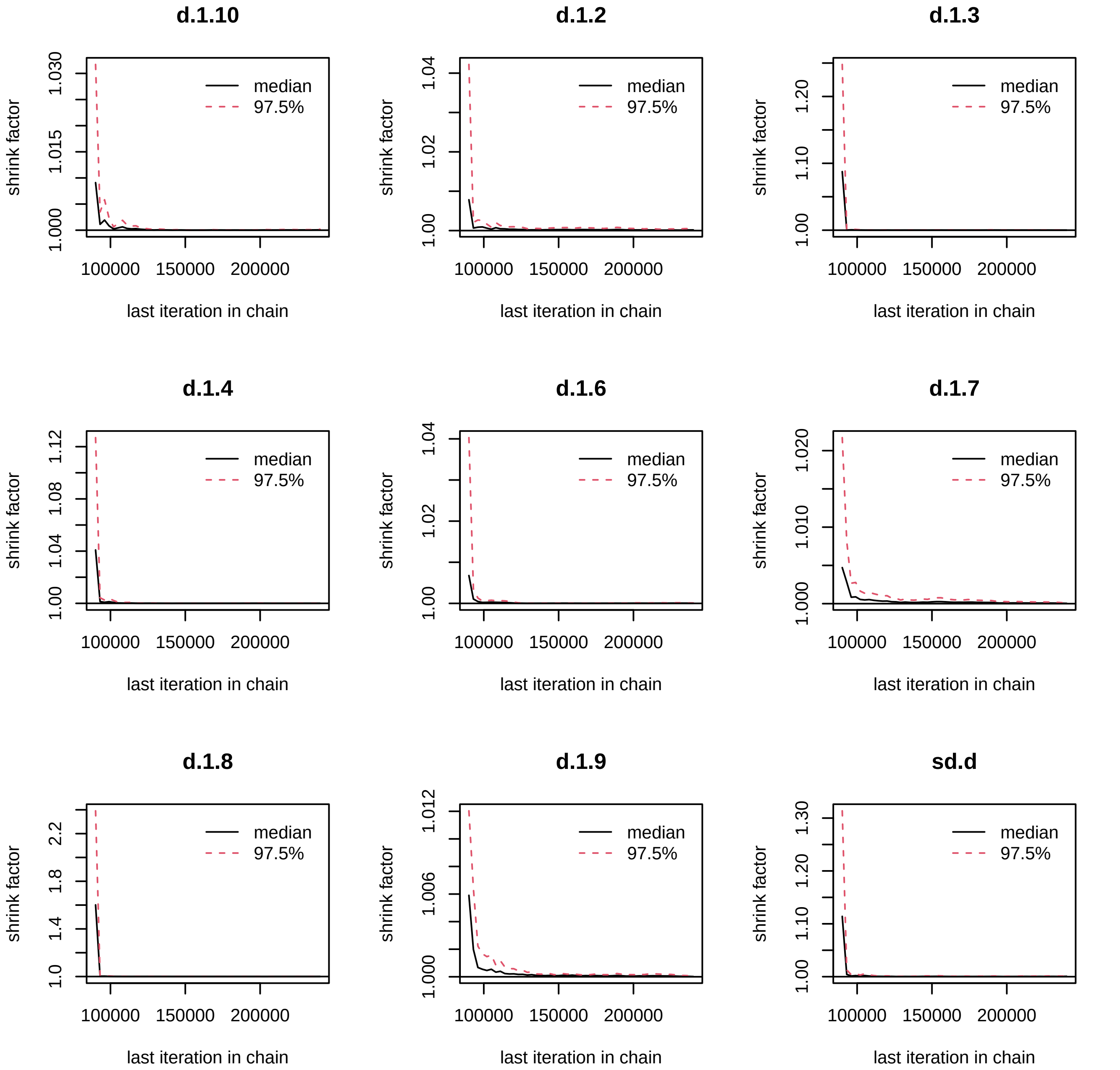
d.1.10
shrink factor
1.000
1.015
1.030
100000
150000
200000
last iteration in chain
median
97.5%
d.1.2
shrink factor
1.00
1.02
1.04
100000
150000
200000
last iteration in chain
median
97.5%
d.1.3
shrink factor
1.00
1.10
1.20
100000
150000
200000
last iteration in chain
median
97.5%
d.1.4
shrink factor
1.00
1.04
1.08
1.12
100000
150000
200000
last iteration in chain
median
97.5%
d.1.6
shrink factor
1.00
1.02
1.04
100000
150000
200000
last iteration in chain
median
97.5%
d.1.7
shrink factor
1.000
1.010
1.020
100000
150000
200000
last iteration in chain
median
97.5%
d.1.8
shrink factor
1.0
1.4
1.8
2.2
100000
150000
200000
last iteration in chain
median
97.5%
d.1.9
shrink factor
1.000
1.006
1.012
100000
150000
200000
last iteration in chain
median
97.5%
sd.d
shrink factor
1.00
1.10
1.20
1.30
100000
150000
200000
last iteration in chain
median
97.5%
