# Supplementary material for: Comparative safety of interleukin inhibitors for rheumatoid arthritis: a systematic review and network meta-analysis of randomized controlled trials
Source: Front Immunol. 2026 Sep 23;17:1866093. doi: 10.3389/fimmu.2026.1866093 (PMC13642689; doi:10.3389/fimmu.2026.1866093)
Supplement: Supplementary file 14 [file DataSheet8.pdf]

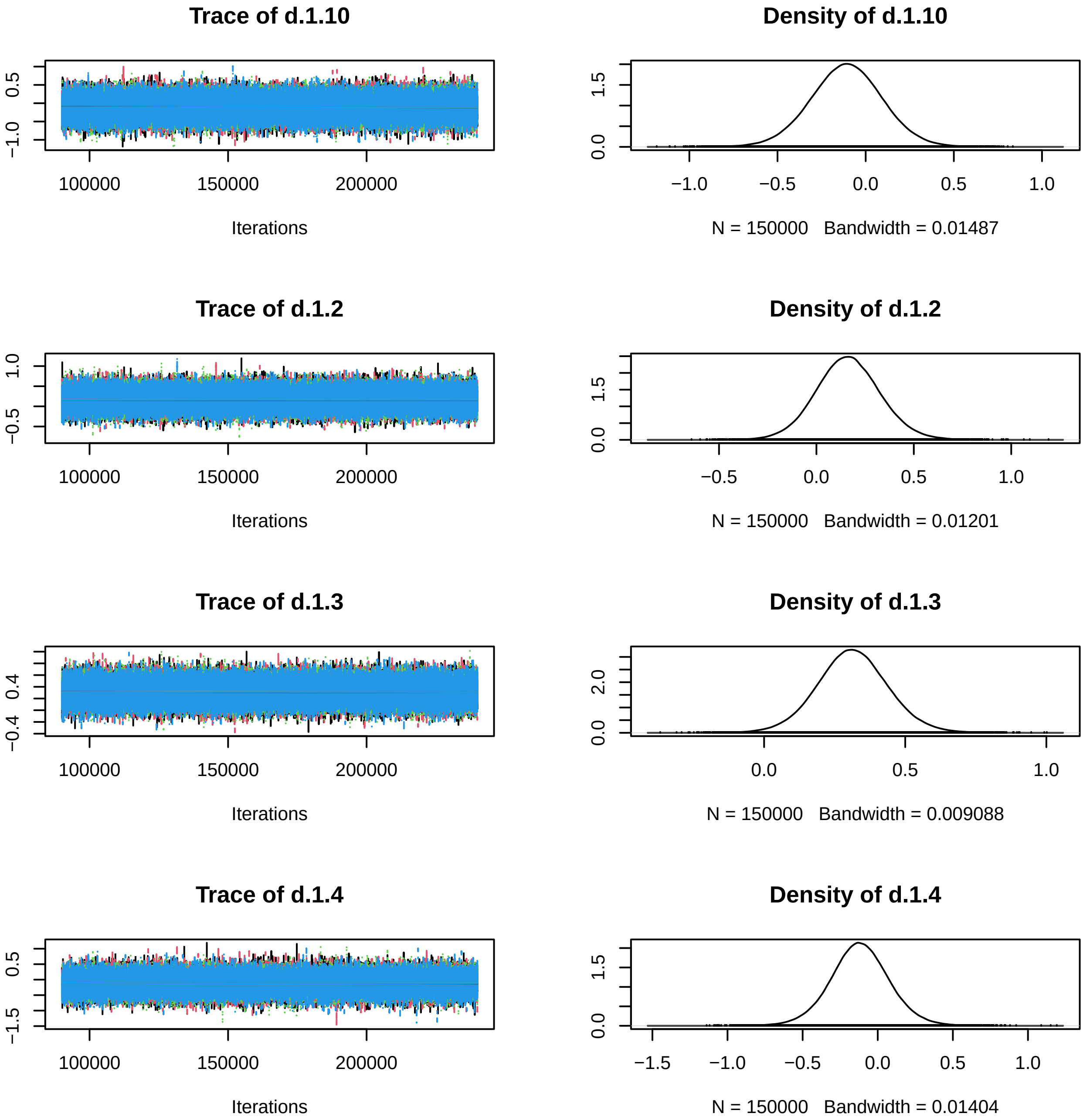
Trace of d.1.10
Density of d.1.10
0.5
−1.0
1.5
0.0
100000
150000
200000
−1.0
−0.5
0.0
0.5
1.0
Iterations
N = 150000 Bandwidth = 0.01487
Trace of d.1.2
Density of d.1.2
1.0
−0.5
1.5
0.0
100000
150000
200000
−0.5
0.0
0.5
1.0
Iterations
N = 150000 Bandwidth = 0.01201
Trace of d.1.3
Density of d.1.3
0.4
−0.4
2.0
0.0
100000
150000
200000
0.0
0.5
1.0
Iterations
N = 150000 Bandwidth = 0.009088
Trace of d.1.4
Density of d.1.4
0.5
−1.5
1.5
0.0
100000
150000
200000
−1.5
−1.0
−0.5
0.0
0.5
1.0
Iterations
N = 150000 Bandwidth = 0.01404

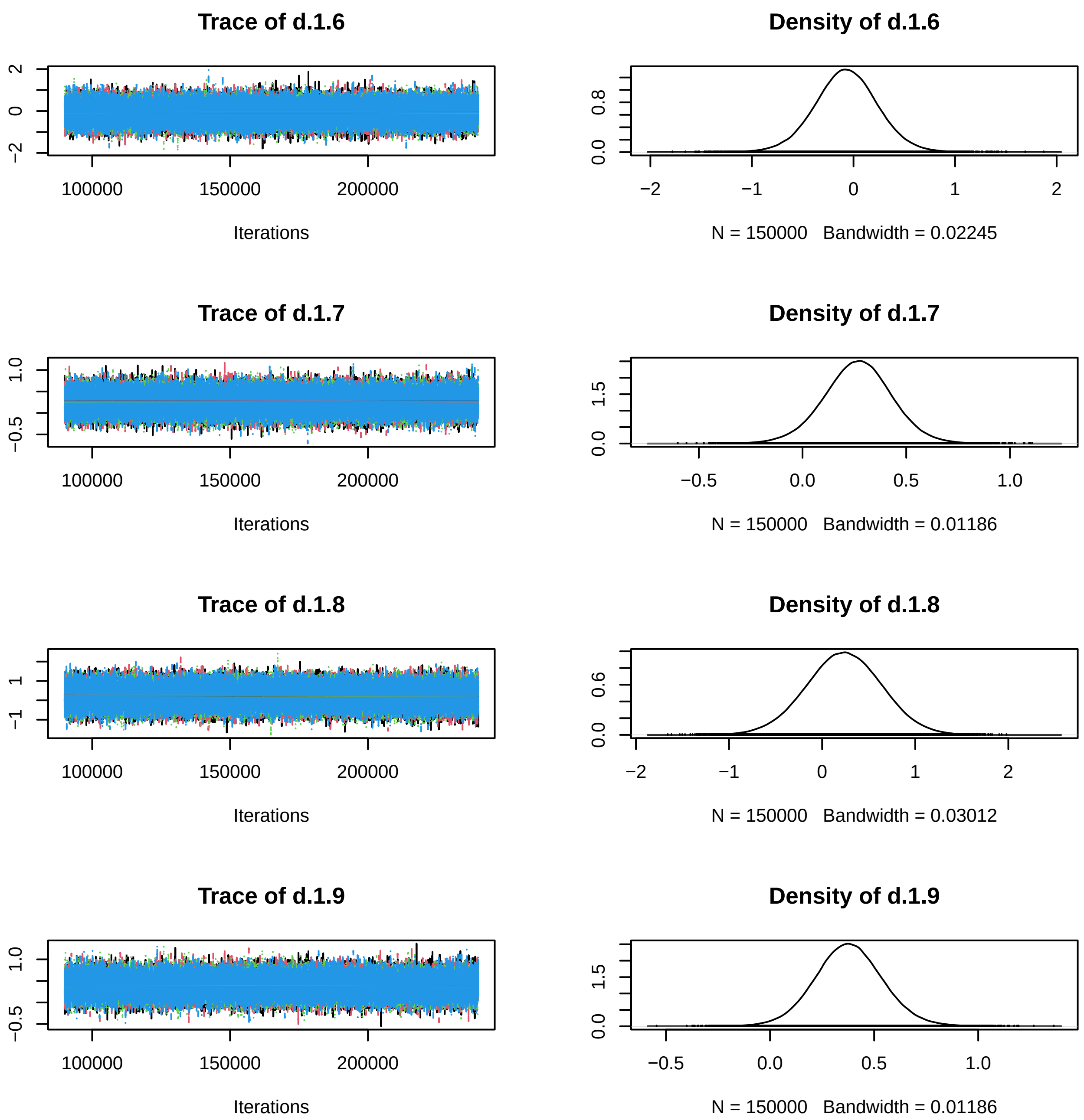
Trace of d.1.6
Iterations
Density of d.1.6
N = 150000 Bandwidth = 0.02245
Trace of d.1.7
Iterations
Density of d.1.7
N = 150000 Bandwidth = 0.01186
Trace of d.1.8
Iterations
Density of d.1.8
N = 150000 Bandwidth = 0.03012
Trace of d.1.9
Iterations
Density of d.1.9
N = 150000 Bandwidth = 0.01186

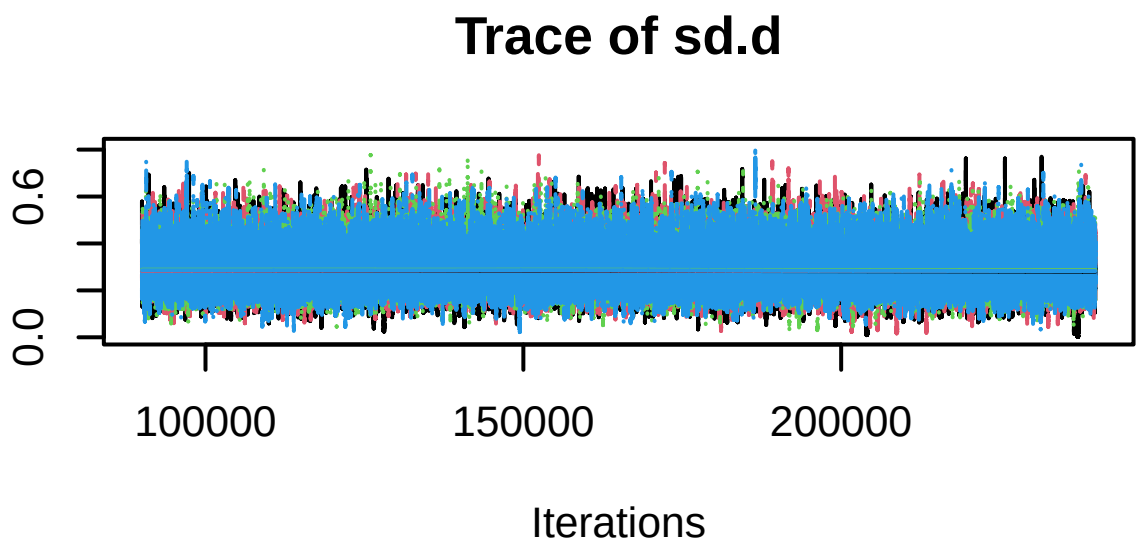
Trace of sd.d
0.0
0.6
100000
150000
200000
Iterations

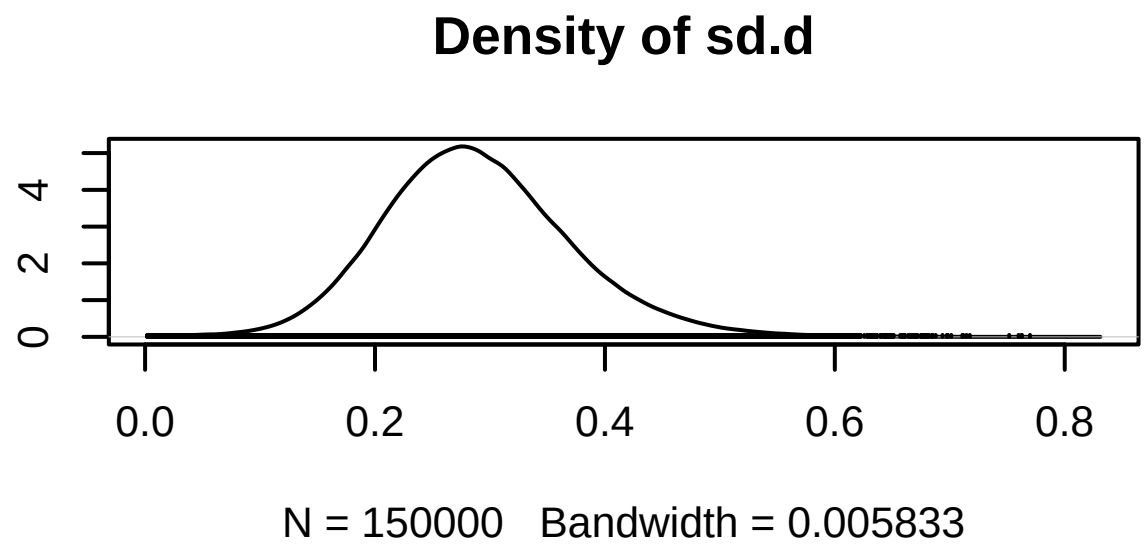
Density of sd.d
0
2
4
0.0
0.2
0.4
0.6
0.8
N = 150000   Bandwidth = 0.005833
