# Supplementary material for: Comparative safety of interleukin inhibitors for rheumatoid arthritis: a systematic review and network meta-analysis of randomized controlled trials
Source: Front Immunol. 2026 Sep 23;17:1866093. doi: 10.3389/fimmu.2026.1866093 (PMC13642689; doi:10.3389/fimmu.2026.1866093)
Supplement: Supplementary file 15 [file DataSheet9.pdf]

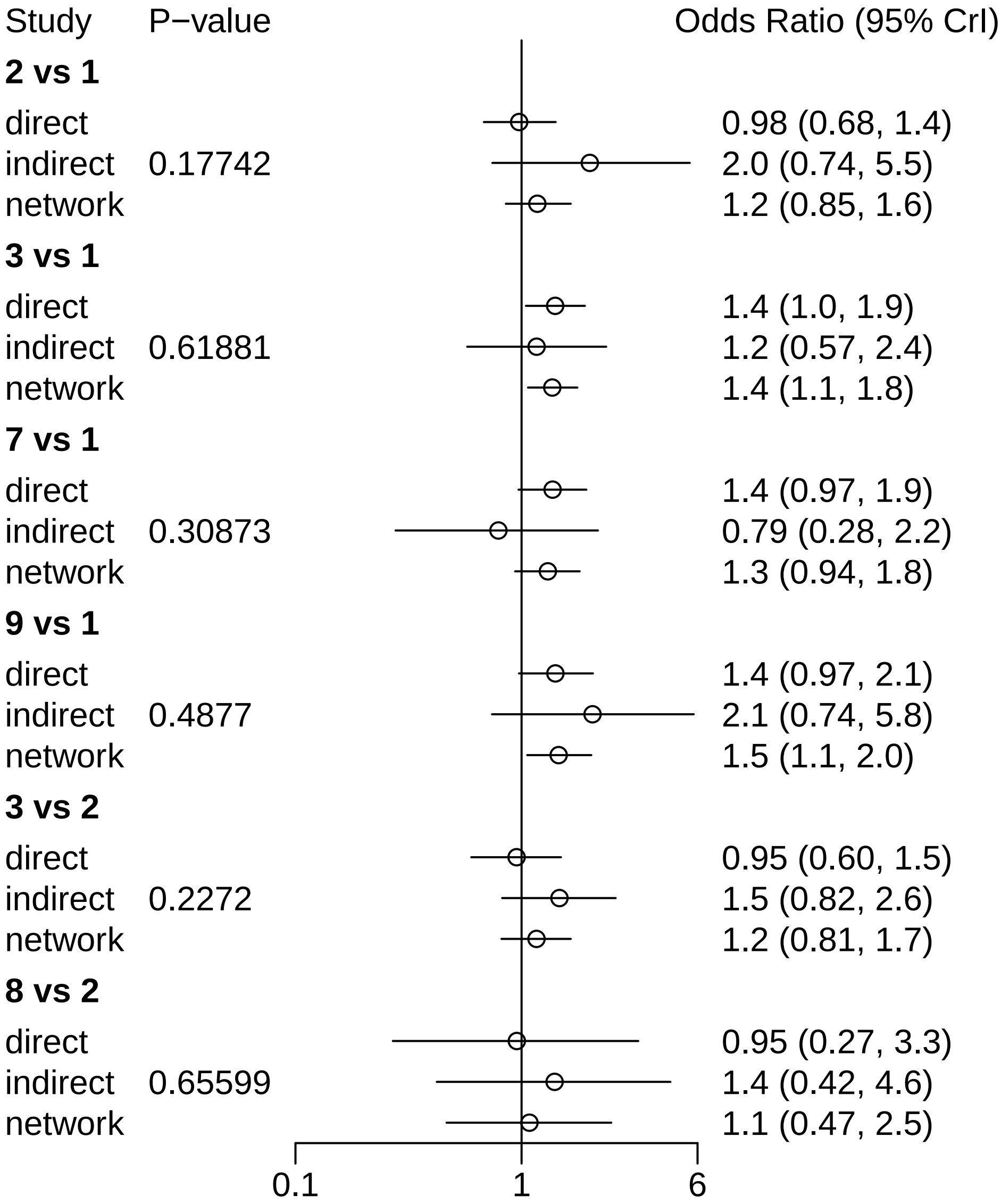
Study
P−value
Odds Ratio (95% CrI)
2 vs 1
direct
0.98 (0.68, 1.4)
indirect
0.17742
2.0 (0.74, 5.5)
network
1.2 (0.85, 1.6)
3 vs 1
direct
1.4 (1.0, 1.9)
indirect
0.61881
1.2 (0.57, 2.4)
network
1.4 (1.1, 1.8)
7 vs 1
direct
1.4 (0.97, 1.9)
indirect
0.30873
0.79 (0.28, 2.2)
network
1.3 (0.94, 1.8)
9 vs 1
direct
1.4 (0.97, 2.1)
indirect
0.4877
2.1 (0.74, 5.8)
network
1.5 (1.1, 2.0)
3 vs 2
direct
0.95 (0.60, 1.5)
indirect
0.2272
1.5 (0.82, 2.6)
network
1.2 (0.81, 1.7)
8 vs 2
direct
0.95 (0.27, 3.3)
indirect
0.65599
1.4 (0.42, 4.6)
network
1.1 (0.47, 2.5)
0.1
1
6

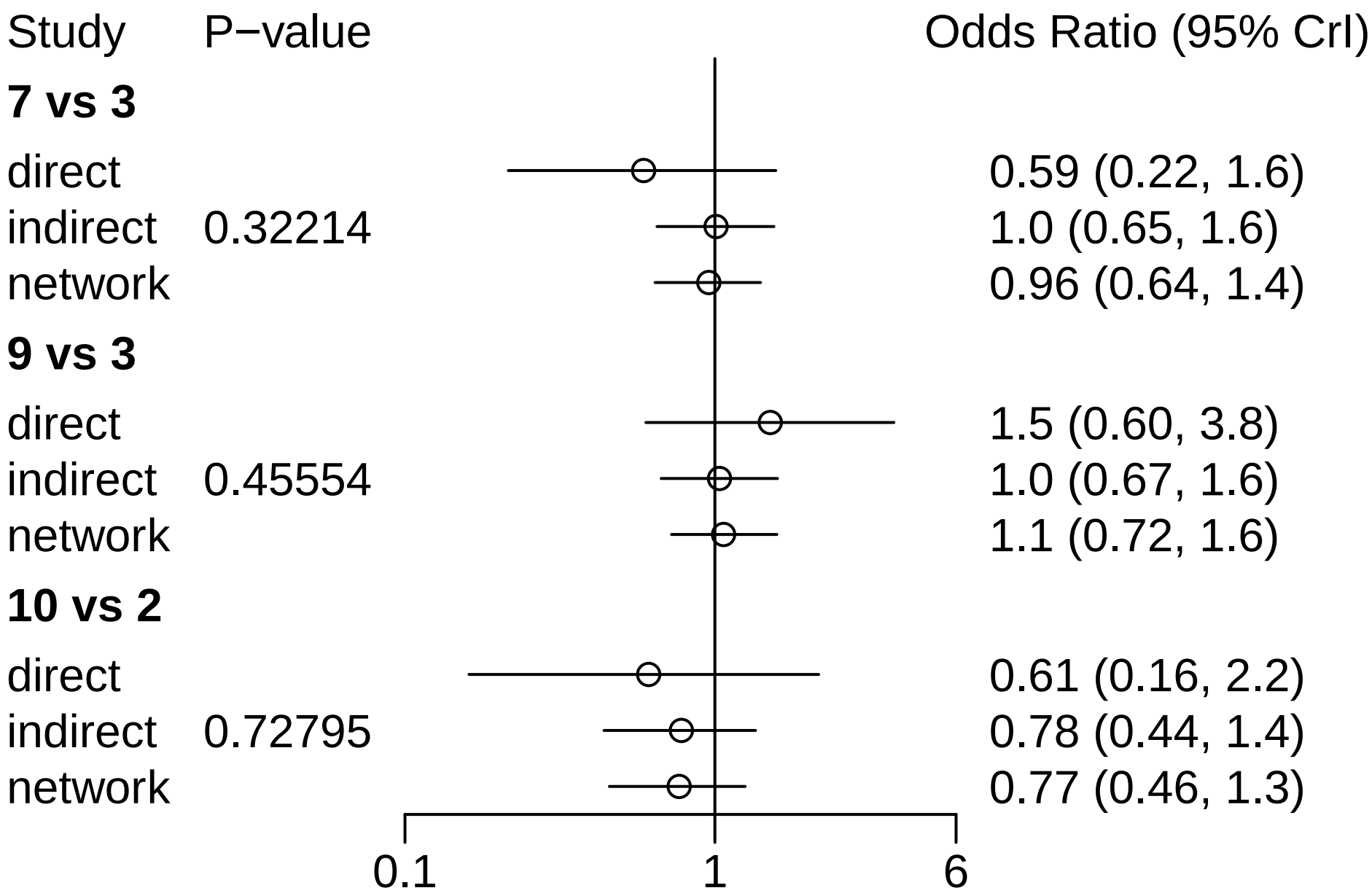
Study
P−value
Odds Ratio (95% CrI)
7 vs 3
direct
0.59 (0.22, 1.6)
indirect
0.32214
1.0 (0.65, 1.6)
network
0.96 (0.64, 1.4)
9 vs 3
direct
1.5 (0.60, 3.8)
indirect
0.45554
1.0 (0.67, 1.6)
network
1.1 (0.72, 1.6)
10 vs 2
direct
0.61 (0.16, 2.2)
indirect
0.72795
0.78 (0.44, 1.4)
network
0.77 (0.46, 1.3)
0.1
1
6
