# Supplementary material for: Comparative safety of interleukin inhibitors for rheumatoid arthritis: a systematic review and network meta-analysis of randomized controlled trials
Source: Front Immunol. 2026 Sep 23;17:1866093. doi: 10.3389/fimmu.2026.1866093 (PMC13642689; doi:10.3389/fimmu.2026.1866093)
Supplement: Supplementary file 16 [file Table1.docx]

| Table S1. Search strategy | |
| --- | --- |
| search database | Search terms |
| PubMed, Cochrane library, CNKI and clinicaltrials.gov | #1 Arthritis, Rheumatoid[Mesh]  #2 Rheumatoid arthritis  #3 Interleukin inhibitor  #4 anakinra  #5 olokizumab  #6 sarilumab  #7 tocilizumab  #8 Randomized controlled trial  #9 #1 or #2  #10 #3 or #4 or #5 or #6 or #7  #6 #8 and #9 and #10 |
| Embase | #1 'rheumatoid arthritis'/exp  #2 'arthritis deformans':ti,ab,kw OR 'arthritis, rheumatoid':ti,ab,kw OR 'arthrosis deformans':ti,ab,kw OR 'beauvais disease':ti,ab,kw OR 'chronic articular rheumatism':ti,ab,kw OR 'chronic polyarthritis':ti,ab,kw OR 'chronic rheumatoid arthritis':ti,ab,kw OR 'disease, beauvais':ti,ab,kw OR 'infantile rheumatoid arthritis':ti,ab,kw OR 'inflammatory arthritis':ti,ab,kw OR 'polyarthritis rheumatica':ti,ab,kw OR 'polyarthritis, primary chronic':ti,ab,kw OR 'primary chronic polyarthritis':ti,ab,kw OR 'rheumarthritis':ti,ab,kw OR 'rheumatic arthritis':ti,ab,kw OR 'rheumatic polyarthritis':ti,ab,kw OR 'rheumatism, chronic articular':ti,ab,kw OR 'rheumatoid polyarthritis':ti,ab,kw OR 'rheumatoid arthritis':ti,ab,kw  #3 'cytokine receptor antagonist'/exp  #4 'cytokine antagonist':ti,ab,kw OR 'cytokine receptor blocker':ti,ab,kw OR 'cytokine receptor blocking agent':ti,ab,kw OR 'interleukin inhibitor':ti,ab,kw OR 'interleukin inhibitors':ti,ab,kw OR 'interleukin receptor antagonist':ti,ab,kw OR 'interleukin receptor antagonists':ti,ab,kw OR 'cytokine receptor antagonist':ti,ab,kw  #5 anakinra  #6 olokizumab  #7 sarilumab  #8 tocilizumab  #9 Randomized controlled trial  #10 #1 or #2  #11 #3 or #4 or #5 or #6 or #6 or #8  #12 #10 and #11 and #9 |
