# Supplementary material for: Comparative safety of interleukin inhibitors for rheumatoid arthritis: a systematic review and network meta-analysis of randomized controlled trials
Source: Front Immunol. 2026 Sep 23;17:1866093. doi: 10.3389/fimmu.2026.1866093 (PMC13642689; doi:10.3389/fimmu.2026.1866093)
Supplement: Supplementary file 18 [file Table3.docx]

| Study | Trial registration number | Trial registration name | Failure of basic drugs |
| --- | --- | --- | --- |
| G Jones (2010) (18) | NCT00109408 | AMBITION | no failure with MTX or DMARDs |
| R. N. Maini (2006) (19) | - | CHARISMA | inadequate response to MTX |
| P Emery (2008) (20) | NCT00106522 | RADIATE | failed at least one TNF antagonist |
| Mark C. Genovese (2008) (21) | - | TOWARD | inadequate response to DMARDs |
| Norihiro Nishimoto (2009) (22) | NCT00144521 | SATORI | inadequate response to MTX |
| ALAN KIVITZ (2014) (23) | NCT01232569 | BREVACTA | inadequate response to >1 DMARDs |
| Tian Liu (2025) (24) | NCT03155347 | CONSORT | failure of at least 1 DMARDs |
| Johannes W J Bijlsma (2016) (25) | NCT01034137 | U-Act-Early | DMARD-naive |
| Yusuf Yazici (2012) (26) | NCT00531817 | ROSE | anti-TNF or non-biological DMARD |
| Joel M. Kremer (2018) (27) | NCT01855789 | COMPACT | inadequate response to MTX |
| Maxime Dougados (2013) (28) | NCT00810199 | ACT-RAY | inadequate response to MTX |
| Josef S Smolen (2008) (29) | NCT00106548 | OPTION | inadequate response to MTX |
| Han Joo Baek (2019) (30) | NCT01211834 | - | inadequate response to DMARDs |
| J.L. Pablos (2019) (31) | NCT01399697, | JUST-ACT | inadequate response to MTX |
| Gerd R Burmester (2016) (32) | NCT01007435 | FUNCTION | not received MTX or biological agents |
| Kristina Lend (2024) (33) | NCT01491815 | NORD-STAR | DMARD-naïve |
| JOHN TESSER (2004) (34) | - | - | inadequate response to DMARDs |
| Roy M. Fleischmann (2003) (35) | - | - | No restrictions |
| S B Cohen (2004) (36) | - | - | No restrictions |
| Michael H. Schiff (2004) (37) | - | - | inadequate response to MTX |
| Stanley Cohen (2002) (38) | - | - | inadequate response to MTX |
| Mark C Genovese (2013) (39) | NCT01242488 | - | intolerance to TNF Inhibitors |
| Eugen Feist (2022) (40) | NCT02760433 | CREDO 3 | intolerance to TNF Inhibitors |
| Tsutomu Takeuchi (2016) (41) | NCT01463059 | - | intolerance to TNF Inhibitors |
| Josef S. Smolen (2022) (42) | NCT02760407 | CREDO2 | inadequate response to MTX |
| Evgeniy Nasonov (2021) (43) | NCT02760368 | - | inadequate response to MTX |
| Mark C. Genovese (2020) (44) | NCT02332590 | MONARCH | inadequate response to MTX |
| Tom W J Huizinga (2013) (45) | NCT01061736 | MOBILITY(A) | inadequate response to MTX |
| Roy Fleischmann (2017) (46) | NCT01709578 | TARGET | intolerance to TNF Inhibitors |
| Mark C. Genovese (2015) (47) | NCT01061736 | MOBILITY(B) | inadequate response to MTX |
| Paul Emery (2019) (48) | NCT01768572 | ASCERTAIN | intolerance to TNF Inhibitors |
| Yoshiya Tanaka (2019) (49) | NCT02293902 | KAKEHASI | inadequate response to MTX |

MTX, Methotrexate; DMARDs, disease-modifying antirheumatic drugs; TNF, Tumor Necrosis Factor
