# Supplementary material for: Comparative safety of interleukin inhibitors for rheumatoid arthritis: a systematic review and network meta-analysis of randomized controlled trials
Source: Front Immunol. 2026 Sep 23;17:1866093. doi: 10.3389/fimmu.2026.1866093 (PMC13642689; doi:10.3389/fimmu.2026.1866093)
Supplement: Supplementary file 19 [file Table4.docx]

| **Table S2. Direct comparison of meta-analysis results** | | | | | | | | | | | | | | | |
| --- | --- | --- | --- | --- | --- | --- | --- | --- | --- | --- | --- | --- | --- | --- | --- |
| AE | Comparison category | | | | Number of studies | | | Heterogeneity | | | Meta analysis results | | | | |
|  |  |  |  |  |  |  |  | *I^2^* | | *P* | OR(95%CI) | | | | *P* |
|  | TCZ vs MTX | | | | 8 | | | 0.0% | | 0.980 | 1.05 (0.92, 1.21) | | | | 0.407 |
|  | TCZ + MTX vs MTX | | | | 10 | | | 18.5% | | 0.273 | **1.16(1.05, 1.29)** | | | | **0.004** |
|  | SAR(150mg) + MTX vs MTX | | | | 5 | | | 0.0% | | 0.985 | **1.23(1.07, 1.40)** | | | | **0.002** |
|  | OKZ(60mg) vs MTX | | | | 5 | | | 0.0% | | 0.761 | 1.14(0.95, 1.36) | | | | 0.159 |
|  | SAR(200mg) + MTX vs MTX | | | | 5 | | | 0.0% | | 0.996 | **1.26(1.10, 1.43)** | | | | **<0.0001** |
|  | OKZ(240mg) vs MTX | | | | 2 | | | 0.0% | | 0.961 | 1.01(0.55, 1.82) | | | | 0.975 |
|  | ANA vs MTX | | | | 2 | | | 0.0% | | 0.992 | 0.99(0.84, 1.17) | | | | 0.966 |
|  | ANA + MTX vs MTX | | | | 2 | | | **68.5%** | | **0.075** | 1.71(0.54, 5.38) | | | | 0.356 |
|  | SAR(200mg) vs MTX | | | | 1 | | | NA | | NA | 1.11(0.84, 1.46) | | | | 0.282 |
|  | Overall | | | | - | | | 0.0% | | 0.963 | **1.15(1.09, 1.21)** | | | | **<0.0001** |
| SAE |  | | | |  | | |  | |  |  | | | |  |
|  | TCZ vs MTX | | | | 8 | | | 0.0% | | 0.460 | 1.21(0.84, 1.74) | | | | 0.286 |
|  | TCZ + MTX vs MTX | | | | 9 | | | 10.7% | | 0.449 | 1.26(0.97, 1.62) | | | | 0.073 |
|  | ANA vs MTX | | | | 3 | | | 0.0% | | 0.999 | 0.98(0.81, 1.19) | | | | 0.891 |
|  | SAR(150mg) + MTX vs MTX | | | | 4 | | | 0.0% | | 0.502 | **1.50(1.05, 2.13)** | | | | **0.023** |
|  | SAR(200mg) + MTX vs MTX | | | | 4 | | | 0.0% | | 0.415 | **1.91(1.36, 2.68)** | | | | **<0.0001** |
|  | OKZ(60mg) vs MTX | | | | 3 | | | 0.0% | | 0.885 | 0.81(0.41, 1.60) | | | | 0.555 |
|  | OKZ(240mg) vs MTX | | | | 2 | | | 0.0% | | 0.609 | 1.00(0.55, 1.82) | | | | 0.954 |
|  | SAR(200mg) vs MTX | | | | 1 | | | NA | | NA | 1.02(0.47, 2.22) | | | | 0.945 |
|  | ANA + MTX vs MTX | | | | 1 | | | NA | | NA | 1.25(0.48, 3.23) | | | | 0.638 |
|  | Overall | | | | - | | | 2.0% | | 0.435 | **1.22(1.08, 1.38)** | | | | **0.001** |
|  | | | | | | | | | | | | | | | |
| Infection |  | | | |  | | |  | |  |  | | | |  |
|  | TCZ vs MTX | | | | 5 | | | 0.0% | | 0.887 | 0.984(0.81, 1.19) | | | | 0.869 |
|  | SAR(150mg) + MTX vs MTX | | | | 5 | | | 0.0% | | 0.418 | **1.25(1.06, 1.48)** | | | | **0.007** |
|  | OKZ(60mg) vs MTX | | | | 5 | | | 0.0% | | 0.698 | 0.94(0.74, 1.19) | | | | 0.627 |
|  | TCZ + MTX vs MTX | | | | 7 | | | **48.9%** | | **0.068** | **1.20(1.00, 1.45)** | | | | **0.049** |
|  | SAR(200mg) + MTX vs MTX | | | | 5 | | | 0.0% | | 0.939 | **1.24(1.05, 1.46)** | | | | **0.014** |
|  | ANA vs MTX | | | | 3 | | | 0.0% | | 0.867 | 0.92(0.79, 1.08) | | | | 0.321 |
|  | OKZ(240mg) vs MTX | | | | 2 | | | 26.6% | | 0.243 | 1.14(0.45, 2.86) | | | | 0.776 |
|  | SAR(200mg) vs MTX | | | | 1 | | | NA | | NA | 0.98(0.69, 1.40) | | | | 0.935 |
|  | Overall | | | | - | | | 8.7% | | 0.325 | **1.10(1.02, 1.18)** | | | | **0.007** |
| Serious infection |  | | | |  | | |  | |  |  | | | |  |
|  | TCZ vs MTX | | | | 5 | | | 0.0% | | 0.922 | 1.61 (0.71, 3.61) | | | | 0.247 |
|  | TCZ + MTX vs MTX | | | | 6 | | | 0.0% | | 0.785 | 1.43(0.87, 2.34) | | | | 0.125 |
|  | ANA vs MTX | | | | 3 | | | 0.0% | | 0.473 | 2.59(0.77, 8.67) | | | | 0.122 |
|  | OKZ(240mg) vs MTX | | | | 2 | | | 0.0% | | 0.862 | 2.60(0.47, 14.13) | | | | 0.268 |
|  | OKZ(60mg) vs MTX | | | | 4 | | | 0.0% | | 0.791 | 0.84(0.33, 2,16) | | | | 0.730 |
|  | SAR(200mg) vs MTX | | | | 1 | | | NA | | NA | 0.43(0.09, 1.98) | | | | 0.281 |
|  | SAR(200mg) + MTX vs MTX | | | | 1 | | | NA | | NA | 1.50(0.66, 3.38) | | | | 0.325 |
|  | SAR(150mg) + MTX vs MTX | | | | 1 | | | NA | | NA | 1.08(0.45.2.59) | | | | 0.847 |
|  | Overall | | | | - | | | 0.0% | | 0.972 | **1.35(1.00, 1.83)** | | | | **0.046** |
|  | | | | | | | | | | | | | | | |
| AE lead to discontinued | TCZ vs MTX | | | | 6 | | | 0.0% | | 0.774 | **1.52(1.03, 2.23)** | | | | **0.034** |
|  | TCZ + MTX vs MTX | | | | 7 | | | 0.0% | | 0.737 | **2.02(1.45, 2.81)** | | | | **<0.0001** |
|  | SAR(200mg) + MTX vs MTX | | | | 5 | | | 0.0% | | 0.959 | **2.55(1.85, 3.53)** | | | | **<0.0001** |
|  | SAR(150mg) + MTX vs MTX | | | | 5 | | | 0.0% | | 0.842 | **2.29 (1.65, 3.18)** | | | | **<0.0001** |
|  | OKZ(60mg) vs MTX | | | | 2 | | | 0.0% | | 0.569 | 1.57(0.77, 3.20) | | | | 0.214 |
|  | ANA+MTX vs MTX | | | | 2 | | | 0.0% | | 0.563 | 1.15(0.75, 1.76) | | | | 0.499 |
|  | SAR(200mg) vs MTX | | | | 1 | | | NA | | NA | 1.27(0.62, 2.58) | | | | 0.501 |
|  | ANA vs MTX | | | | 1 | | | NA | | NA | **0.37(0.22, 0.62)** | | | | **<0.0001** |
|  | OKZ(240mg) vs MTX | | | | 1 | | | NA | | NA | 1.15(0.14,8.49) | | | | 0.916 |
|  | Overall | | | | - | | | **49.3%** | | **0.001** | **1.65(1.32, 2.06)** | | | | **<0.0001** |
|  | | | | | | | | | | | | | | | |
| General disorders and administration site conditions | | OKZ(60mg) vs MTX | 4 | | | | 0.0% | | 0.921 | | | **2.27(1.14, 4.51)** | | **0.019** | |
|  |  | ANA vs MTX | 3 | | | | 0.0% | | 0.477 | | | **2.19(1.80, 2.66)** | | **<0.0001** | |
|  |  | TCZ vs MTX | 2 | | | | 0.0% | | 0.691 | | | 0.91(0.52, 1.57) | | 0.735 | |
|  |  | TCZ + MTX vs MTX | 4 | | | | 0.0% | | 0.264 | | | 1.27(0.91, 1.77) | | 0.146 | |
|  |  | OKZ(240mg) vs MTX | 2 | | | | 32.2% | | 0.225 | | | 2.71(0.75, 9.84) | | 0.128 | |
|  |  | ANA + MTX vs MTX | 2 | | | | 0.0% | | 0.477 | | | **2.76(1.97, 3.86)** | | **<0.0001** | |
|  |  | SAR(150mg) + MTX vs MTX | 1 | | | | NA | | NA | | | 3.66(0.98, 13.63) | | 0.052 | |
|  |  | SAR(200mg) + MTX vs MTX | 1 | | | | NA | | NA | | | **4.05(1.10, 14.89)** | | **0.035** | |
|  |  | Overall | - | | | | **44.1%** | | **0.021** | | | **1.85(1.49, 2.29)** | | **<0.0001** | |
|  | | | | | | | | | | | | | | | |
| Gastrointestinal disorders in AE | | TCZ vs MTX | 4 | | | | **77.8%** | | **0.004** | | | 2.67(0.83, 8.59) | | 0.098 | |
|  |  | OKZ(60mg) vs MTX | 4 | | | | 0.0% | | 0.810 | | | 1.07(0.55, 2.10) | | 0.832 | |
|  |  | TCZ + MTX vs MTX | 4 | | | | 0.0% | | 0.323 | | | **1.42(1.17, 1.72)** | | **<0.0001** | |
|  |  | OKZ(240mg) vs MTX | 2 | | | | 31.7% | | 0.226 | | | 1.87(0.54, 6.42) | | 0.319 | |
|  |  | SAR(150mg) + MTX vs MTX | 1 | | | | NA | | NA | | | 0.90(0.36, 2.25) | | 0.837 | |
|  |  | SAR(200mg) + MTX vs MTX | 1 | | | | NA | | NA | | | 1.84(0.82, 4.08) | | 0.134 | |
|  |  | Overall | - | | | | 27.0% | | 0.187 | | | **1.38(1.04, 1.83)** | | **0.002** | |
|  | | | | | | | | | | | | | | | |
| Neurological disorders in AE | | TCZ + MTX vs MTX | | 3 | | 0.0% | | | 0.553 | | | 1.22(0.92, 1.62) | 0.151 | | |
|  |  | OKZ(60mg) vs MTX | | 2 | | 0.0% | | | 0.779 | | | 1.30(0.48, 3.50) | 0.595 | | |
|  |  | TCZ vs MTX | | 1 | | NA | | | NA | | | **2.02(1.12, 3.64)** | **0.018** | | |
|  |  | OKZ(240mg) vs MTX | | 1 | | NA | | | NA | | | 0.66 (0.14, 3.07) | 0.606 | | |
|  |  | Overall | | - | | 0.0% | | | 0.742 | | | **1.32(1.03, 1.68)** | **0.024** | | |
|  | | | | | | | | | | | | | | | |
| Disorders of the skin and subcutaneous tissues in AE | | TCZ + MTX vs MTX | | 4 | | 37.2% | | | 0.207 | | | **2.16(1.68, 2.78)** | **<0.0001** | | |
|  |  | OKZ(60mg) vs MTX | | 2 | | 0.0% | | | 0.537 | | | 2.32(0.68, 7.90) | 0.175 | | |
|  |  | TCZ vs MTX | | 2 | | 0.0% | | | 0.868 | | | 1.27(0.81, 1.97) | 0.285 | | |
|  |  | OKZ(240mg) vs MTX | | 1 | | NA | | | NA | | | 0.74(0.11, 4.80) | 0.756 | | |
|  |  | SAR(200mg) + MTX vs MTX | | 1 | | NA | | | NA | | | 1.51(0.58, 3.91) | 0.387 | | |
|  |  | Overall | | - | | 12.2% | | | 0.337 | | | **1.86(1.52, 2.28)** | **<0.0001** | | |
|  | | | | | | | | | | | | | | | |
| Musculoskeletal and connective tissue disorders in AE | | TCZ + MTX vs MTX | | 3 | | 0.0% | | | 0.998 | | | **0.73(0.57, 0.93)** | **0.014** | | |
|  |  | TCZ vs MTX | | 2 | | 0.0% | | | 0.991 | | | 0.97(0.62, 1.52) | 0.918 | | |
|  |  | SAR(200mg) + MTX vs MTX | | 1 | | NA | | | NA | | | 0.50(0.11, 2.10) | 0.345 | | |
|  |  | SAR(150mg) + MTX vs MTX | | 1 | | NA | | | NA | | | 0.16(0.01, 1.40) | 0.099 | | |
|  |  | OKZ(60mg) vs MTX | | 1 | | NA | | | NA | | | 0.90(0.40, 2.01) | 0.807 | | |
|  |  | Overall | | - | | 0.0% | | | 0.599 | | | **0.77(0.62, 0.95)** | **0.014** | | |
|  | | | | | | | | | | | | | | | |
| Vascular disorders in AE | | TCZ + MTX vs MTX | | 3 | | 0.0% | | | 0.898 | | | 1.34(0.90, 2.00) | 0.141 | | |
|  |  | TCZ vs MTX | | 1 | | NA | | | NA | | | 1.82(0.90, 3.64) | 0.091 | | |
|  |  | OKZ(60mg) vs MTX | | 1 | | NA | | | NA | | | 0.43(0.08, 2.19) | 0.310 | | |
|  |  | SAR(200mg) + MTX vs MTX | | 1 | | NA | | | NA | | | 5.06(0.57, 44.28) | 0.143 | | |
|  |  | SAR(150mg) + MTX vs MTX | | 1 | | NA | | | NA | | | 4.00(0.43, 36.55) | 0.219 | | |
|  |  | Overall | | - | | 0.0% | | | 0.545 | | | **1.45(1.04, 2.02)** | **0.026** | | |
|  | | | | | | | | | | | | | | | |
| Nasopharyngitis in infection | | TCZ vs MTX | | 2 | | 0.0% | | | 0.569 | | | 1.29(0.74, 2.25) | 0.369 | | |
|  |  | SAR(150mg) + MTX vs MTX | | 2 | | 0.0% | | | 0.548 | | | 1.08(0.48, 2.43) | 0.288 | | |
|  |  | SAR(200mg) + MTX vs MTX | | 3 | | 0.0% | | | 0.489 | | | 1.32(0.81, 2.16) | 0.261 | | |
|  |  | OKZ(60mg) vs MTX | | 2 | | 19.8% | | | 0.264 | | | 0.84(0.24, 2.92) | 0.793 | | |
|  |  | SAR(200mg) vs MTX | | 1 | | NA | | | NA | | | 0.76(0.27, 2.09) | 0.603 | | |
|  |  | OKZ(240mg) vs MTX | | 1 | | NA | | | NA | | | 3.34(0.62, 18.05) | 0.160 | | |
|  |  | TCZ + MTX vs MTX | | 1 | | NA | | | NA | | | 1.20(0.50,2.84) | 0.678 | | |
|  |  | Overall | | - | | 0.0% | | | 0.930 | | | 1.24 (0.96, 1.58) | 0.089 | | |
|  | | | | | | | | | | | | | | | |
| Upper respiratory tract infection in infection | | TCZ vs MTX | | 3 | | 0.0% | | | 0.807 | | | 1.25(0.76, 2.04) | 0.363 | | |
|  |  | SAR(150mg) + MTX vs MTX | | 3 | | 24.3% | | | 0.267 | | | 1.07(0.52, 2.18) | 0.849 | | |
|  |  | ANA vs MTX | | 2 | | 0.0% | | | 0.559 | | | 0.69(0.46, 1.05) | 0.085 | | |
|  |  | SAR(200mg) + MTX vs MTX | | 2 | | 0.0% | | | 0.855 | | | 1.52(0.92, 2.52) | 0.098 | | |
|  |  | TCZ + MTX vs MTX | | 3 | | 52.9% | | | 0.120 | | | **1.89(1.09, 3.29)** | **0.023** | | |
|  |  | SAR(200mg) vs MTX | | 1 | | NA | | | NA | | | 0.98(0.31, 3.10) | 0.978 | | |
|  |  | Overall | | - | | **46.3%** | | | **0.029** | | | 1.23(0.92, 1.63) | 0.151 | | |
|  | | | | | | | | | | | | | | | |
| Cellulitis in serious infection | | OKZ(240mg) vs MTX | | 2 | | 0.0% | | | 0.793 | | | 1.47(0.23, 9.34) | 0.682 | | |
|  |  | SAR(200mg) + MTX vs MTX | | 2 | | 0.0% | | | 0.824 | | | 2.47(0.47, 12.96) | 0.284 | | |
|  |  | TCZ vs MTX | | 1 | | NA | | | NA | | | 1.97(0.35, 10.85) | 0.435 | | |
|  |  | TCZ + MTX vs MTX | | 1 | | NA | | | NA | | | 3.10(0.37, 25.84) | 0.296 | | |
|  |  | SAR(150mg) + MTX vs MTX | | 1 | | NA | | | NA | | | 4.00(0.43, 36.55) | 0.219 | | |
|  |  | Overall | | - | | 0.0% | | | 0.994 | | | **2.33(1.01, 5.39)** | **0.047** | | |
|  | | | | | | | | | | | | | | | |
| Pneumonia in serious infection | | TCZ vs MTX | | 3 | | 0.0% | | | 0.796 | | | 0.90(0.48, 1.71) | 0.766 | | |
|  |  | TCZ + MTX vs MTX | | 2 | | 0.0% | | | 0.819 | | | 0.94(0.50, 1.76) | 0.853 | | |
|  |  | OKZ(240mg) vs MTX | | 2 | | 32.1% | | | 0.225 | | | 0.64(0.10, 4.02) | 0.638 | | |
|  |  | ANA vs MTX | | 2 | | 0.0% | | | 1.000 | | | 0.48(0.08, 2.67) | 0.407 | | |
|  |  | OKZ(60mg) vs MTX | | 1 | | 0.0% | | | 0.752 | | | 0.90(0.05, 15.18) | 0.947 | | |
|  |  | Overall | | - | | 0.0% | | | 0.959 | | | 0.84(0.56, 1.26) | 0.409 | | |
|  | | | | | | | | | | | | | | | |
| Herpes zoster in serious infection | | TCZ vs MTX | | 2 | | 0.0% | | | 0.851 | | | 1.78(0.45, 7.00) | 0.404 | | |
|  |  | TCZ + MTX vs MTX | | 2 | | 0.0% | | | 0.431 | | | 1.20(0.21, 6.73) | 0.835 | | |
|  |  | SAR(150mg) + MTX vs MTX | | 1 | | NA | | | NA | | | 3.00(0.30, 29.44) | 0.346 | | |
|  |  | SAR(200mg) + MTX vs MTX | | 1 | | NA | | | NA | | | 1.01(0.06, 16.46) | 0.993 | | |
|  |  | OKZ(60mg) vs MTX | | 1 | | NA | | | NA | | | 1.00(0.06, 16.14) | 1.000 | | |
|  |  | Overall | | - | | 0.0% | | | 0.972 | | | 1.53(0.52, 4.47) | 0.318 | | |
|  | | | | | | | | | | | | | | | |
| Sepsis in serious infection | | TCZ vs MTX | | 1 | | NA | | | NA | | | 2.02(0.18, 22.49) | 0.565 | | |
|  |  | TCZ + MTX vs MTX | | 1 | | NA | | | NA | | | 2.06(0.23, 18.55) | 0.517 | | |
|  |  | OKZ(60mg) vs MTX | | 1 | | NA | | | NA | | | 1.00(0.06, 16.14) | 1.000 | | |
|  |  | Overall | | - | | 0.0% | | | 0.909 | | | 1.70(0.42, 6.93) | 0.454 | | |
|  | | | | | | | | | | | | | | | |
| Death | | SAR(200mg) + MTX vs MTX | | 5 | | 0.0% | | | 0.988 | | | 0.62 (0.20, 1.96) | 0.424 | | |
|  |  | OKZ(60mg) vs MTX | | 5 | | 0.0% | | | 0.992 | | | 0.84(0.25, 2.83) | 0.783 | | |
|  |  | SAR(150mg) + MTX vs MTX | | 5 | | 0.0% | | | 0.993 | | | 0.85(0.31, 2.44) | 0.798 | | |
|  |  | TCZ vs MTX | | 4 | | 0.0% | | | 0.954 | | | 0.49(0.14, 1.77) | 0.283 | | |
|  |  | TCZ + MTX vs MTX | | 5 | | 0.0% | | | 0.880 | | | 1.09(0.41, 2.89) | 0.851 | | |
|  |  | OKZ(240mg) vs MTX | | 2 | | 0.0% | | | 0.959 | | | 1.05(0.14, 7.77) | 0.959 | | |
|  |  | SAR(200mg) vs MTX | | 1 | | NA | | | NA | | | 1.07(0.09, 11.97) | 0.951 | | |
|  |  | ANA vs MTX | | 1 | | NA | | | NA | | | 1.01(0.11, 9.11) | 0.990 | | |
|  |  | Overall | | - | | 0.0% | | | 1.000 | | | 0.82(0.52, 1.30) | 0.414 | | |
|  | | | | | | | | | | | | | | | |
| Abnormal Library investigation | | SAR(150mg) + MTX vs MTX | | 4 | | 0.0% | | | 0.908 | | | **1.85(1.30, 2.68)** | **0.001** | | |
|  |  | SAR(200mg) + MTX vs MTX | | 4 | | 0.0% | | | 0.411 | | | **2.10(1.48, 2.97)** | **<0.0001** | | |
|  |  | TCZ + MTX vs MTX | | 3 | | 54.5% | | | 0.111 | | | **2.71(1.45, 5.09)** | **0.002** | | |
|  |  | OKZ(60mg) vs MTX | | 2 | | 0.0% | | | 0.818 | | | **2.01(1.25, 3.24)** | **0.004** | | |
|  |  | TCZ vs MTX | | 1 | | NA | | | NA | | | 1.10(0.70, 1.71) | 0.671 | | |
|  |  | Overall | | - | | 27.8% | | | 0.158 | | | **1.99(1.59, 2.48)** | **<0.0001** | | |
|  | | | | | | | | | | | | | | | |
| Injury, poisoning and procedural complications | | TCZ + MTX vs MTX | | 2 | | 0.0% | | | 0.939 | | | 1.11(0.75, 1.62) | 0.590 | | |
|  |  | TCZ vs MTX | | 1 | | NA | | | NA | | | 0.92(0.43, 1.94) | 0.828 | | |
|  |  | SAR(200mg) + MTX vs MTX | | 1 | | NA | | | NA | | | 1.00(0.30, 3.30) | 1.000 | | |
|  |  | SAR(150mg) + MTX vs MTX | | 1 | | NA | | | NA | | | 0.81(0.23, 2.84) | 0.751 | | |
|  |  | OKZ(60mg) vs MTX | | 1 | | NA | | | NA | | | 4.31(0.54, 34.34) | 0.167 | | |
|  |  | Overall | | - | | 0.0% | | | 0.830 | | | 1.07(0.79, 1.47) | 0.632 | | |
|  | | | | | | | | | | | | | | | |
| Neoplasms benign, malignant and unspecified | | TCZ vs MTX | | 3 | | 0.0% | | | 0.901 | | | 0.50(0.14, 1.76) | 0.281 | | |
|  |  | OKZ(60mg) vs MTX | | 2 | | 0.0% | | | 0.685 | | | 0.60(0.14, 2.43) | 0.475 | | |
|  |  | OKZ(240mg) vs MTX | | 1 | | NA | | | NA | | | 1.00(0.05, 17.01) | 1.000 | | |
|  |  | TCZ + MTX vs MTX | | 1 | | NA | | | NA | | | 0.32(0.03, 3.13) | 0.331 | | |
|  |  | Overall | | - | | 0.0% | | | 0.992 | | | 0.53(0.23, 1.22) | 0.137 | | |
|  | | | | | | | | | | | | | | | |
| ALT increasing in Library investigation | | SAR(150mg) + MTX vs MTX | | 5 | | 0.0% | | | 0.718 | | | **2.76(1.80, 4.24)** | **<0.0001** | | |
|  |  | SAR(200mg) + MTX vs MTX | | 5 | | 3.2% | | | 0.388 | | | **2.64(1.68, 4.15)** | **<0.0001** | | |
|  |  | TCZ vs MTX | | 2 | | 43.7% | | | 0.182 | | | 1.36(1.05, 1.76) | 0.425 | | |
|  |  | TCZ + MTX vs MTX | | 2 | | 0.0% | | | 0.728 | | | **1.18(1.52, 2.68)** | **0.017** | | |
|  |  | SAR(200mg) vs MTX | | 1 | | NA | | | NA | | | **2.87(1.19, 6.94)** | **0.019** | | |
|  |  | OKZ(60mg) vs MTX | | 1 | | NA | | | NA | | | **3.00(1.45, 6.16)** | **0.003** | | |
|  |  | Overall | | - | | **64.9%** | | | **<0.0001** | | | **1.88(1.36, 2.60)** | **<0.0001** | | |
|  | | | | | | | | | | | | | | | |
| AST increasing in Library investigation | | SAR(200mg) + MTX vs MTX | | 2 | | 0.0% | | | 0.524 | | | 2.16(0.65, 7.21) | 0.208 | | |
|  |  | SAR(150mg) + MTX vs MTX | | 2 | | 0.0% | | | 0.434 | | | 1.43(0.38, 5.31) | 0.591 | | |
|  |  | TCZ vs MTX | | 2 | | 21.5% | | | 0.259 | | | 0.80(0.35, 1.85) | 0.613 | | |
|  |  | TCZ + MTX vs MTX | | 2 | | NA | | | NA | | | **1.54(1.15, 2.06)** | **0.004** | | |
|  |  | Overall | | - | | 19.4% | | | 0.276 | | | 1.24(0.91, 1.68) | 0.158 | | |
| AE, adverse event; SAE, serious adverse event; Abbreviation, NA; CI, Confidence interval; OR, odds ratio. ALT, The bold font indicates that there was a statistically significant difference between the two treatments. | | | | | | | | | | | | | | | |
